# Synthesis of Aminoalkyl Sclareolide Derivatives and Antifungal Activity Studies

**DOI:** 10.3390/molecules28104067

**Published:** 2023-05-12

**Authors:** Ziyi Li, Hua Gao, Haibo Mei, Guangwei Wu, Vadim A. Soloshonok, Jianlin Han

**Affiliations:** 1 Jiangsu Co-Innovation Center of Efficient Processing and Utilization of Forest Resources, College of Chemical Engineering, Nanjing Forestry University, Nanjing 210037, China; 2 Ningbo Institute of Marine Medicines, Peking University, Ningbo 315010, China; 3 Department of Organic Chemistry I, Faculty of Chemistry, University of the Basque Country UPV/EHU, Paseo Manuel Lardizábal 3, 20018 San Sebastián, Spain; 4 IKERBASQUE, Basque Foundation for Science, Alameda Urquijo 36-5, Plaza Bizkaia, 48011 Bilbao, Spain

**Keywords:** sclareolide, Mannich reaction, aldmine, antifungal activity

## Abstract

Sclareolide was developed as an efficient *C*-nucleophilic reagent for an asymmetric Mannich addition reaction with a series of *N*-*tert*-butylsulfinyl aldimines. The Mannich reaction was carried out under mild conditions, affording the corresponding aminoalkyl sclareolide derivatives with up to 98% yield and 98:2:0:0 diastereoselectivity. Furthermore, the reaction could be performed on a gram scale without any reduction in yield and diastereoselectivity. Additionally, deprotection of the obtained Mannich addition products to give the target sclareolide derivatives bearing a free N-H group was demonstrated. In addition, target compounds **4**–**6** were subjected to an antifungal assay in vitro, which showed considerable antifungal activity against forest pathogenic fungi.

## 1. Introduction

Sclareolide belongs to a sesqui-terpene lactone type of organic compound, which is isolated from the flowers of Salvia sclarea, with various biological activities, such as antifungal, antibacterial, anticancer, anti-inflammatory, and cytotoxic effects [1]. For example, sclareolide showed anti-Ebola virus (EBOV) activity and can act as an EBOV fusion inhibitor, inhibiting the growth of eight filamentous viruses [2]. Furthermore, sclareolide is an important organic intermediate that has been widely used in the total synthesis of natural products and the preparation of bioactive compounds [3,4,5]. Thus, there exists a lot of interest in the development of efficient strategies for the modifications of sclareolide in organic and biological chemistry [6,7,8,9,10,11].

**Scheme 1:**
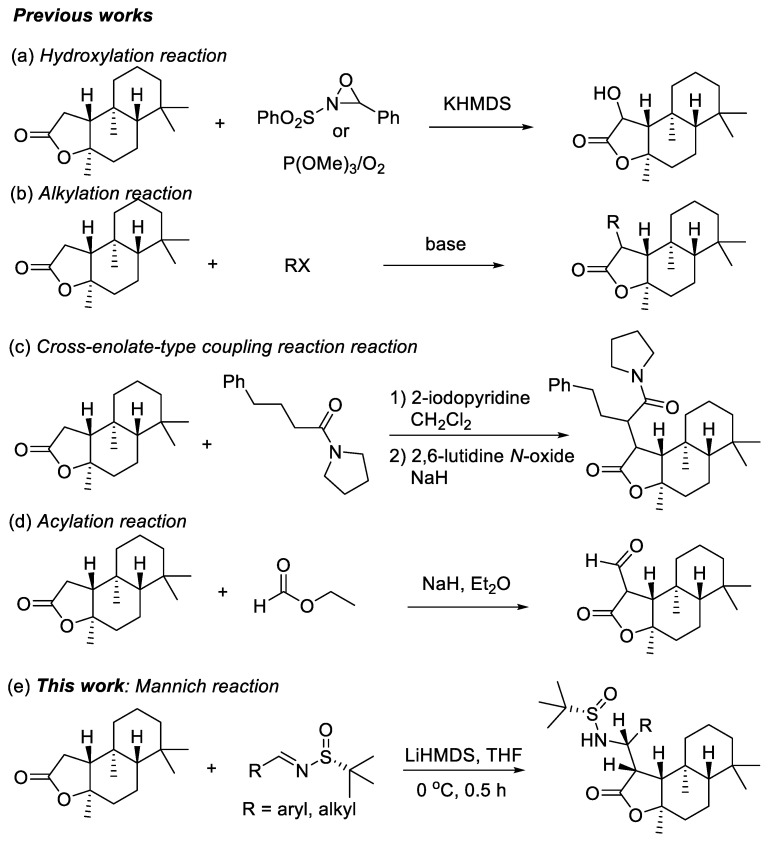
Modifications on α-C of sclareolide.

The synthesis of sclareolide-derived molecules has attracted a lot of attention in recent years, and several works have been developed, such as those on ring opening and selective C–H bond functionalization [12,13,14,15,16,17,18,19]. In particular, the modification of α-C of the lactone moiety of sclareolide was important due to the unique reactivity of this position [20,21,22,23,24,25,26,27]. The reaction of sclareolide with 2-benzenesulfonyl-3-phenyl-oxaziridine using KHMDS as a base could successfully introduce a hydroxyl group to the α-position of the lactone moiety [28]. The treatment of sclareolide with KHMDS, followed by a reaction with P(OMe)_3_ in an oxygen atmosphere, also provided hydroxylated sclareolide (Scheme 1a) [29]. On the other hand, the alkylation of sclareolide represents another modification, which usually uses sclareolide as an enolate to react with alkyl halide under the basic conditions (Scheme 1b) [30]. The cross-enolate-type coupling reaction of sclareolide with amide carbonyl could also realize alkylation via the use of 2-iodopyridine and 2,6-lutidine *N*-oxide in the presence of NaH (Scheme 1c) [31]. In the presence of sodium hydride, the α-formylation of sclareolide was achieved via a treatment with ethyl formate in ether (Scheme 1d) [32,33]. Despite there being excellent reports on the α-functionalization of lactone unit of sclareolide, the installation of amino functionality in this position still remains unexplored. As a result of our continuous interest in the development of the Mannich reaction with chiral *N*-*tert*-butylsulfinyl aldimines [34,35,36,37] and discovering new natural-product-derived molecules featuring antifungal activity against forest pathogenic fungi, herein, we would like to report an asymmetric Mannich reaction of *N*-*tert*-butylsulfinyl aldimines with sclareolide as an enolate precursor (Scheme 1e). The reaction was conducted under mild conditions, affording a series of new α-aminoalkyl sclareolide derivatives as products in excellent yields and high diastereoselectivities. Furthermore, these obtained new compounds were subjected to antifungal activity testing against two forest pathogenic fungi, *F. oxysporum* and *L. theobromae* [38], which showed good antifungal activities. 

## 2. Results and Discussion

Chiral sulfinamides/imines and their derivatives are relatively inexpensive reagents, allowing high levels of stereocontrol in corresponding addition reactions [39,40,41,42]. Thus, sclareolide (**1**) and (*R*)-*N*-benzylidene-2-methylpropane-2-sulfinamide (**2a**) were chosen as the model substrate for this asymmetric Mannich reaction. After a series of experiments, we used 1.2 equivalents of sclareolide and 0 °C as the starting point for the optimization of the reaction conditions. The reaction was performed at 0 °C using tetrahydrofuran as a solvent in the presence of LiHMDS. After four hours, the desired Mannich adduct, **3a,** was obtained in a moderate yield and excellent diastereoselectivity (69% yield, 92:8:0:0 dr; entry 1, Table 1). Then, several bases, including MeONa, LDA, and BuLi, were used for this reaction instead of LiHMDS. No improvement was obtained at all, and almost none of the desired product, **3a,** was observed when NaOMe and BuLi were used (entries 2 and 4). A significant effect of temperature on the reaction outcome was observed (entries 5 and 6), and the results disclose that 0 °C was the best choice. Subsequently, the screening of the reaction time showed that the reaction could be completed within a short time (0.5 h), with an obviously increased yield (95%) and a similar diastereoselectivity (entries 7, 8, and 9). Moreover, the reaction media also have a significant effect on this reaction outcome (entries 10, 11, and 12), and a dramatically decreased yield was observed in the reaction conducted with dichloromethane, acetonitrile, and 1,4-dioxane. Finally, the loading amount of sclareolide (**1**) was varied. Although no improvement in the chemical yield was observed, a slightly higher diastereoselectivity was obtained (entry 13). 

**Scheme 2:**
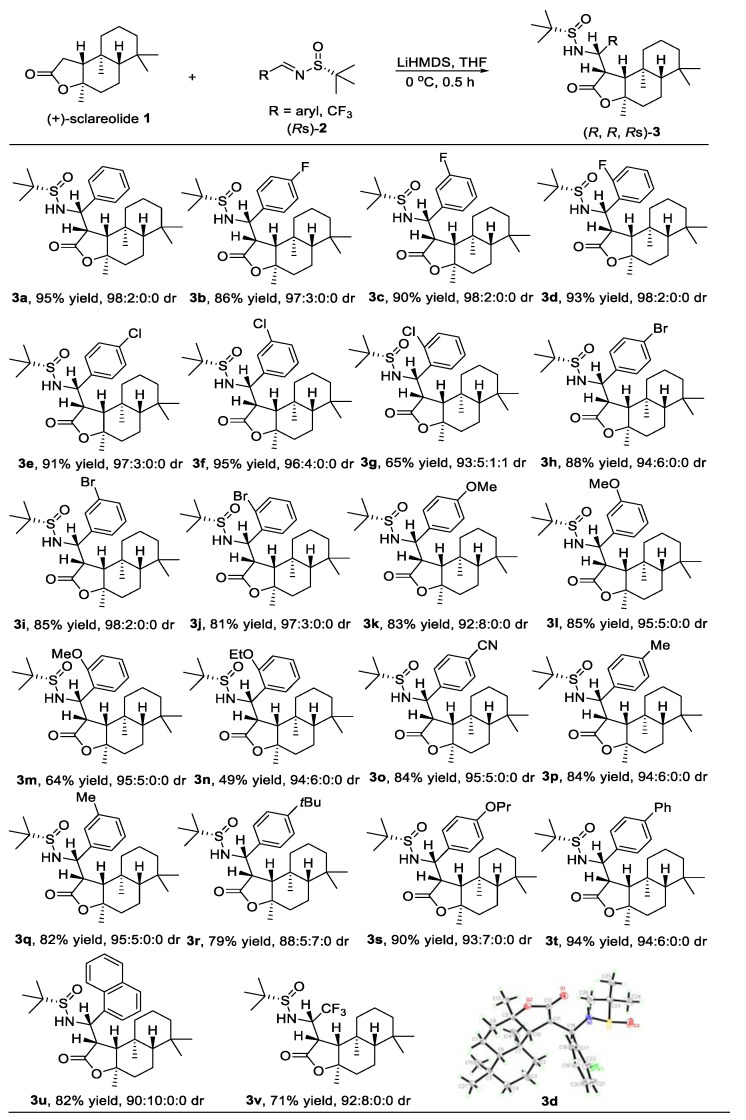
Substrate scope of various imines **2** (reaction conditions: sclareolide **1** (0.3 mmol), aldimine **2a** (0.2 mmol), and LiHMDS (0.45 mmol)). Isolated yields based on **2**.

With the optimized reaction conditions in hand, we then evaluated the substrate generality of this asymmetric Mannich reaction by using varieties of *N*-*tert*-butylsulfinyl aldimines **2** (Scheme 2). All of the tested forms of aldimine substrate **2** worked very well under the standard conditions, resulting in the corresponding aminoalkyl sclareolide products (**3a**–**v**) in moderate-to-high yields (49–95%) and high diastereoselectivities. The electronegativity of the substituent on the phenyl ring showed almost no obvious effect on the reaction outcome; even the substrates featuring a strong electron-donating substituent (OMe, **2k**) and electron-withdrawing substituent (CN, **2o**) were also tolerated very well to give the corresponding products, **3k** and **3b,** in 83% and 86% yields, respectively. Then, the influence of steric hindrance on the reaction was investigated. Usually, para- and meta-substituted phenyl-containing substrates worked better and reacted smoothly with sclareolide (**1**), affording product **3** with moderate-to-high yields (79–95%). However, a decreased yield was found for substrates with an ortho-substituted phenyl moiety (64% yields for **3g** and **3m**). A more bulky group, such as ethoxyl (**2n**), still could react with sclareolide, but with only 49% yield. In addition, the naphthalene ring-containing substrate also showed good reactivity with a high yield (**3u**, 82%) and good diastereoselectivity (dr = 90:10:0:0). As the fluoroalkyl group has been found in many bioactive molecules [43,44,45], an aldimine substrate with a fluoroalkyl moiety was examined in this asymmetric Mannich reaction. The reaction with aldimine **2v** proceeded smoothly, achieving the formation of the desired product, **3v,** in 71% yield and 92:8:0:0 diastereoselectivity. To determine the absolute configuration of the chiral addition product, **3**, we successfully performed crystallographic X-ray analysis of product **3d**, and the structure is shown in Scheme 2. The absolute configuration of the newly generated chiral centers in the main product, **3d**, are both (*R*, *R*) (For details, see Figure S1 in Supplementary Materials). The absolute configurations of other corresponding products, **3**, were assigned accordingly.

To demonstrate the scalability of this methodology, we conducted the reaction with the starting imine, **2a**, on a gram scale under standard conditions. The reaction with the amount of imine, **2a**, increased from 0.2 to 4.01 mmol. Fortunately, the gram-scale reaction also proceeded smoothly, yielding the desired product, **3a,** in a high yield (99%) and high diastereoselectivity (97:3:0:0). 

**Scheme 3:**
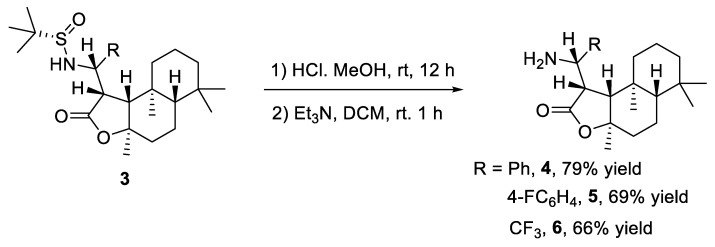
Removal of the chiral auxiliary.

Then, we conducted further chemical reactions with the obtained product **3** via the removal of the chiral auxiliary *tert*-butyl sulfinyl. Treating compound **3** with an aqueous solution of HCl (36%) in methanol was performed at room temperature for 12 h. Then, the obtained amine hydrochloride was neutralized with trimethylamine in dichloromethane at room temperature for 1 h (Scheme 3), affording free amines **4**–**6** with isolated yields of 79%, 69%, and 66%, respectively. 

To investigate whether the antifungal activity could be improved compared with that of sclareolide, compounds **4**–**6** were subjected to the examination of preliminary antifungal activity against two forest pathogenic fungi at 50.0 mg/L, and sclareolide (**1**) was used as a positive control (for details, see Figures S2 and S3 in Supplementary Materials). The results in Table 2 show that compounds **4** and **5** could effectively inhibit the growth of fungal mycelium. Compounds **4** and **5** showed 48% and 53% inhibition rates against *F. oxysporum*, which were about two-fold higher than that of sclareolide (20%). Similarly, for *L. theobromae*, compounds **4** and **5** showed 67% and 61% inhibition rates, which were also higher than that of sclareolide (53%) [46,47,48,49]. Compound **6** did not have a better inhibitory effect on *L. theobromae*.

## 3. Materials and Methods

### 3.1. General Information

All the commercial reagents, including solvents, were used directly without further purification. All the experiments were monitored via thin-layer chromatography (TLC) with UV light. For TLC, we employed 0.25 mm silica gel coated on glass plates. The purification of products was carried out on silica gel 60 F-254 TLC plates of 20 cm × 20 cm. Melting points were recorded without correction using RY -1G of Tianjin Xintianguang instrument company. NMR spectra were recorded with Bruker 400 MHz and 600 MHz spectrometers. High-resolution mass spectra (HRMS) were measured with Agilent 6210 ESI/TOF MS instrument. Values of optical rotation were measured using an automatic polarimeter SGW-531. X-ray data were collected at 100 K using a Rigaku Oxford Diffraction Supernova Dual Source, Cu at Zero, equipped with an AtlasS2 CCD using Cu Kα radiation.

### 3.2. General Procedure for the Mannich Reaction

Sclareolide (**1**) (0.3 mmol) and anhydrous THF (2.0 mL) were obtained from an oven-dried reaction vial flushed with N_2_. The reaction vial was cooled to 0 °C, and LiHMDS (1 M in THF, 0.45 mmol) was added dropwise and stirred. After 0.5 h at 0 °C, imine **2** (0.2 mmol) dissolved in anhydrous THF (1.0 mL) was added dropwise. Stirring was continued at 0 °C for 0.5 h. Then, the reaction was quenched with saturated NH_4_Cl (2.0 mL), followed by H_2_O (5.0 mL), and the mixture was brought to room temperature. The organic layer was taken, and the aqueous layer was extracted with CH_2_Cl_2_ (3 × 15 mL). The combined organic layers were dried with anhydrous Na_2_SO_4_, filtered, and the solvent was removed to give the crude product, **3**, which was purified via column chromatography using petroleum ether/ethyl acetate (4:1, *v*/*v*) as an eluent.

### 3.3. Procedure for the Synthesis of **4**, **5**, **6**

Then, compound **3a** (3.996 mmol) was dissolved in a 250 mL round-bottomed flask using MeOH (50 mL) as a solvent, and HCl solution (36%, 4 mL) was added dropwise to the reaction mixture, and then was stirred at room temperature for 12 h. Volatiles were removed under reduced pressure. The residue was dissolved in CH_2_Cl_2_ (50 mL), followed by Et_3_N to adjust the pH > 8. Then, H_2_O (10 mL) was added. The organic layer was taken, washed with H_2_O (3 × 100 mL), dried with anhydrous Na_2_SO_4_, filtered, and the solvent was removed to give the crude product, **4**, which was purified via column chromatography using petroleum ether/ethyl acetate (8:1, *v*/*v*) as an eluent (79% yield).

Then, compound **3b** (2.0 mmol) was dissolved in a 100 mL round-bottomed flask using MeOH (20 mL) as a solvent, and HCl solution (36%, 2 mL) was added dropwise to the reaction mixture, and then was stirred at room temperature for 12 h. Volatiles were removed under reduced pressure. The residue was dissolved in CH_2_Cl_2_ (20 mL), followed by Et_3_N to adjust the pH > 8. Then, H_2_O (10 mL) was added. The organic layer was taken, washed with H_2_O (3 × 100 mL), dried with anhydrous Na_2_SO_4_, filtered, and the solvent was removed to give the crude product, **5**, which was purified via column chromatography using petroleum ether/ethyl acetate (8:1, *v*/*v*) as an eluent.

Then, compound **3v** (1.269 mmol) was dissolved in a 100 mL round-bottomed flask using MeOH (20 mL) as a solvent, and HCl solution (36%, 1.3 mL) was added dropwise to the reaction mixture, and then was stirred at room temperature for 12 h. Volatiles were removed under reduced pressure. The residue was dissolved in CH_2_Cl_2_ (20 mL), followed by Et_3_N to adjust the pH > 8. Then, H_2_O (10 mL) was added. The organic layer was taken, washed with H_2_O (3 × 100 mL), dried with anhydrous Na_2_SO_4_, filtered, and the solvent was removed to give the crude product, **6**, which was purified via column chromatography using petroleum ether/ethyl acetate (8:1, *v*/*v*) as an eluent.

### 3.4. Large Scale Synthesis

Sclareolide (**1**) (6.02 mmol) and anhydrous THF (20 mL) were taken from an oven-dried reaction vial flushed with N_2_. The reaction vial was cooled to 0 °C, and LiHMDS (1 M in THF, 9.03 mmol) was added dropwise and stirred. After 0.5 h at 0 °C, imine **2a** (4.01 mmol) dissolved in anhydrous THF (10 mL) was added dropwise. Stirring was continued at 0 °C for 0.5 h. Then, the reaction was quenched with saturated NH_4_Cl (20 mL), followed by H_2_O (50 mL), and the mixture was brought to room temperature. The organic layer was taken, and the aqueous layer was extracted with CH_2_Cl_2_ (3 × 50 mL). The combined organic layers were dried with anhydrous Na_2_SO_4_, filtered, and the solvent was removed to give the crude product, **3a** (1.84 g, 99% yield), which was purified via column chromatography using petroleum ether/ethyl acetate (4:1, *v*/*v*) as an eluent.

### 3.5. In Vitro Antifungal Effects Studies

According to the screening method previously reported [46,47,48,49], the antifungal activity of **4** against two forest pathogenic fungi in vitro was tested. We dissolved compound **4** in DMSO to prepare a stock solution (10.0 g/L). We added the stock solution into PDA medium, and the concentration of the compound **4** in the medium was 50.0 mg/L. Pure DMSO without the target compound was used as the blank control, and sclareolide was used as the reference compound. We took a 6 mm diameter bacterial block from the edge of the fungus colony cultured via PDA and inoculated it on the three PDA media mentioned above. Each experiment was repeated three times. We calculated the relative inhibition rate *I* (%) of all test compounds using the following formula: *I* (%) = [(*C* − *T*)/(*C* − 6)] × 100%. In this equation, *I* was the inhibition rate, and *C* and *T* are the colony diameters of blank control (mm) and treatment (mm), respectively.

### 3.6. Product Identification

Compound **3a**: 87.4 mg, 95% yield, white solid, mp = 78–79 °C, ${\left[\alpha \right]}_{D}^{25}$ = −13.5 (c = 0.07, MeOH). ^1^H NMR (600 MHz, CDCl_3_): δ = 7.42–7.32 (m, 4H), 7.30–7.28 (m, 1H), 5.48 (br, 1H), 4.89–4.77 (m, 1H), 3.29 (dd, *J* = 4.20, 13.20 Hz, 1H), 2.03–1.97 (m, 1H), 1.86–1.77 (m, 1H), 1.72–1.43 (m, 6H), 1.38–1.36 (m, 4H), 1.33–1.31 (m, 1H), 1.20 (s, 9H), 1.11–1.06 (m, 1H), 1.01 (s, 3H), 0.87–0.77 (m, 7H). ^13^C{^1^H} NMR (150 MHz, CDCl_3_): δ = 178.4, 139.1, 128.8, 128.7, 128.0, 84.9, 59.0, 58.8, 56.6, 56.3, 47.6, 41.8, 40.6, 38.3, 37.9, 33.3, 33.2, 23.6, 22.7, 21.0, 20.5, 18.1, 16.3. HRMS (ESI) *m*/*z*: [M + H]^+^ calcd for C_27_H_42_NO_3_S^+^ 460.2880, found 460.2878.

Compound **3b**: 82.1 mg, 86% yield, white solid, mp = 81–83 °C, ${\left[\alpha \right]}_{D}^{25}$ = −29.9 (c = 0.07, MeOH). ^1^H NMR (600 MHz, CDCl_3_): δ = 7.41–7.35 (m, 2H), 7.06–6.99 (m, 2H), 5.53 (br, 1H), 4.84–4.75 (m, 1H), 3.28 (dd, *J* = 4.26, 13.26 Hz, 1H), 2.04–1.97 (m, 1H), 1.88–1.79 (m, 1H), 1.70–1.46 (m, 6H), 1.39–1.37 (m, 4H), 1.36–1.31 (m, 1H), 1.20 (s, 9H), 1.15–1.07 (m, 1H), 1.03 (s, 3H), 0.86–0.79 (m, 7H). ^13^C{^1^H} NMR (150 MHz, CDCl_3_): δ = 178.4, 163.1 (d, *J* = 246.0 Hz), 135.0 (d, *J* = 3.6 Hz), 130.6 (d, *J* = 7.7 Hz), 115.8 (d, *J* = 21.4 Hz), 85.1, 58.9, 58.5, 56.6, 56.3, 47.7, 41.8, 40.6, 38.3, 38.0, 33.3, 33.2, 23.6, 22.7, 21.0, 20.5, 18.1, 16.3. ^19^F NMR (565 MHz, CDCl_3_): δ = −114.0 (s). HRMS (ESI) *m*/*z*: [M + H]^+^ calcd for C_27_H_41_FNO_3_S^+^ 478.2786, found 478.2800.

Compound **3c**: 86.2 mg, 90% yield, white solid, mp = 75–76 °C, ${\left[\alpha \right]}_{D}^{25}$ = 6.7 (c = 0.06, MeOH). ^1^H NMR (600 MHz, CDCl_3_): δ = 7.35–7.30 (m, 1H), 7.22–7.16 (m, 1H), 7.15–7.11 (m, 1H), 7.02–6.95 (m, 1H), 5.50 (br, 1H), 4.88–4.77 (m, 1H), 3.28 (dd, *J* = 4.20, 13.32 Hz, 1H), 2.04–1.99 (m, 1H), 1.87–1.80 (m, 1H), 1.70–1.44 (m, 6H), 1.41–1.36 (m, 4H), 1.35–1.32 (m, 1H), 1.20 (s, 9H), 1.14–1.06 (m,1H), 1.02 (s, 3H), 0.85–0.76 (m, 7H). ^13^C{^1^H} NMR (150 MHz, CDCl_3_): δ = 178.2, 163.6 (d, *J* = 245.2 Hz), 141.7 (d, *J* = 5.5 Hz), 130.3 (d, *J* = 8.2 Hz), 124.5, 115.8 (d, *J* = 22.3 Hz), 115.2 (d, *J* = 20.9 Hz), 85.1, 58.9, 58.5, 56.7, 56.3, 47.7, 41.8, 40.7, 38.3, 38.0, 33.3, 33.2, 23.6, 22.7, 21.0, 20.5, 18.1, 16.2. ^19^F NMR (565 MHz, CDCl_3_): δ = −111.4 (s). HRMS (ESI) *m*/*z*: [M + H]^+^ calcd for C_27_H_41_FNO_3_S^+^ 478.2786, found 478.2797.

Compound **3d**: 89.0 mg, 93% yield, white solid, mp = 80–82 °C, ${\left[\alpha \right]}_{D}^{25}$ = −29.9 (c = 0.07, MeOH). ^1^H NMR (600 MHz, CDCl_3_): δ = 7.45–7.38 (m, 1H), 7.34–7.29 (m, 1H), 7.16–7.04 (m, 2H), 5.55 (d, *J* = 10.74 Hz, 1H), 5.25 (dd, *J* = 4.92, 10.80 Hz, 1H), 3.32 (dd, *J* = 4.92, 13.44 Hz, 1H), 2.09–1.98 (m, 1H), 1.87–1.78 (m, 1H), 1.75–1.67 (m, 1H), 1.65–1.52 (m, 4H),1.42 (s, 3H), 1.36–1.32 (m, 2H), 1.22 (s, 9H), 1.06–0.95 (m,4H), 0.85–0.77 (m, 7H), 0.72–0.63 (m, 1H). ^13^C{^1^H} NMR (150 MHz, CDCl_3_): δ = 178.9, 160.9 (d, *J* = 245.2 Hz), 130.0 (d, *J* = 8.7 Hz), 128.0 (d, *J* = 2.5 Hz), 127.0 (d, *J* = 12.4 Hz), 124.9 (d, *J* = 3.2 Hz), 116.2 (d, *J* = 23.7 Hz), 85.0, 59.3, 56.5, 56.2, 51.5, 46.2, 41.7, 39.4, 38.5, 37.5, 33.3, 33.2, 23.3, 22.7, 21.0, 20.5, 18.0, 16.1. ^19^F NMR (565 MHz, CDCl_3_): δ = −115.3 (s). HRMS (ESI) *m*/*z*: [M + H]^+^ calcd for C_27_H_41_FNO_3_S^+^ 478.2786, found 478.2803.

Compound **3e**: 90.0 mg, 91% yield, white solid, mp = 89–91 °C, ${\left[\alpha \right]}_{D}^{25}$ = −32.9 (c = 0.07, MeOH). ^1^H NMR (600 MHz, CDCl_3_): δ = 7.37–7.30 (m, 4H), 5.56 (br, 1H), 4.83–4.73 (m, 1H), 3.27 (dd, *J* = 4.32, 13.26 Hz, 1H), 2.03–1.98 (m, 1H), 1.87–1.79 (m, 1H), 1.70–1.60 (m, 3H), 1.56–1.45 (m, 2H), 1.40–1.37 (m, 4H), 1.36–1.29 (m, 1H), 1.19 (s, 9H), 1.15–1.06 (m, 1H), 1.04–0.98 (m, 4H), 0.87–0.78 (m, 7H). ^13^C{^1^H} NMR (150 MHz, CDCl_3_): δ = 178.3, 137.7, 134.0, 130.2, 129.0, 85.1, 58.9, 58.5, 56.6, 56.3, 47.6, 41.8, 40.6, 38.3, 38.0, 33.3, 33.2, 23.6, 22.7, 21.0, 20.5, 18.2, 16.3. HRMS (ESI) *m*/*z*: [M + H]^+^ calcd for C_27_H_41_ClNO_3_S^+^ 494.2490, found 494.2481.

Compound **3f**: 93.7 mg, 95% yield, white solid, mp = 80–81 °C, ${\left[\alpha \right]}_{D}^{25}$ = −13.0 (c = 0.07, MeOH). ^1^H NMR (600 MHz, CDCl_3_): δ = 7.41–7.35 (m, 1H), 7.30–7.28 (m, 3H), 5.50 (br, 1H), 4.85–4.74 (m, 1H), 3.27 (dd, *J* = 4.20, 13.26 Hz, 1H), 2.02–1.98 (m, 1H), 1.86–1.79 (m, 1H), 1.68–1.43 (m, 5H), 1.42–1.35 (m, 4H), 1.34–1.29 (m, 1H), 1.19 (s, 9H), 1.15–1.05 (m, 1H), 1.04–0.97 (m, 4H), 0.87–0.79 (m, 7H). ^13^C{^1^H} NMR (150 MHz, CDCl_3_): δ = 178.1, 141.2, 134.5, 130.1, 128.9, 128.3, 126.9, 85.1, 58.9, 58.5, 56.7, 56.3, 47.7, 41.8, 40.8, 38.3, 38.0, 33.3, 33.2, 23.6, 22.7, 21.0, 20.5, 18.1, 16.2. HRMS (ESI) *m*/*z*: [M + H]^+^ calcd for C_27_H_41_ClNO_3_S^+^ 494.2490, found 494.2482.

Compound **3g**: 64.3 mg, 65% yield, white solid, mp = 172–174 °C, ${\left[\alpha \right]}_{D}^{25}$ = −42.2 (c = 0.04, MeOH). ^1^H NMR (600 MHz, CDCl_3_): δ = 7.46–7.42 (m, 1H), 7.41–7.38 (m, 1H), 7.27–7.21 (m, 2H), 5.50–5.36 (m, 2H), 3.29 (dd, J = 5.16, 13.56 Hz, 1H), 2.12–2.05 (m, 1H), 1.89–1.81 (m, 1H), 1.76 (d, *J* = 13.62 Hz, 1H), 1.68–1.56 (m, 3H), 1.44–1.30 (m, 6H), 1.24 (s, 9H), 1.06–0.95 (m, 4H), 0.84–0.78 (m, 6H), 0.76–0.72 (m, 1H), 0.57–0.48 (m, 1H). ^13^C{^1^H} NMR (150 MHz, CDCl_3_): δ = 178.9, 136.9, 134.7, 130.8, 129.4, 127.6, 127.5, 84.9, 59.4, 56.5, 56.2, 55.7, 45.6, 41.7, 39.8, 38.6, 37.4, 33.2, 33.1, 23.2, 22.7, 21.0, 20.5, 17.9, 16.2. HRMS (ESI) *m*/*z*: [M + H]^+^ calcd for C_27_H_41_ClNO_3_S^+^ 494.2490, found 494.2482.

Compound **3h**: 95.0 mg, 88% yield, white solid, mp = 95–96 °C, ${\left[\alpha \right]}_{D}^{25}$ = −14.9 (c = 0.07, MeOH). ^1^H NMR (600 MHz, CDCl_3_): δ = 7.48–7.43 (m, 2H), 7.28–7.25 (m, 2H), 5.56 (br, 1H), 4.82–4.71 (m, 1H), 3.29 (dd, *J* = 4.32, 13.26 Hz, 1H), 2.03–1.97 (m, 1H), 1.87–1.79 (m, 1H), 1.70–1.59 (m, 3H), 1.54–1.44 (m, 2H), 1.38 (s, 3H), 1.34–1.29 (m, 1H), 1.19 (s, 9H), 1.14–1.06 (m, 1H), 1.05–0.98 (m, 4H), 0.91–0.77 (m, 8H). ^13^C{^1^H} NMR (150 MHz, CDCl_3_): δ = 178.3, 138.2, 132.0, 130.5, 122.3, 85.2, 58.9, 58.6, 56.6, 56.3, 47.6, 41.8, 40.6, 38.3, 38.0, 33.3, 33.2, 23.6, 22.7, 21.0, 20.5, 18.2, 16.3. HRMS (ESI) *m*/*z*: [M + H]^+^ calcd for C_27_H_41_BrNO_3_S^+^ 538.1985, found 538.1977.

Compound **3i**: 91.5 mg, 85% yield, white solid, mp = 76–78 °C, ${\left[\alpha \right]}_{D}^{25}$ = −30.3 (c = 0.07, MeOH). ^1^H NMR (600 MHz, CDCl_3_): δ = 7.54 (s, 1H), 7.46–7.41 (m, 1H), 7.38–7.32 (m, 1H), 7.25–7.19 (m, 1H), 5.50 (br, 1H), 4.84–4.74 (m, 1H), 3.28 (dd, *J* = 4.14, 13.26 Hz, 1H), 2.04–1.97 (m, 1H), 1.88–1.79 (m, 1H), 1.69–1.63 (m, 2H), 1.59–1.46 (m, 3H), 1.43–1.38 (m, 4H), 1.36–1.30 (m, 1H), 1.20 (s, 9H), 1.14–1.06 (m, 1H), 1.05–0.96 (m, 4H), 0.88–0.78 (m, 7H). ^13^C{^1^H} NMR (150 MHz, CDCl_3_): δ = 178.1, 141.5, 131.9, 131.3, 130.4, 127.4, 122.7, 85.1, 58.9, 58.3, 56.7, 56.3, 47.7, 41.9, 40.8, 38.3, 38.0, 33.3, 33.2, 23.6, 22.7, 21.0, 20.5, 18.2, 16.2. HRMS (ESI) *m*/*z*: [M + H]^+^ calcd for C_27_H_41_BrNO_3_S^+^ 538.1985, found 538.1980.

Compound **3j**: 87.5 mg, 81% yield, white solid, mp = 104–106 °C, ${\left[\alpha \right]}_{D}^{25}$ = −4.3 (c = 0.07, MeOH). ^1^H NMR (600 MHz, CDCl_3_): δ = 7.68–7.62 (m, 1H), 7.41–7.36 (m, 1H), 7.32–7.26 (m, 1H), 7.19–7.13 (m, 1H), 5.51–5.41 (m, 1H), 5.39–5.32 (m, 1H), 3.28 (dd, *J* = 5.22, 13.56 Hz, 1H), 2.13–2.07 (m, 1H), 1.87–1.82 (m, 1H), 1.77 (d, *J* = 13.56 Hz, 1H), 1.72–1.64 (m, 1H), 1.63–1.54 (m, 2H), 1.44–1.39 (m, 4H), 1.36–1.30 (m, 2H), 1.25 (s, 9H), 1.02–0.97 (m, 4H), 0.79 (s, 6H), 0.76–0.71 (m, 1H), 0.57–0.47 (m, 1H). ^13^C{^1^H} NMR (150 MHz, CDCl_3_): δ = 178.9, 138.2, 134.4, 129.6, 128.1, 127.9, 125.8, 84.8, 59.4, 58.3, 56.5, 56.2, 45.7, 41.7, 40.2, 38.6, 37.4, 33.3, 33.2, 23.2, 22.7, 21.0, 20.4, 17.8, 16.2. HRMS (ESI) *m*/*z*: [M + H]^+^ calcd for C_27_H_41_BrNO_3_S^+^ 538.1985, found 538.1983.

Compound **3k**: 81.6 mg, 83% yield, white solid, mp = 79–81 °C, ${\left[\alpha \right]}_{D}^{25}$ = −18.0 (c = 0.06, MeOH). ^1^H NMR (600 MHz, CDCl_3_): δ = 7.33–7.27 (m, 2H), 6.90–6.82 (m, 2H), 5.50 (br, 1H), 4.80–4.70 (m, 1H), 3.80 (s, 3H), 3.26 (dd, *J* = 4.38, 13.26 Hz, 1H), 2.03–1.97 (m, 1H), 1.86–1.77 (m, 1H), 1.72–1.61 (m, 3H), 1.54–1.45 (m, 2H), 1.41–1.36 (m, 4H), 1.33–1.30 (m, 1H), 1.20 (s, 9H), 1.14–0.98 (m, 5H), 0.88–0.79 (m, 7H). ^13^C{^1^H} NMR (150 MHz, CDCl_3_): δ = 178.5, 159.1, 131.2, 130.0, 114.1, 85.0, 58.8, 58.7, 56.5, 56.2, 55.1, 47.7, 41.8, 40.5, 38.3, 38.0, 33.3, 33.2, 23.7, 22.7, 21.0, 20.5, 18.2, 16.3. HRMS (ESI) *m*/*z*: [M + H]^+^ calcd for C_28_H_44_NO_4_S^+^ 490.2986, found 490.2981.

Compound **3l**: 83.3 mg, 85% yield, white solid, mp = 69–71 °C, ${\left[\alpha \right]}_{D}^{25}$ = −10.9 (c = 0.06, MeOH). ^1^H NMR (600 MHz, CDCl_3_): δ = 7.26–7.22 (m, 1H), 7.01–6.94 (m, 2H), 6.85–6.80 (m, 1H), 5.44 (br, 1H), 4.86–4.75 (m, 1H), 3.78 (s, 3H), 3.27 (dd, *J* = 4.26, 13.26 Hz, 1H), 2.03–1.98 (m, 1H), 1.86–1.78 (m, 1H), 1.73 (d, J = 13.20 Hz, 1H), 1.65–1.43 (m, 4H), 1.38 (s, 3H), 1.37–1.29 (m, 2H), 1.20 (s, 9H), 1.13–1.05 (m,2H), 1.01 (s, 3H), 0.88–0.85 (m, 1H), 0.84–0.78 (m, 6H). ^13^C{^1^H} NMR (150 MHz, CDCl_3_): δ = 178.4, 159.7, 140.6, 129.7, 121.0, 114.1, 113.8, 84.9, 58.9, 58.8, 56.6, 56.3, 55.2, 47.6, 41.9, 40.6, 38.3, 37.9, 33.3, 33.2, 23.6, 22.7, 21.0, 20.5, 18.2, 16.3. HRMS (ESI) *m*/*z:* [M + H]^+^ calcd for C_28_H_44_NO_4_S^+^ 490.2986, found 490.2983.

Compound **3m**: 62.5 mg, 64% yield, white solid, mp = 79–80 °C, ${\left[\alpha \right]}_{D}^{25}$ = −17.3 (c = 0.05, MeOH). ^1^H NMR (600 MHz, CDCl_3_): δ = 7.30–7.21 (m, 2H), 6.93–6.85 (m, 2H), 5.40–5.33 (m,1H), 5.31–5.22 (m, 1H), 3.87 (s, 3H), 3.26 (dd, *J* = 5.22, 13.56 Hz, 1H), 2.08–2.02 (m, 1H), 1.85–1.73 (m, 2H), 1.62–1.50 (m, 3H), 1.40 (s, 3H), 1.34–1.28 (m, 3H), 1.22 (s, 9H), 1.01–0.90 (m, 4H), 0.82–0.72 (m, 7H), 0.59–0.48 (m, 1H). ^13^C{^1^H} NMR (150 MHz, CDCl_3_): δ = 179.1, 156.7, 129.2, 128.0, 127.3, 120.8, 111.3, 84.6, 59.2, 56.2, 56.1, 55.4, 52.2, 45.8, 41.7, 39.1, 38.6, 37.4, 33.3, 33.2, 23.2, 22.8, 21.0, 20.5, 18.2, 16.1. HRMS (ESI) *m*/*z*: [M + H]^+^ calcd for C_28_H_44_NO_4_S^+^ 490.2986, found 490.2983.

Compound **3n**: 49.6 mg, 49% yield, white solid, mp = 76–77 °C, ${\left[\alpha \right]}_{D}^{25}$ = −9.8 (c = 0.03, MeOH). ^1^H NMR (600 MHz, CDCl_3_): δ = 7.31–7.21 (m, 2H), 6.91–6.84 (m, 1H), 5.48–5.40 (m, 1H), 5.23–5.14 (m, 1H), 4.19–4.12 (m, 1H), 4.07–4.00 (m, 1H), 3.29 (dd, *J* = 5.10, 13.50 Hz, 1H), 2.10–2.03 (m, 1H), 1.84–1.74 (m, 2H), 1.63–1.54 (m, 3H), 1.49 (t, *J* = 6.96 Hz, 3H), 1.41 (s, 3H), 1.34–1.28 (m, 3H), 1.23 (s, 9H), 1.05–0.95 (m, 4H), 0.84–0.73 (m, 7H), 0.63–0.53 (m, 1H). ^13^C{^1^H} NMR (150 MHz, CDCl_3_): δ = 179.2, 156.0, 129.1, 127.9, 127.0, 120.5, 111.8, 84.6, 63.6, 59.2, 56.2, 56.1, 52.0, 45.8, 41.7, 39.2, 38.6, 37.3, 33.3, 33.2, 23.2, 22.8, 21.0, 20.5, 18.1, 16.1, 14.9. HRMS (ESI) *m*/*z*: [M + H]^+^ calcd for C_29_H_46_NO_4_S^+^ 504.3142, found 504.3135.

Compound **3o**: 81.6 mg, 84% yield, white solid, mp = 105–107 °C, ${\left[\alpha \right]}_{D}^{25}$ = −22.6 (c = 0.07, MeOH). ^1^H NMR (600 MHz, CDCl_3_): δ = 7.68–7.61 (m, 2H), 7.56–7.49 (m, 1H), 5.60 (br, 1H), 4.90–4.78 (m, 1H), 3.30 (dd, *J* = 4.26, 13.38 Hz, 1H), 2.06–1.99 (m, 1H), 1.88–1.80 (m, 1H), 1.71–1.44 (m, 5H), 1.41–1.36 (m, 4H), 1.35–1.29 (m, 1H), 1.18 (s, 9H), 1.13–1.06 (m, 1H), 1.01 (s, 3H), 0.97–0.88 (m, 1H), 0.85–0.78 (m, 7H). ^13^C{^1^H} NMR (150 MHz, CDCl_3_): δ = 178.1, 144.6, 132.6, 129.6, 118.5, 112.1, 85.4, 59.0, 56.8, 56.4, 47.5, 41.7, 40.8, 38.3, 38.0, 33.3, 33.2, 23.5, 22.6, 21.0, 20.5, 18.1, 16.2. HRMS (ESI) *m*/*z*: [M + H]^+^ calcd for C_28_H_41_N_2_O_3_S^+^ 485.2832, found 485.2828.

Compound **3p**: 79.7 mg, 84% yield, white solid, mp = 78–80 °C, ${\left[\alpha \right]}_{D}^{25}$ = −28.5 (c = 0.06, MeOH). ^1^H NMR (600 MHz, CDCl_3_): δ = 7.27–7.22 (m, 2H), 7.16–7.11 (m, 2H), 5.48 (br, 1H), 4.83–4.73 (m, 1H), 3.27 (dd, *J* = 4.26, 13.26 Hz, 1H), 2.33 (s, 3H), 2.04–1.97 (m, 1H), 1.87–1.78 (m, 1H), 1.72–1.50 (m, 5H), 1.42–1.32 (m, 6H), 1.20 (s, 9H), 1.14–1.07 (m, 1H), 1.02 (s, 3H), 0.89–0.85 (m, 1H), 0.81 (s, 6H). ^13^C{^1^H} NMR (100 MHz, CDCl_3_): δ = 178.5, 137.6, 136.1, 129.5, 128.6, 84.9, 58.9, 58.8, 56.5, 56.2, 47.6, 41.8, 40.5, 38.3, 38.0, 33.3, 33.2, 23.6, 22.7, 21.1, 21.0, 20.6, 18.2, 16.3. HRMS (ESI) *m*/*z:* [M + H]^+^ calcd for C_28_H_44_NO_3_S^+^ 474.3036, found 474.3033.

Compound **3q**: 77.9 mg, 82% yield, white solid, mp = 71–73 °C, ${\left[\alpha \right]}_{D}^{25}$ = −18.7 (c = 0.06, MeOH). ^1^H NMR (600 MHz, CDCl_3_): δ = 7.24–7.14 (m, 3H), 7.13–7.07 (m, 1H), 5.41 (br, 1H), 4.84–4.74 (m, 1H), 3.27 (dd, *J* = 4.26, 13.20 Hz, 1H), 2.34 (s, 3H), 2.03–1.97 (m, 1H), 1.84–1.78 (m, 1H), 1.75–1.42 (m, 6H), 1.38–1.29 (m, 5H), 1.21 (s, 9H), 1.13–1.06 (m, 1H), 1.01 (s, 3H), 0.86–0.77 (m, 7H). ^13^C{^1^H} NMR (150 MHz, CDCl_3_): δ = 178.4, 139.1, 138.3, 129.3, 128.8, 128.6, 125.7, 84.8, 59.1, 58.8, 56.5, 56.3, 47.6, 41.9, 40.6, 38.4, 37.9, 33.3, 33.2, 23.6, 22.7, 21.0, 20.5, 18.2, 16.3. HRMS (ESI) *m*/*z:* [M + H]+ calcd for C_28_H_44_NO_3_S^+^ 474.3036, found 474.3033.

Compound **3r**: 81.2 mg, 79% yield, white solid, mp = 93–94 °C, ${\left[\alpha \right]}_{D}^{25}$ = −25.2 (c = 0.06, MeOH). ^1^H NMR (600 MHz, CDCl_3_): δ = 7.38–7.29 (m, 4H), 5.33 (br, 1H), 4.90–4.74 (m, 1H), 3.28 (dd, *J* = 4.08, 13.26 Hz, 1H), 2.03–1.97 (m, 1H), 1.87–1.82 (m, 1H), 1.78–1.71 (m, 1H), 1.68–1.44 (m, 5H), 1.41–1.34 (m, 5H), 1.31 (s, 9H), 1.21 (m, 9H), 1.15–1.09 (m, 1H), 1.02 (s, 3H), 0.90–0.87 (m, 1H), 0.83–0.81 (m, 6H). ^13^C{^1^H} NMR (150 MHz, CDCl_3_): δ = 178.2, 150.6, 128.3, 126.3, 125.6, 84.8, 58.7, 58.6, 56.5, 56.3, 47.8, 41.9, 40.6, 38.3, 37.9, 34.5, 33.3, 33.2, 31.3, 23.6, 22.7, 21.0, 20.6, 18.2, 16.3. HRMS (ESI) *m*/*z*: [M + H]^+^ calcd for C_31_H_50_NO_3_S^+^ 516.3506, found 516.3499.

Compound **3s**: 93.3 mg, 90% yield, white solid, mp = 70–72 °C, ${\left[\alpha \right]}_{D}^{25}$ = −20.0 (c = 0.08, MeOH). ^1^H NMR (600 MHz, CDCl_3_): δ = 7.33–7.25 (m, 2H), 6.87–6.81 (m, 1H), 5.49 (br, 1H), 4.80–4.70 (m, 1H), 3.92 (t, *J* = 6.54 Hz, 2H), 3.25 (dd, *J* = 4.38, 13.20 Hz, 1H), 2.02–1.97 (m, 1H), 1.86–1.76 (m, 3H), 1.70–1.44 (m, 6H), 1.41–1.36 (m, 4H), 1.33–1.30 (m, 1H), 1.20 (s, 9H), 1.13–1.08 (m, 1H), 1.05–0.97 (m, 6H), 0.86–0.83 (m, 1H), 0.81–0.78 (m, 6H). ^13^C{^1^H} NMR (150 MHz, CDCl_3_): δ = 178.5, 158.7, 130.9, 129.9, 114.6, 84.9, 69.4, 58.8, 58.6, 56.4, 56.2, 47.7, 41.8, 40.5, 38.3, 38.0, 33.3, 33.2, 23.6, 22.7, 22.6, 21.0, 20.5, 18.2, 16.3, 10.6. HRMS (ESI) *m*/*z*: [M + H]^+^ calcd for C_30_H_48_NO_4_S^+^ 518.3299, found 518.3295.

Compound **3t**: 100.5mg, 94% yield, white solid, mp = 95–97 °C, ${\left[\alpha \right]}_{D}^{25}$ = −36.4 (c = 0.08, MeOH). ^1^H NMR (600 MHz, CDCl_3_): δ = 7.65–7.57 (m, 4H), 7.51–7.46 (m, 2H), 7.46–7.39 (m, 2H), 7.36–7.31 (m, 1H), 5.58 (br, 1H), 4.98–4.81 (m, 1H), 3.33 (dd, *J* = 4.26, 13.26 Hz, 1H), 2.05–1.99 (m, 1H), 1.87–1.80 (m, 1H), 1.76 (d, *J* = 13.26 Hz, 1H), 1.71–1.46 (m, 5H), 1.44–1.41 (m, 4H), 1.35–1.32 (m, 1H), 1.24 (s, 9H), 1.14–1.10 (m, 1H), 1.05 (s, 3H), 0.90–0.87 (m, 1H), 0.83 (s, 6H). ^13^C{^1^H} NMR (150 MHz, CDCl_3_): δ = 178.4, 140.5, 140.4, 129.2, 128.8, 127.4, 127.3, 127.0, 85.0, 58.9, 58.8, 56.6, 56.3, 47.7, 41.9, 40.6, 38.3, 38.0, 33.3, 33.2, 23.6, 22.8, 21.0, 20.6, 18.2, 16.3. HRMS (ESI) *m*/*z*: [M + H]^+^ calcd for C_33_H_46_NO_3_S^+^ 536.3193, found 536.3184.

Compound **3u**: 83.8 mg, 82% yield, white solid, mp = 98–100 °C, ${\left[\alpha \right]}_{D}^{25}$ = 3.3 (c = 0.06, MeOH). ^1^H NMR (600 MHz, CDCl_3_): δ = 8.26 (d, *J* = 8.82 Hz, 1H), 7.88 (d, *J* = 7.98 Hz, 1H), 7.84 (d, *J* = 8.16 Hz, 1H), 7.62–7.57 (m, 1H), 7.56–7.48 (m, 2H), 7.46 (t, *J* = 7.80 Hz, 1H), 5.88–5.81 (m, 1H), 4.91 (br, 1H), 3.46 (dd, *J* = 4.56, 13.32 Hz, 1H), 2.14–2.08 (m, 1H), 2.00 (d, *J* = 13.38 Hz, 1H), 1.87–1.80 (m, 1H), 1.66–1.62 (m, 2H), 1.43 (s, 4H), 1.37–1.31 (m, 1H), 1.27 (s, 9H), 1.23–1.17 (m, 1H), 1.09–1.01 (m, 1H), 0.91–0.85 (m, 3H), 0.83–0.78 (m, 1H), 0.77–0.68 (m, 7H), 0.30–0.21 (m, 1H). ^13^C{^1^H} NMR (150 MHz, CDCl_3_): δ = 178.6, 134.4, 131.0, 129.1, 129.0, 126.6, 125.8, 125.0, 124.5, 123.0, 84.6, 59.5, 56.6, 56.3, 41.5, 38.7, 37.3, 33.2, 33.1, 23.3, 22.6, 20.9, 20.5, 17.8, 16.0. HRMS (ESI) *m*/*z:* [M + H]^+^ calcd for C_31_H_44_NO_3_S^+^ 510.3036, found 510.3031.

Compound **3v**: 64.3 mg, 71% yield, colorless oil, ${\left[\alpha \right]}_{D}^{25}$ = −7.3 (c = 0.04, MeOH). ^1^H NMR (600 MHz, CDCl_3_): δ = 5.77 (d, *J* = 10.44 Hz, 1H), 4.41–4.30 (m, 1H), 3.22 (d, *J* = 13.26 Hz, 1H), 2.19 (d, *J* = 13.80 Hz, 1H), 2.13–2.06 (m, 1H), 1.97–1.90 (m, 1H), 1.79–1.69 (m, 2H), 1.54–1.30 (m, 9H), 1.23 (s, 9H), 1.15–1.09 (m, 1H), 1.04 (s, 3H), 0.90 (s, 3H), 0.86 (s, 3H). ^13^C{^1^H} NMR (150 MHz, CDCl_3_): δ = 176.5, 126.1 (q, *J* = 282.2 Hz), 85.4, 58.5, 57.6, 56.3, 53.8 (q, *J* = 30.3 Hz), 44.7, 41.9, 39.2, 38.4, 38.0, 33.3, 33.2, 22.8, 22.2, 21.0, 20.6, 18.3, 15.9. ^19^F NMR (565 MHz, CDCl_3_): δ = −63.9 (s, 3F). HRMS (ESI) *m*/*z*: [M + H]^+^ calcd for C_22_H_37_F_3_NO_3_S^+^ 452.2441, found 452.2432.

Compound **4**: 56.3 mg, 79% yield, colorless oil, ${\left[\alpha \right]}_{D}^{25}$ = 9.8 (c = 0.10, MeOH). 1H NMR (400 MHz, CDCl3): δ = 7.44–7.22 (m, 5H), 4.40–4.32 (m, 1H), 3.36–3.26 (m, 1H), 2.48 (s, 2H), 2.05–1.94 (m, 1H), 1.89–1.77 (m, 1H), 1.75–1.52 (m, 4H), 1.43–1.31 (m, 6H), 1.14–0.94 (m, 5H), 0.93–0.74 (m, 7H). ^13^C{^1^H} NMR (100 MHz, CDCl3): δ = 178.7, 143.2, 128.8, 128.0, 127.4, 84.5, 58.9, 56.3, 56.2, 47.3, 41.8, 40.5, 38.3, 38.0, 33.3, 23.6, 21.0, 20.5, 18.1, 16.2. HRMS (ESI) *m*/*z*: [M + Na]^+^ calcd for C_23_H_33_NNaO_2_^+^ 378.2404, found 378.2413.

Compound **5**: 51.7 mg, 69% yield, colorless oil. ^1^H NMR (400 MHz, CDCl_3_): δ = 7.40–7.31 (m, 2H), 7.08–6.97 (m, 2H), 4.40–4.32 (m, 1H), 3.36–3.26 (m, 1H), 2.44 (s, 2H), 2.05–1.95 (m, 1H), 1.89–1.78 (m, 1H), 1.69–1.49 (m, 4H), 1.45–1.36 (m, 5H), 1.32–1.24 (m, 1H), 1.18–1.07 (m, 1H), 1.03–0.95 (m, 4H), 0.93–0.74 (m, 7H). ^13^C{^1^H} NMR (100 MHz, CDCl_3_): δ = 178.6, 163.2 (d, *J* = 244.8 Hz), 138.9, 129.8 (d, *J* = 7.87 Hz), 115.7 (d, *J* = 20.9 Hz), 84.6, 59.0, 56.3, 55.7, 47.4, 41.8, 40.6, 38.3, 38.0, 33.3, 33.2, 23.6, 21.0, 20.5, 18.1, 16.2. ^19^F NMR (376 MHz, CDCl_3_): δ = −114.0.

Compound **6**: 45.7 mg, 66% yield, colorless oil. ^1^H NMR (400 MHz, CDCl_3_): δ = 3.81–3.70 (m, 1H), 3.12–3.05 (m, 1H), 2.28–2.15 (m, 3H), 2.11–2.03 (m, 1H), 1.98–1.87 (m, 1H), 1.78–1.60 (m, 2H), 1.55–1.22 (m, 9H), 1.17–1.08 (m, 1H), 1.02 (s, 3H), 0.95–0.78 (m, 6H). ^13^C{^1^H} NMR (100 MHz, CDCl_3_): δ = 176.0, 130.5 (q, *J* = 283.5 Hz), 84.7, 58.2, 56.3, 54.7 (q, *J* = 28.6), 43.3, 41.8, 39.3, 38.4, 38.0, 33.4, 22.9, 21.0, 20.6, 18.2, 16.0. ^19^F NMR (376 MHz, CDCl_3_): δ = −68.7.

## 4. Conclusions

In conclusion, we have developed an asymmetric Mannich reaction of chiral sulfinyl imines with sclareolide as a new nucleophilic reagent. This mild and effective asymmetric system can be used with a wide range of substrates and has a high-functional-group tolerance, resulting in moderate-to-high yields and high diastereoselectivities of the aminoalkyl sclareolide derivatives. Furthermore, aminoalkyl sclareolide derivatives **4**–**6** have been proven to effectively inhibit two kinds of forest pathogenic fungi: *F. Oxysporum* and *L. Theobromae*.

## Figures and Tables

**Table 1:** Optimization of the reaction conditions ^a^.

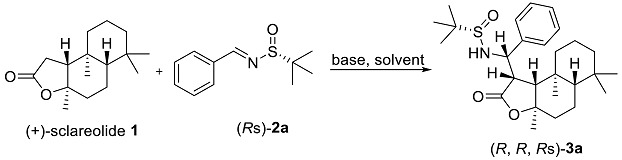							
Entry	Base	1 (Equiv)	Solvent	Time (h)	T (°C)	Yield (%) ^b^	Dr ^c^
1	LiHMDS	1.2	THF	4	0	69	92:8:0:0
2	MeONa	1.2	THF	4	0	trace	-
3	LDA	1.2	THF	4	0	48	93:7:0:0
4	*n*-BuLi	1.2	THF	4	0	trace	-
5	LiHMDS	1.2	THF	4	rt	trace	-
6	LiHMDS	1.2	THF	4	−78	31	97:3:0:0
7	LiHMDS	1.2	THF	1	0	88	96:4:0:0
8	LiHMDS	1.2	THF	12	0	90	95:5:0:0
9	LiHMDS	1.2	THF	0.5	0	95	94:6:0:0
10	LiHMDS	1.2	DCM	0.5	0	20	94:6:0:0
11	LiHMDS	1.2	MeCN	0.5	0	nd	
12	LiHMDS	1.2	1,4-dioxane	0.5	0	46	91:8:1:0
13	LiHMDS	1.5	THF	0.5	0	95	98:2:0:0

^a^ Reaction conditions: Sclareolide **1** (0.3 mmol) and anhydrous THF (2 mL) were added into an oven-dried flask flushed using N_2_. After cooling to 0 °C, LiHMDS (1 M in THF, 0.45 mL) was added dropwise and stirred. Then, aldimine **2a** (0.2 mmol) dissolved in anhydrous THF (1 mL) was added dropwise, and stirring was continued at 0 °C for 0.5 h. ^b^ Isolated yield. ^c^ Determined via ^1^H NMR.

**Table 2:** In vitro antifungal activities of the target compound, **4**, **5** and **6** at 50.0 mg/L.

Compound	Structure	Inhibition Rate (%) ^a^	
		*F. oxysporum*	*L. theobromae*
**1**	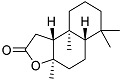	20 ± 4.3	53 ± 1.1
**4**	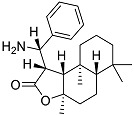	48 ± 2.5	67 ± 3.4
**5**	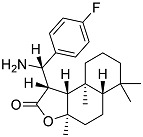	53 ± 3.2	61 ± 16.4
**6**	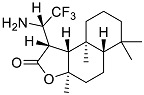	26 ± 1.0	52 ± 4.2

^a^ Values are the mean ± standard deviation (SD) of three replicates.

## Data Availability

Not applicable.

## Supplementary Materials

The following are available online at https://www.mdpi.com/article/10.3390/molecules28104067/s1, NMR spectra. Figure S1: Single crystal x-ray analysis of **3d**; Figure S2: Mycelia growth of *F. Oxysporum*; Figure S3: Mycelia growth of *L. Theobromae*.

## Sample Availability

Samples of the compounds **3**–**6** are available from the authors.
