# Supplementary material for: Synthesis of Aminoalkyl Sclareolide Derivatives and Antifungal Activity Studies
Source: Molecules. 2023 May 12;28(10):4067. doi: 10.3390/molecules28104067 (PMC10223150; doi:10.3390/molecules28104067)

# Supporting Information

## Table of Contents

**1. Single crystal x-ray analysis of 3d………………………………….S2**

**2. Mycelia growth of *F. oxysporum* and *L. theobromae* …………..…… S2**

**3. $^{1}$H, $^{13}$C and $^{19}$F NMR spectra for product 3, 4, 5 and 6..……....…. S5**

## 1. Single crystal x-ray analysis of 3d

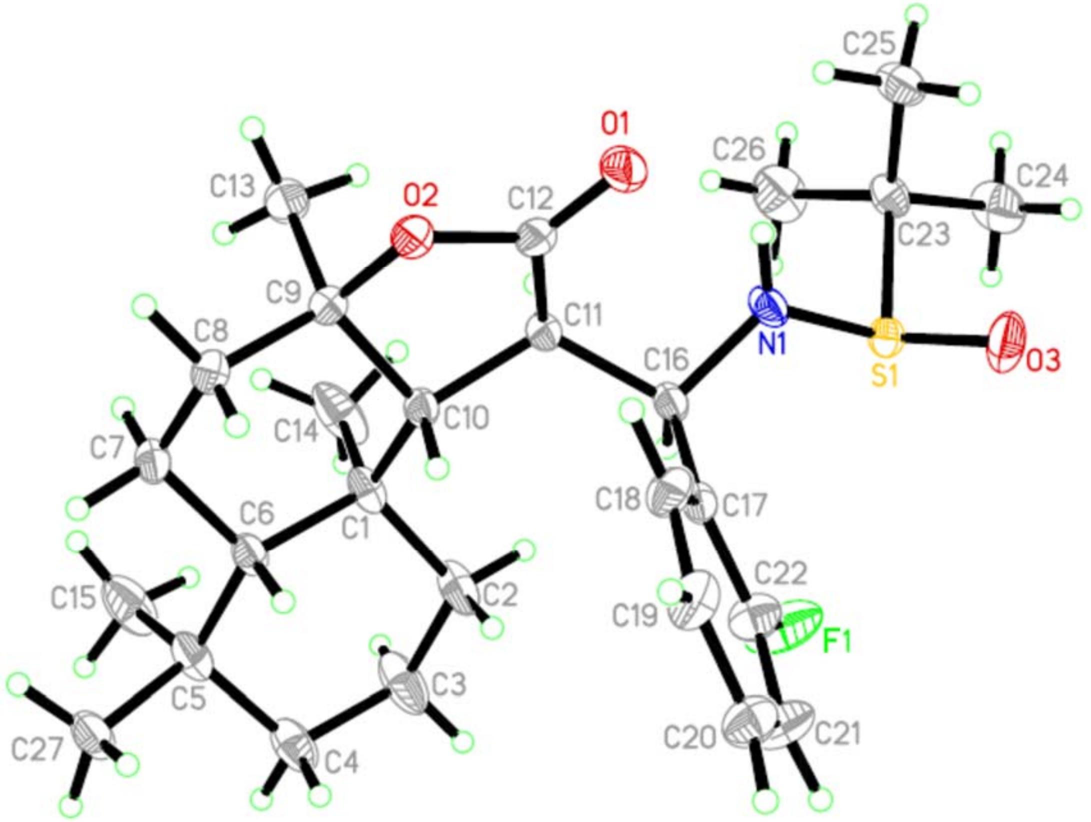


**Figure S1.** Single crystal x-ray analysis of **3d** (CCDC 2130078, the ellipsoids are drawn at a 30% probability level).

Suitable crystals of compound **3d** were obtained by slowly evaporating a mixture of petroleum ether and ethyl acetate solution at ambient temperature.

## 2. Mycelia growth of *F. oxysporum* and *L. theobroma*

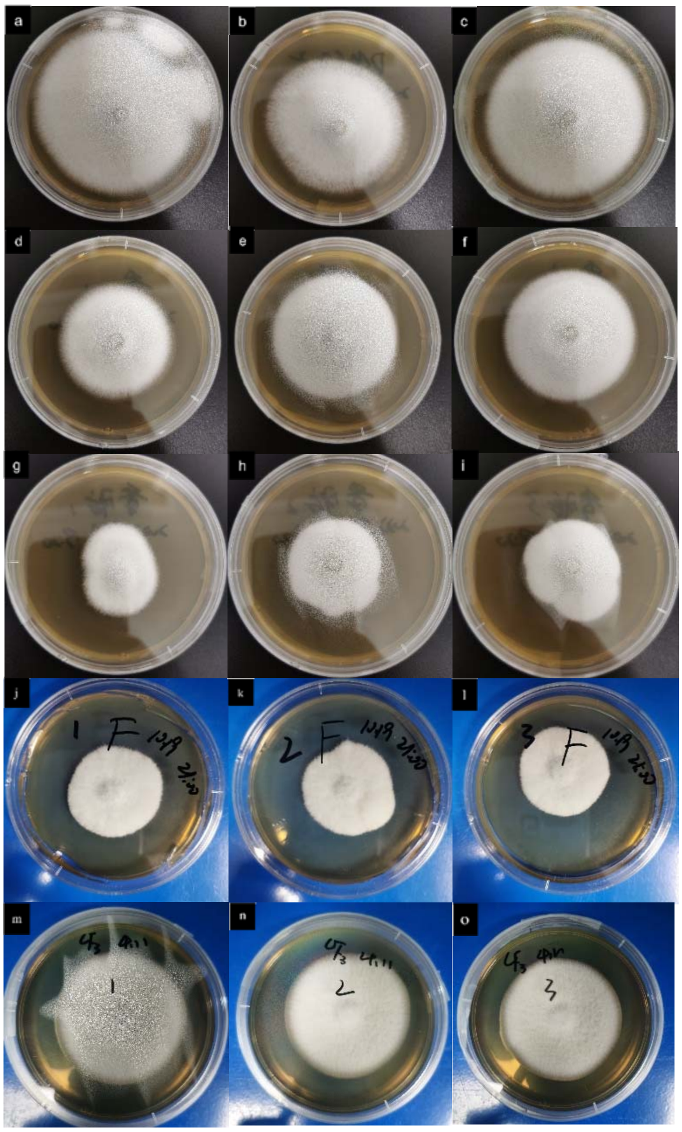


**Figure S2**. Mycelia growth of *F. oxysporum* after treating with DMSO: a, b, c; Sclareolide: d, e, f; compound **4**: g, h, i; compound **5**: j, k, l; compound **6**: m, n, o (50 mg/L).

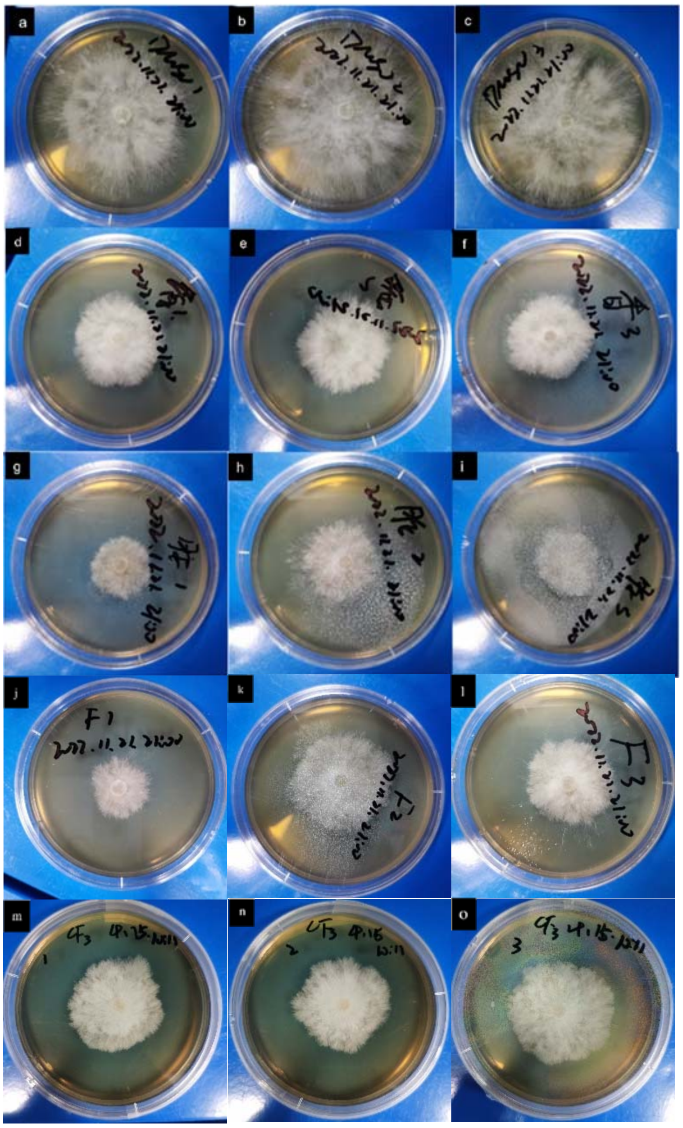


**Figure S3**. Mycelia growth of *L. theobromae* after treating with DMSO: a, b, c; Sclareolide: d, e, f; compound **4**: g, h, i; compound **5**: j, k, l; compound **6**: m, n, o (50 mg/L).

.

## 3. $^{1}$H, $^{13}$C and $^{19}$F spectra for product 3, 4, 5 and 6

$^{1}$H NMR (600 MHz, $CDCl_3$) of **3a**:

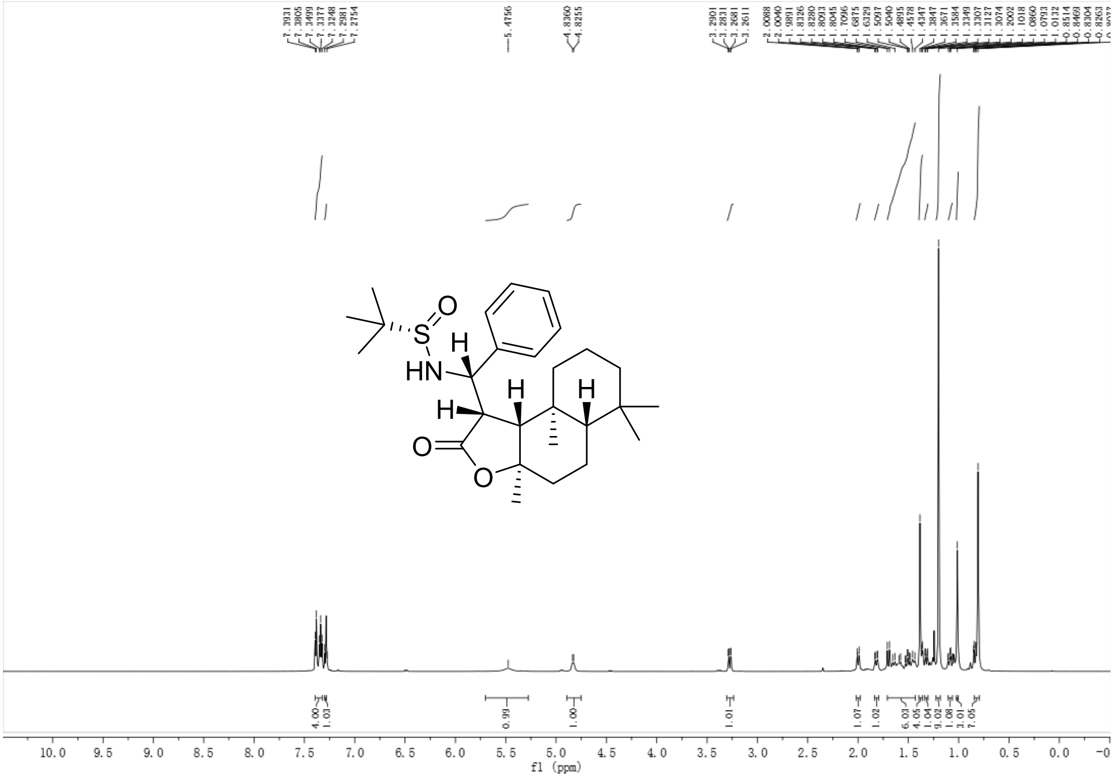


$^{13}$C{$^{1}$H} NMR (150 MHz, $CDCl_3$) of **3a**:

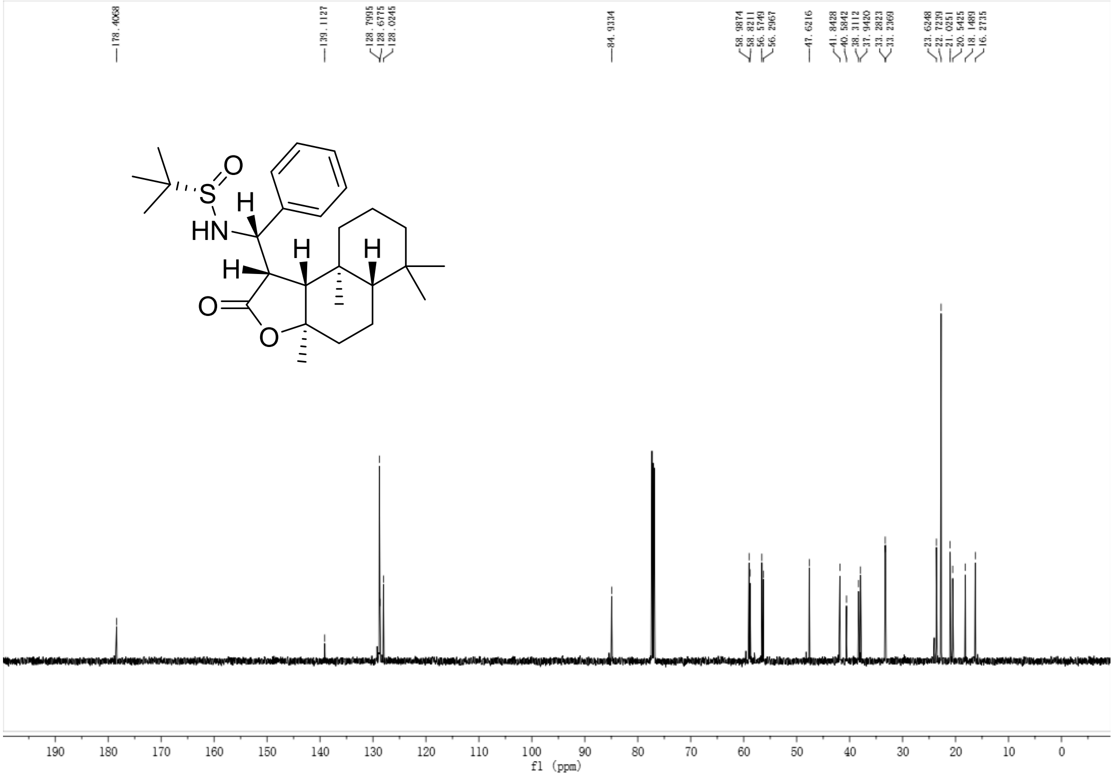

$^{1}H$ NMR (600 MHz, $CDCl_3$) of **3b**:

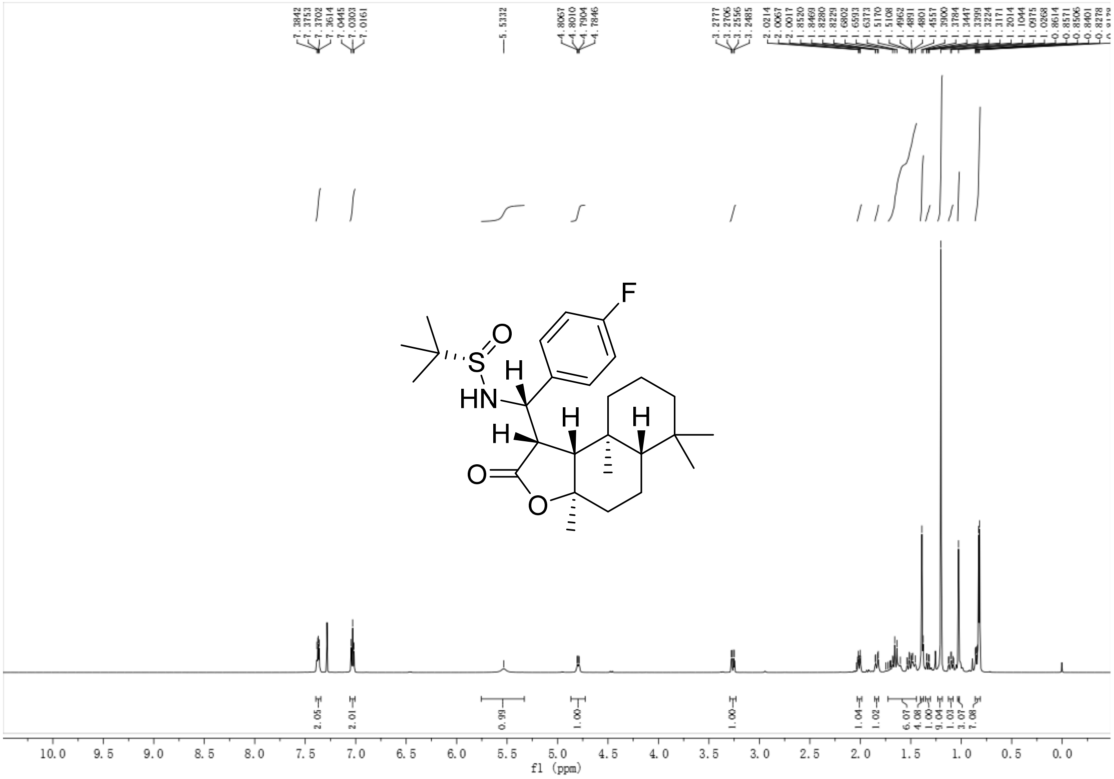

$^{13}C\{^{1}H\}$ NMR (150 MHz, $CDCl_3$) of **3b**:

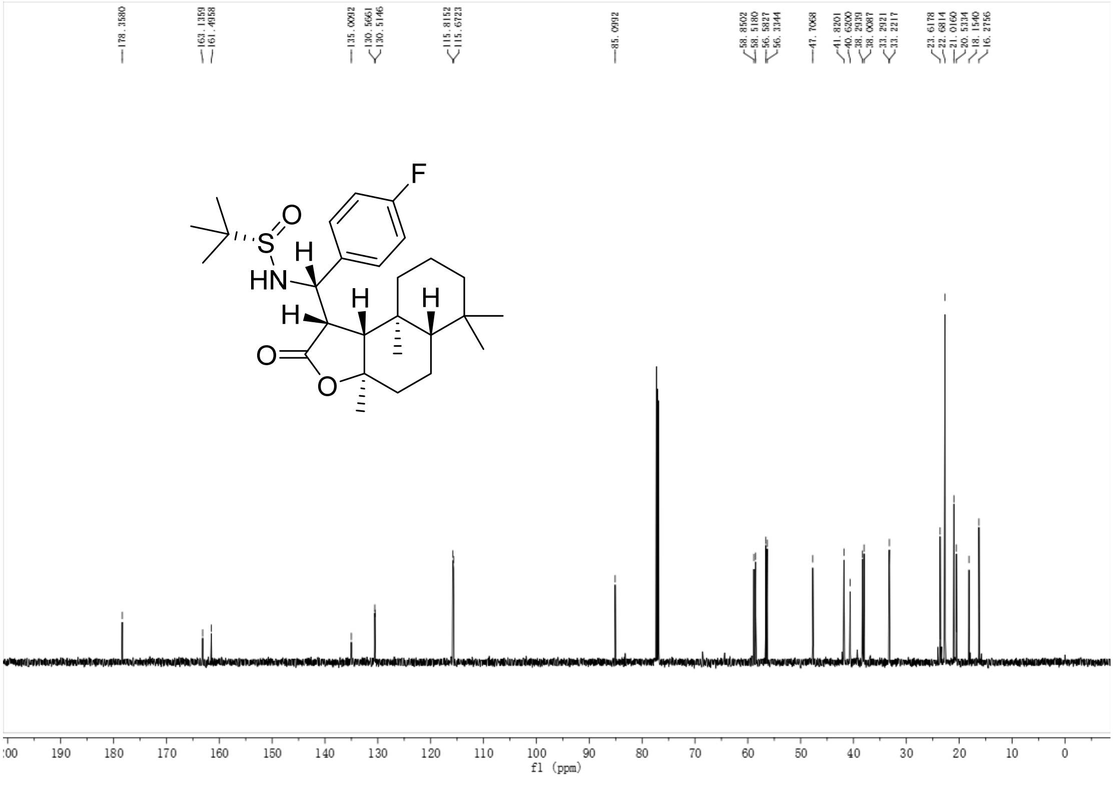

$^{19}F$ NMR (565 MHz, $CDCl_3$) of **3b**:

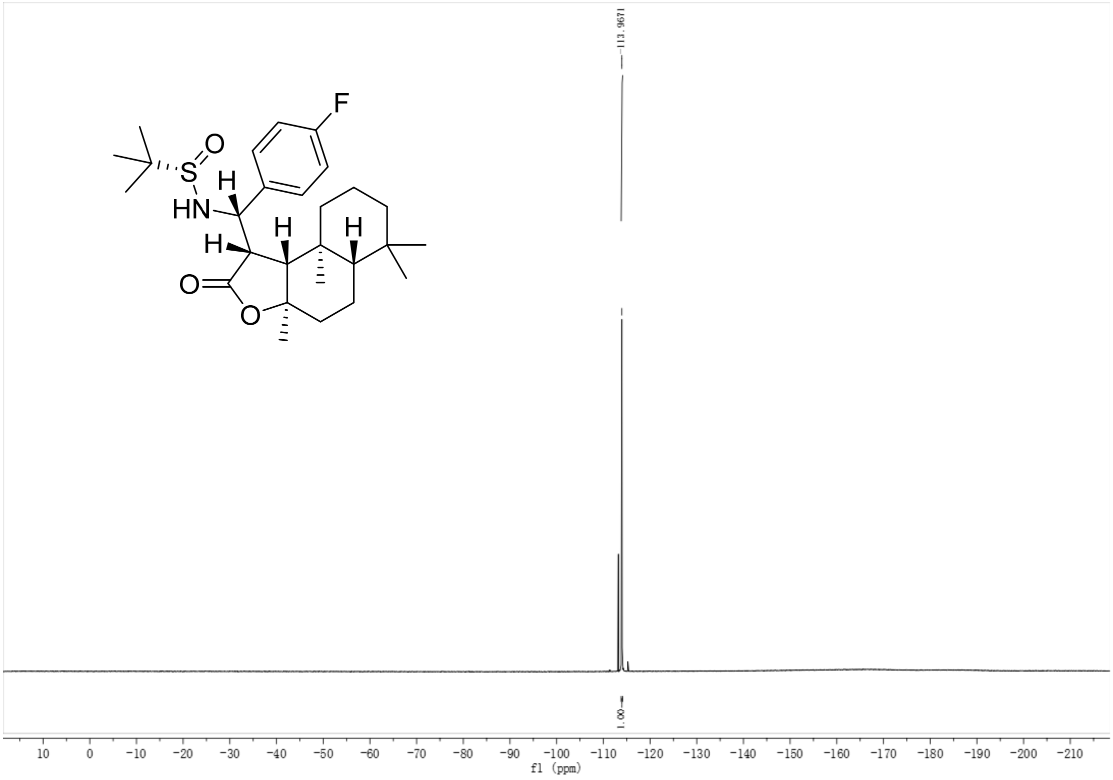


$^{1}H$ NMR (600 MHz, $CDCl_3$) of **3c**:

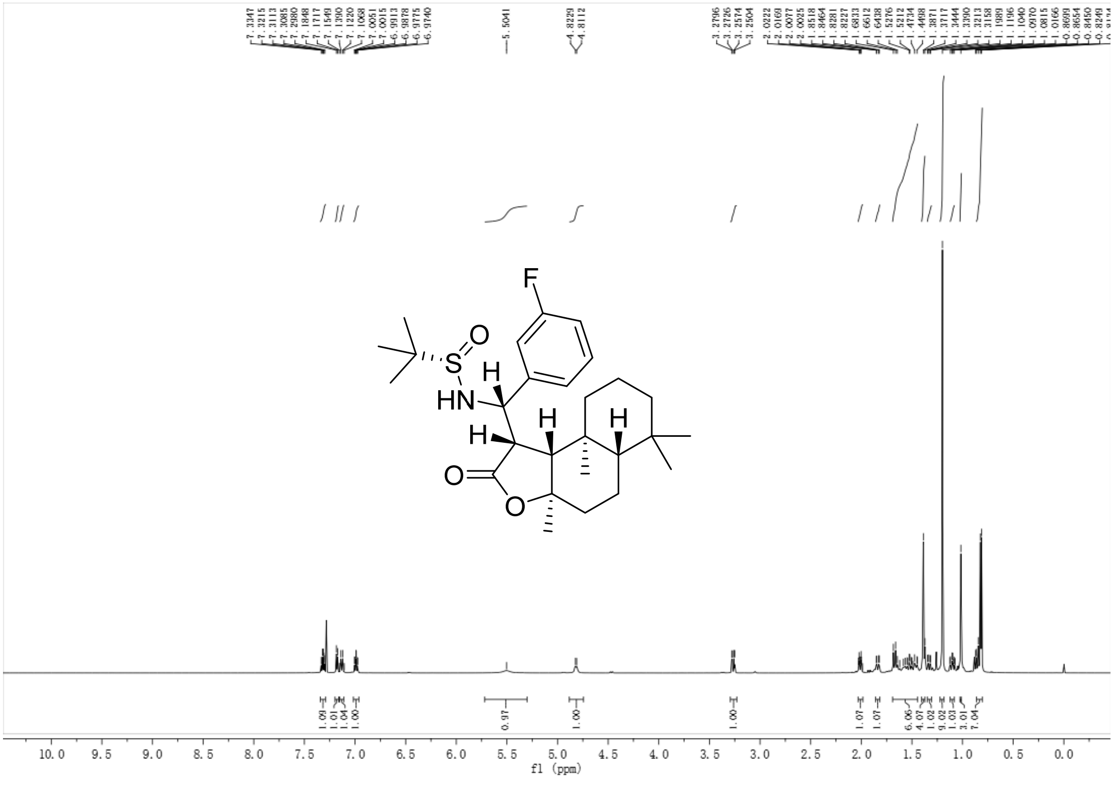

$^{13}C\{^{1}H\}$ NMR (150 MHz, $CDCl_3$) of **3c**:

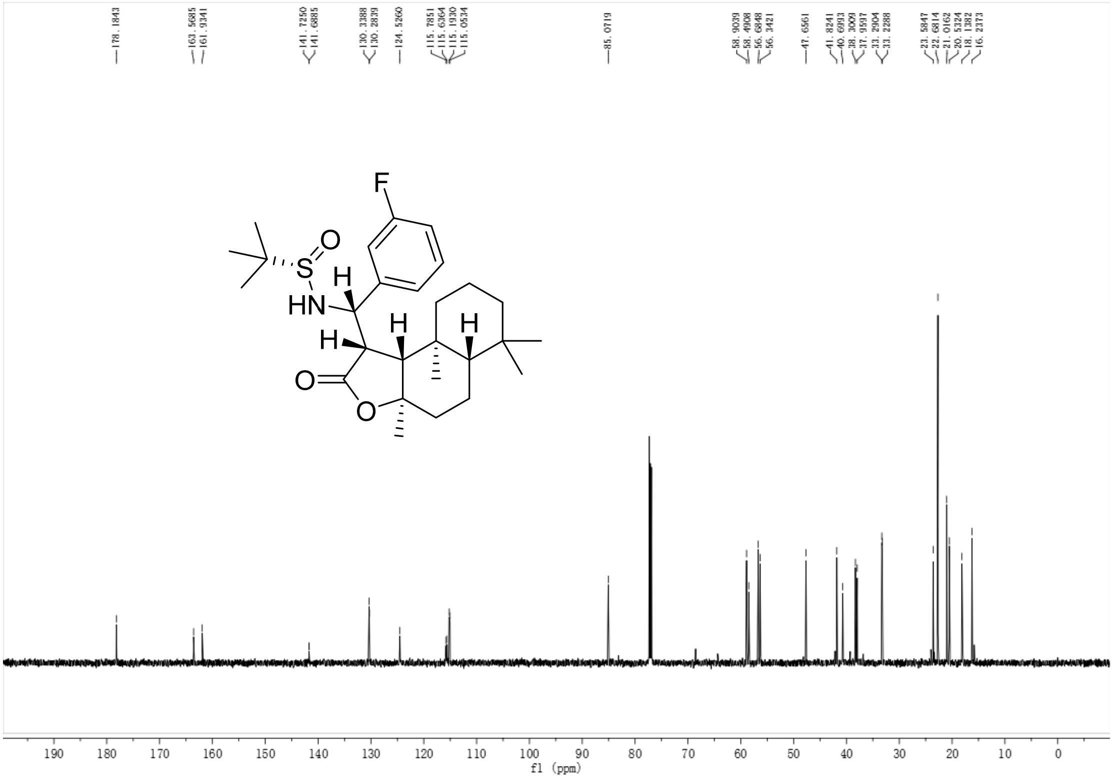


$^{19}F$ NMR (565 MHz, $CDCl_3$) of **3c**:

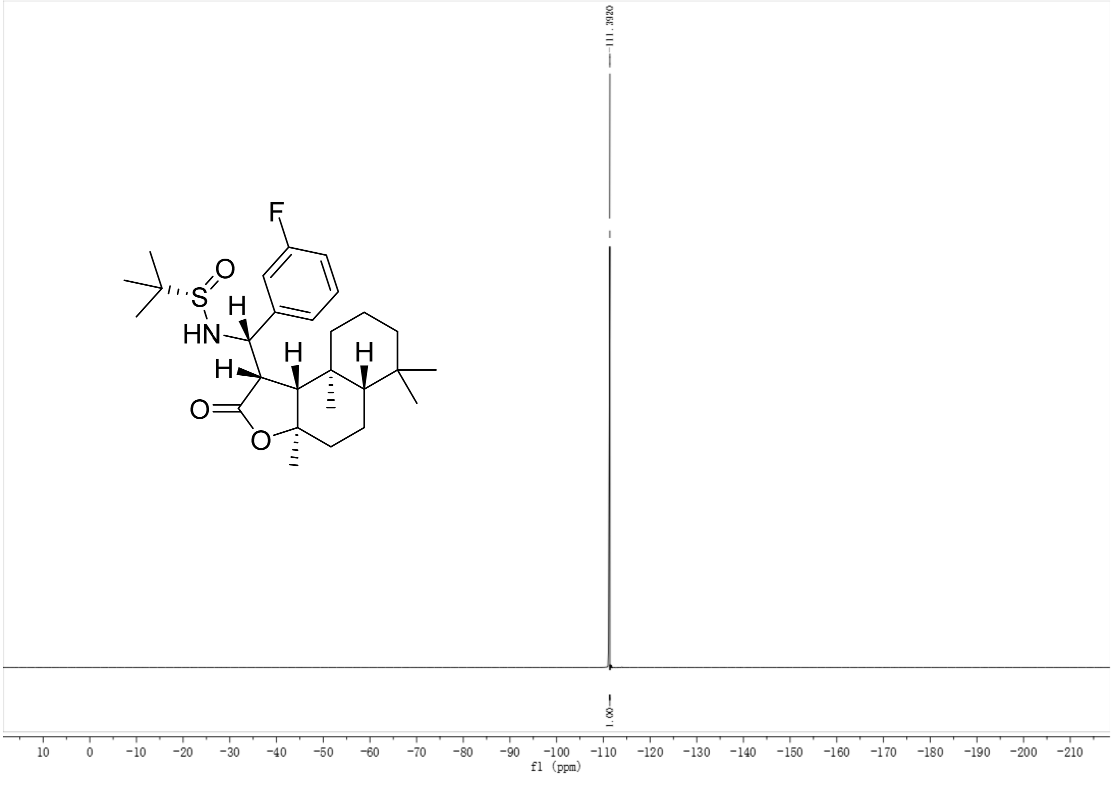

$^{1}H$ NMR (600 MHz, $CDCl_3$) of **3d**:

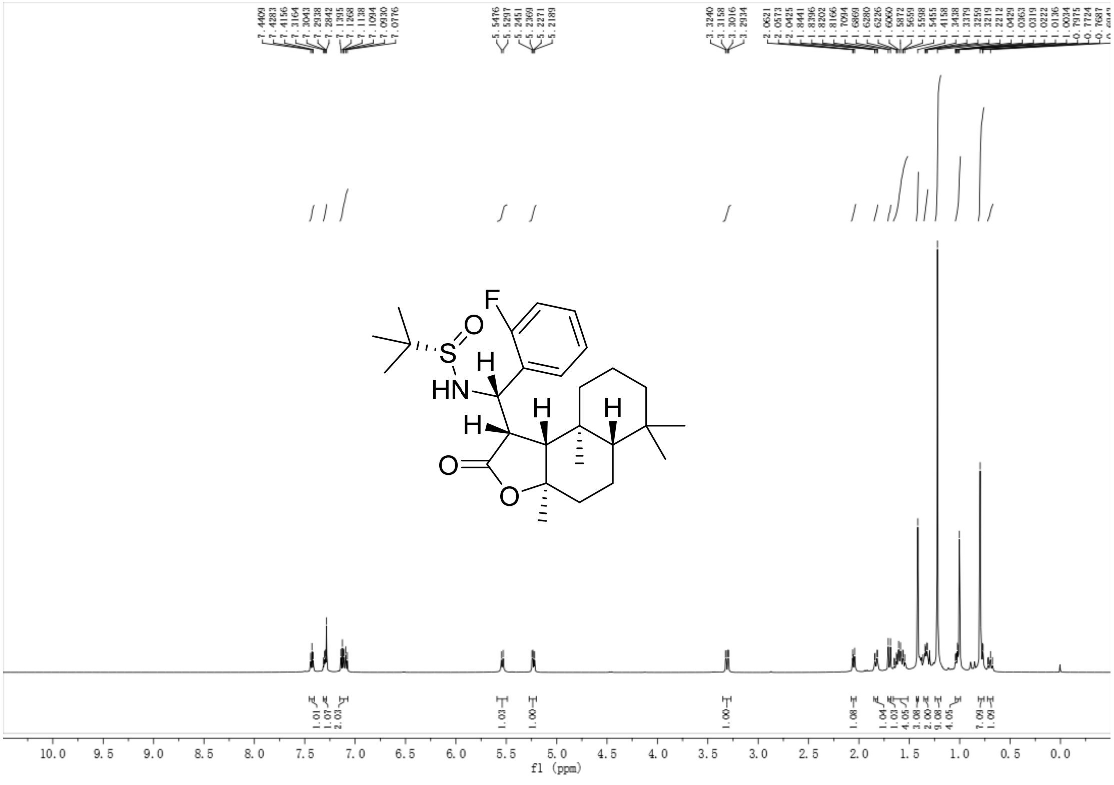

$^{13}C\{^{1}H\}$ NMR (150 MHz, $CDCl_3$) of **3d**:

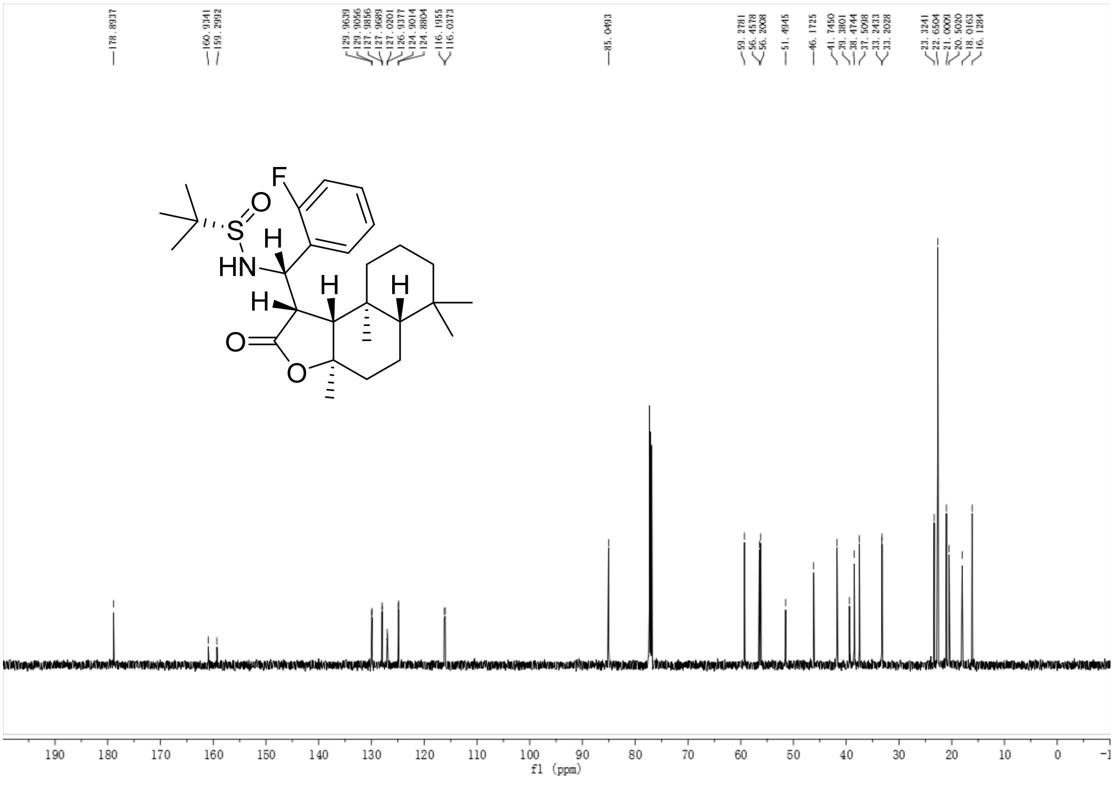

$^{19}$F NMR (565 MHz, CDCl$_3$) of **3d**:

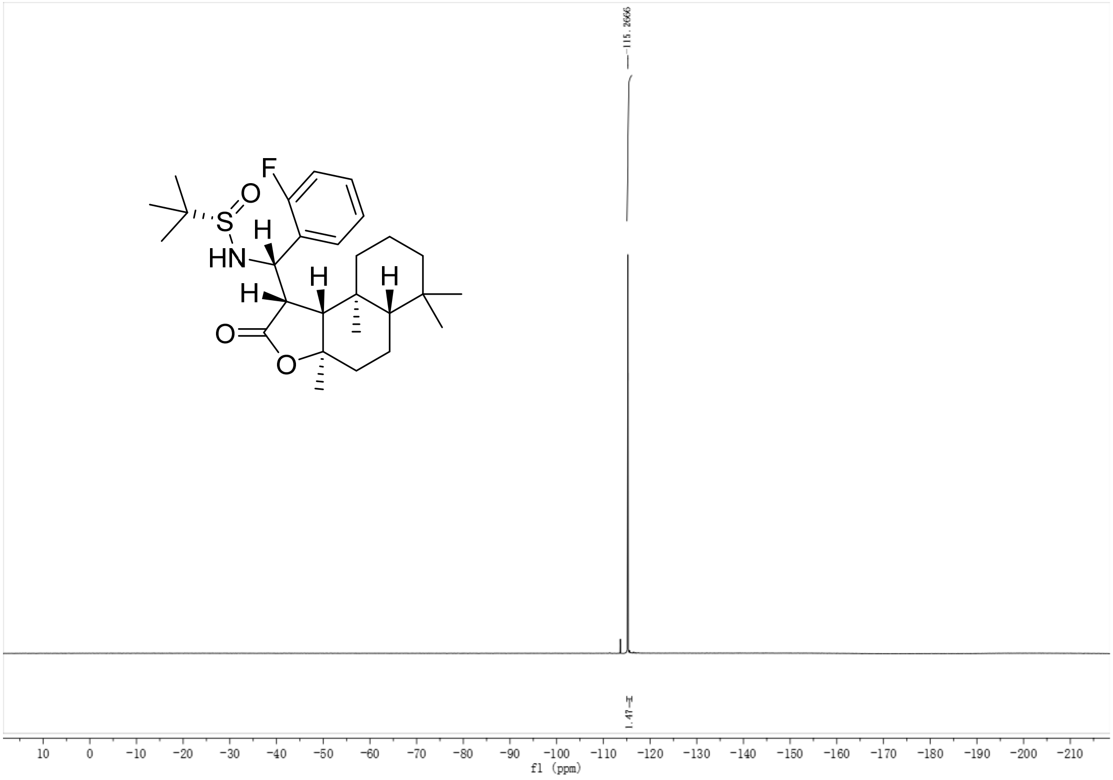

$^{1}$H NMR (600 MHz, CDCl$_3$) of **3e**:

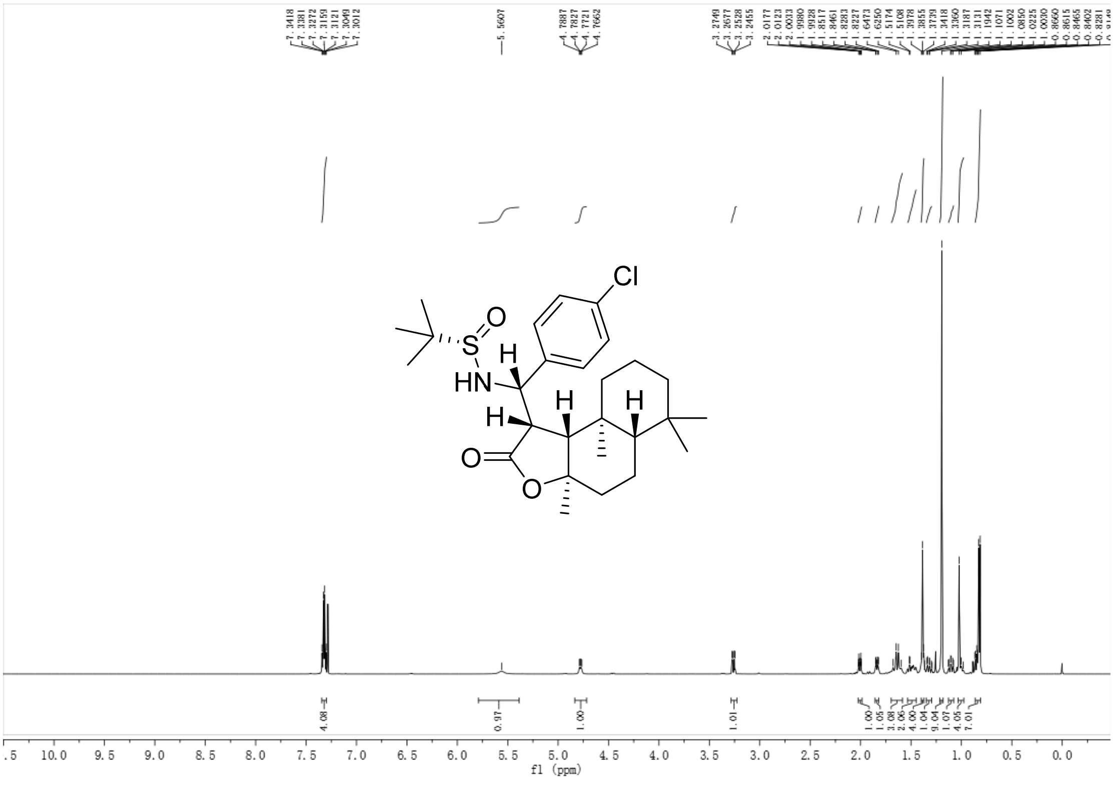

$^{13}C\{^{1}H\}$ NMR (150 MHz, $CDCl_3$) of **3e**:

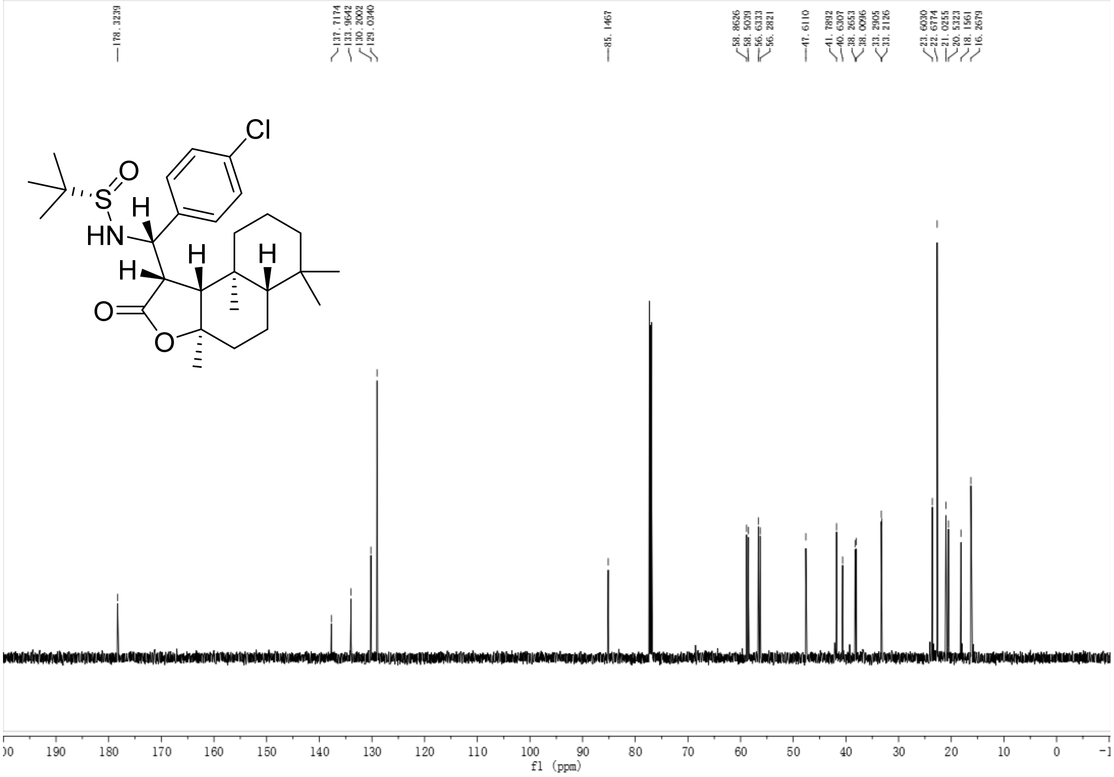


$^{1}H$ NMR (600 MHz, $CDCl_3$) of **3f**:

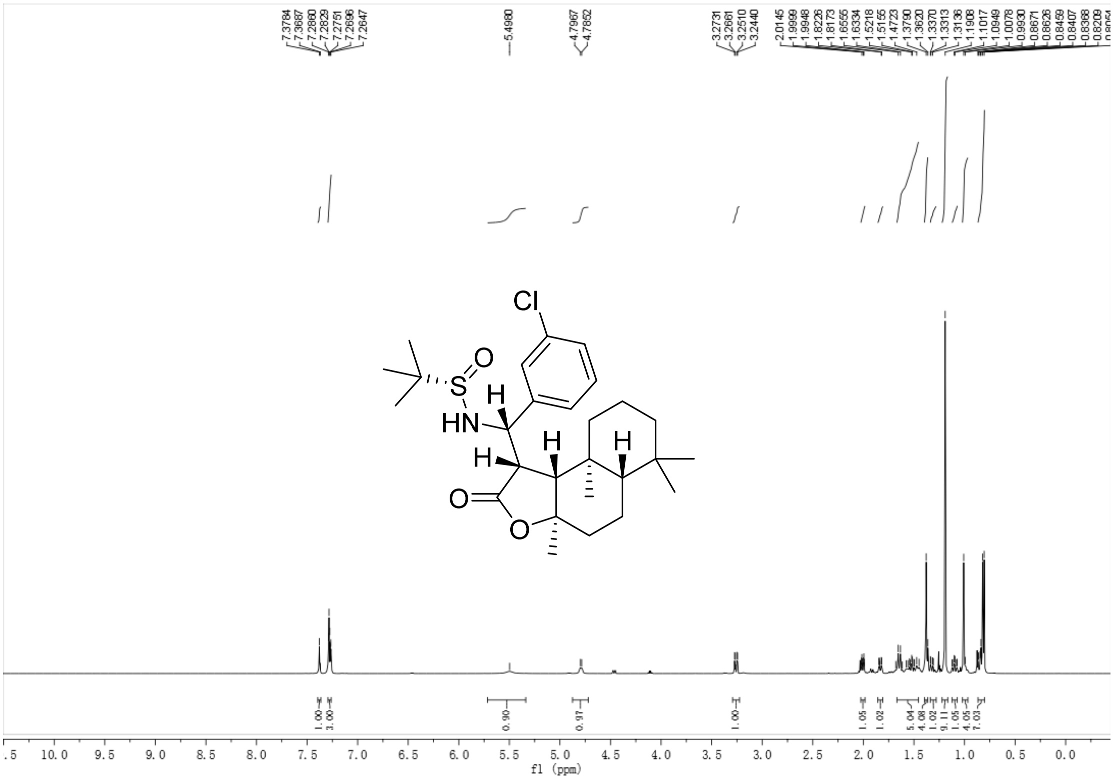

$^{13}C\{^1H\}$ NMR (150 MHz, $CDCl_3$) of **3f**:

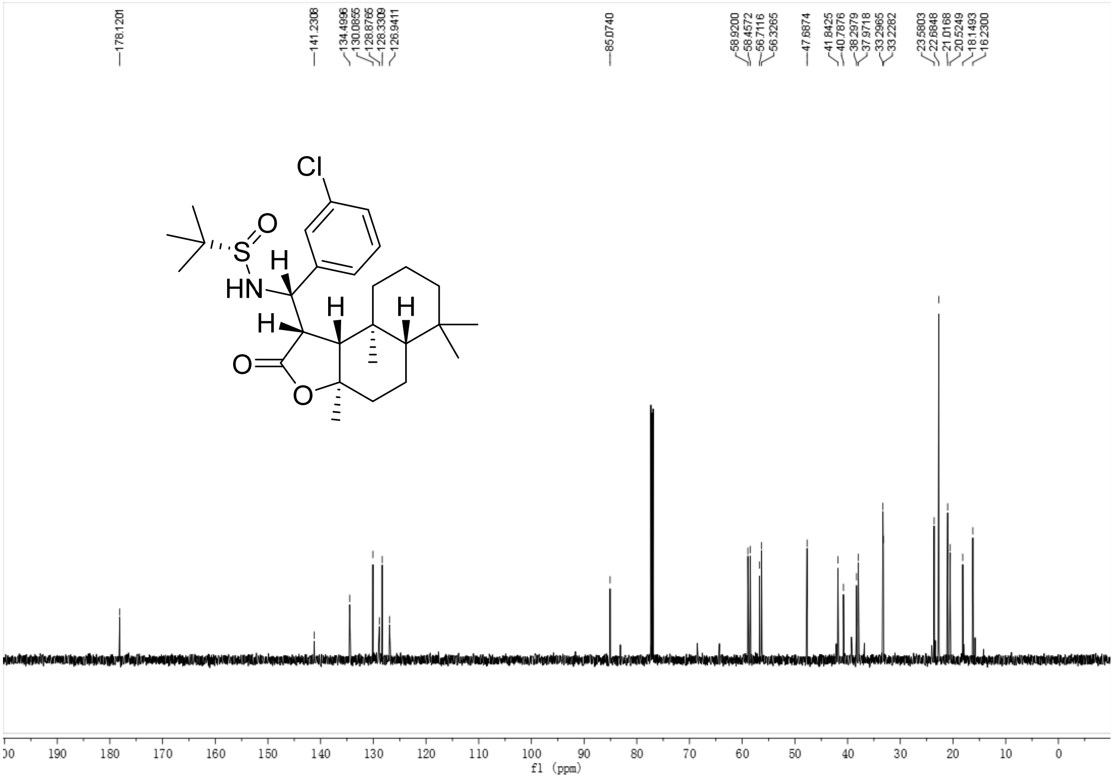

$^1H$ NMR (600 MHz, $CDCl_3$) of **3g**:

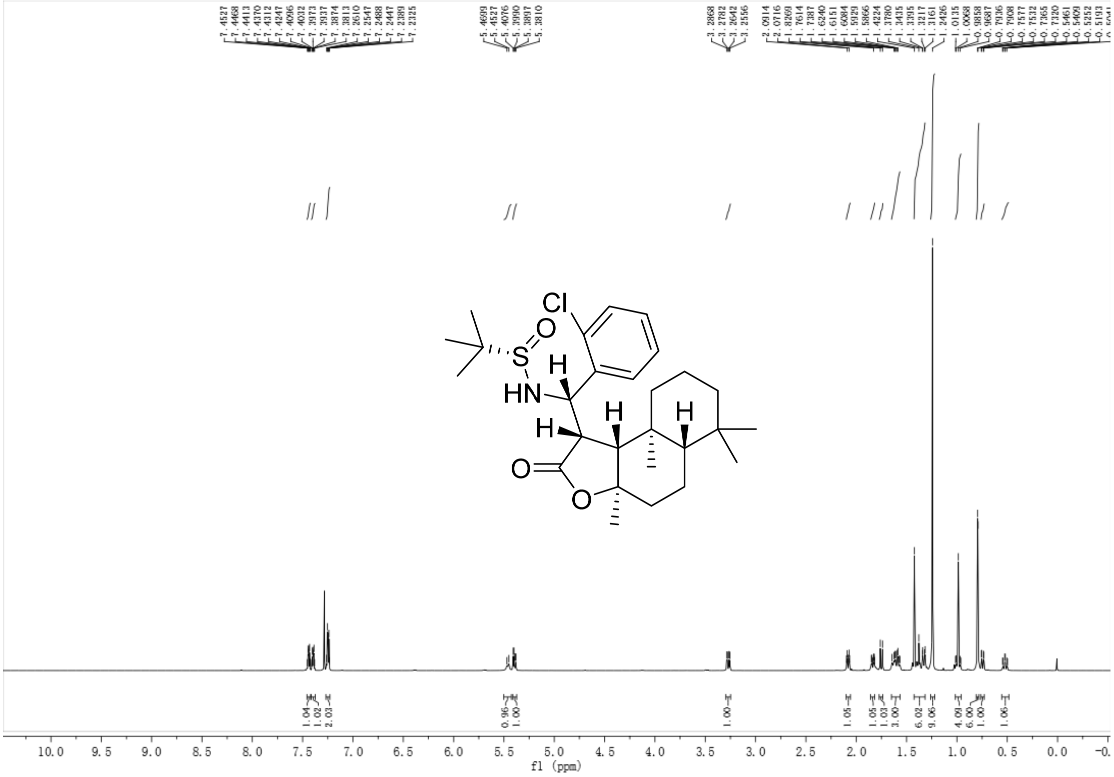

$^{13}C\{^{1}H\}$ NMR (150 MHz, $CDCl_3$) of **3g**:

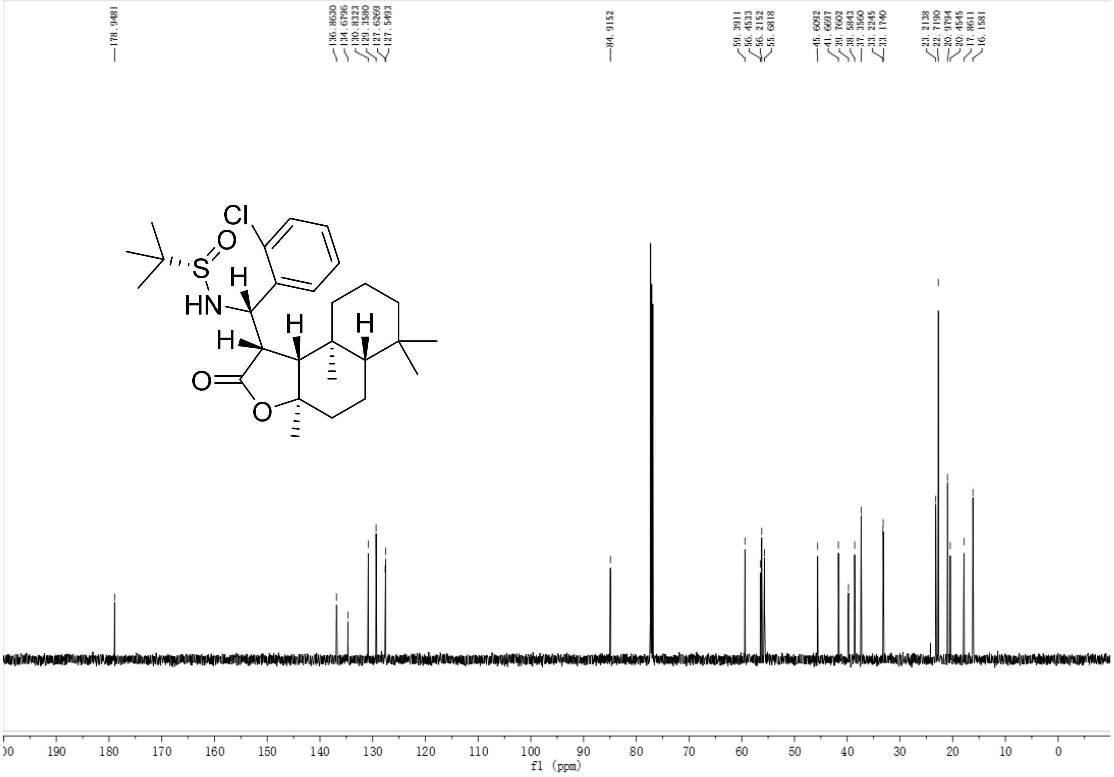

$^{1}H$ NMR (600 MHz, $CDCl_3$) of **3h**:

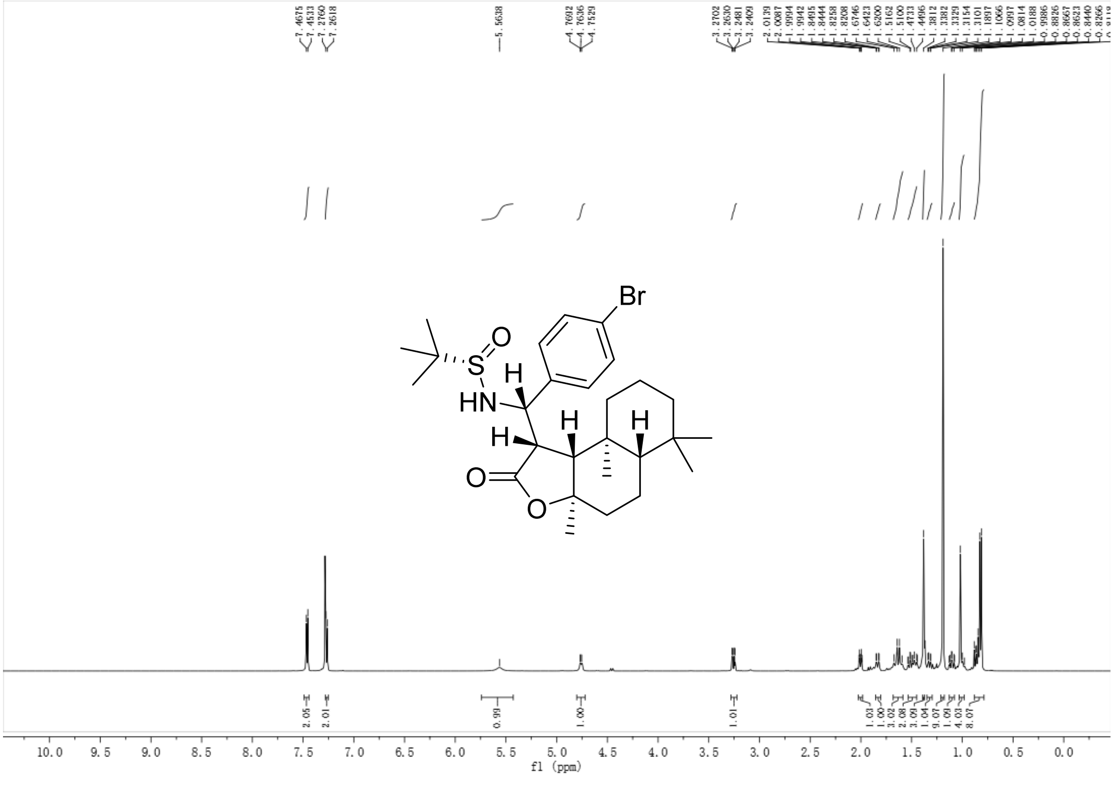

$^{13}C\{^1H\}$ NMR (150 MHz, $CDCl_3$) of **3h**:

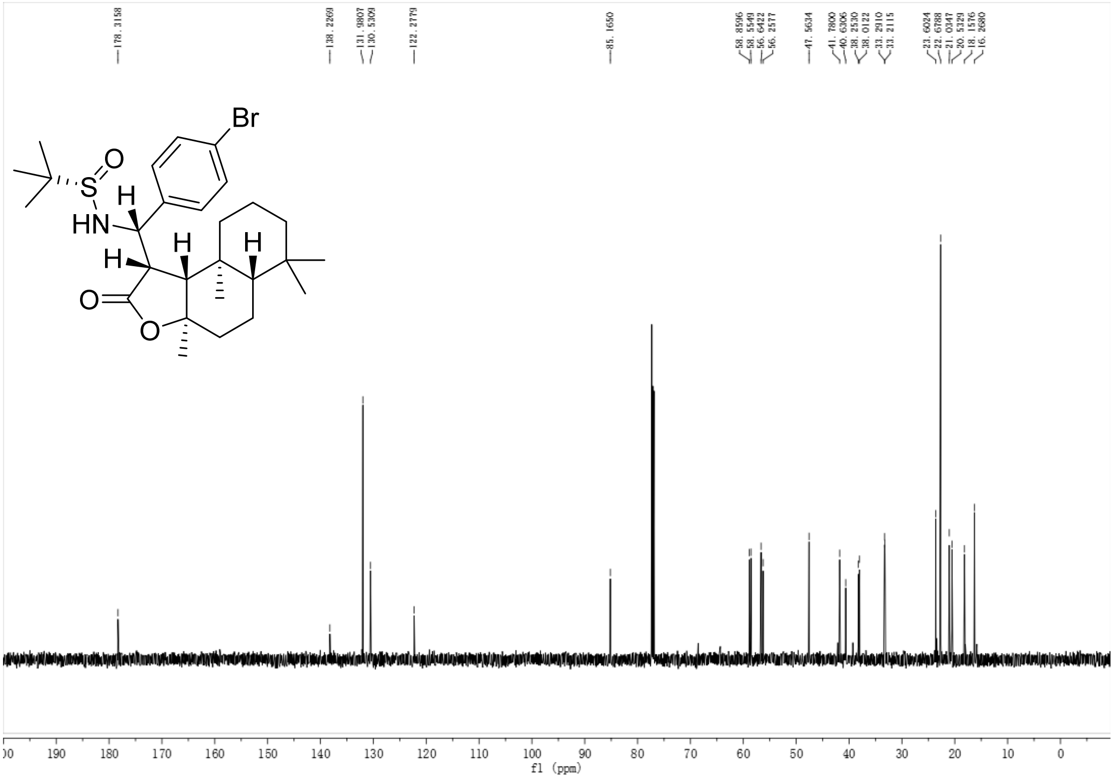

$^1H$ NMR (400 MHz, $CDCl_3$) of **3i**:

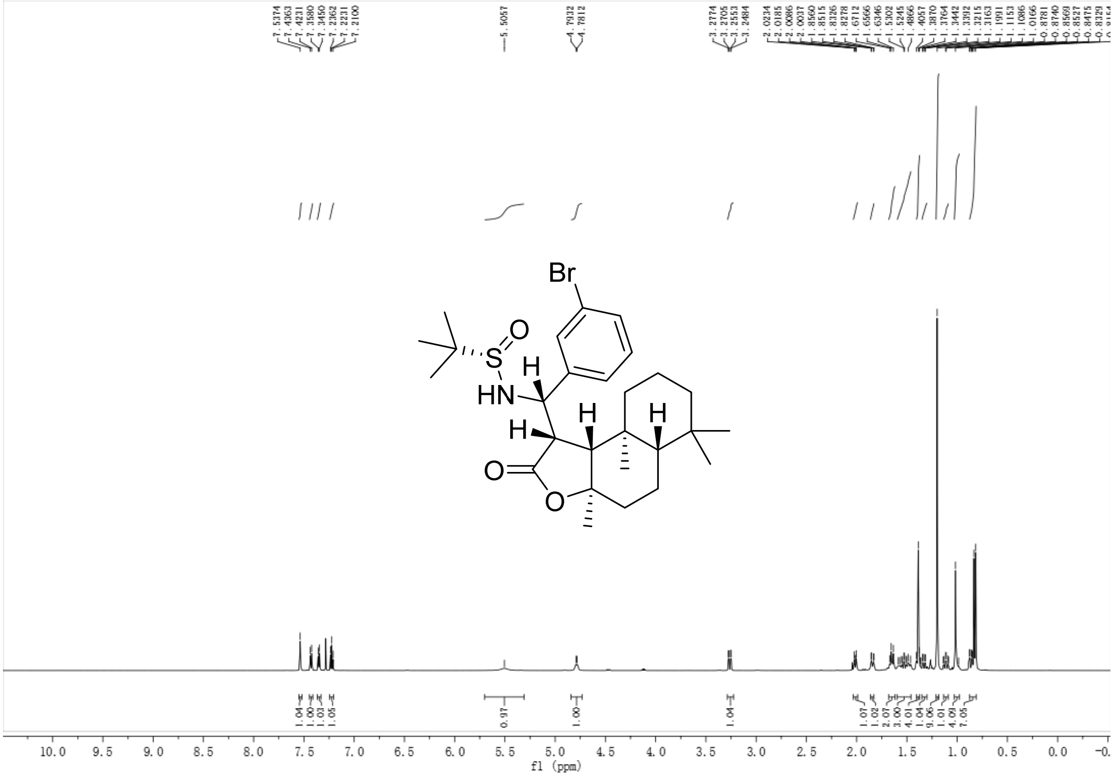

$^{13}C\{^{1}H\}$ NMR (150 MHz, $CDCl_3$) of **3i**:

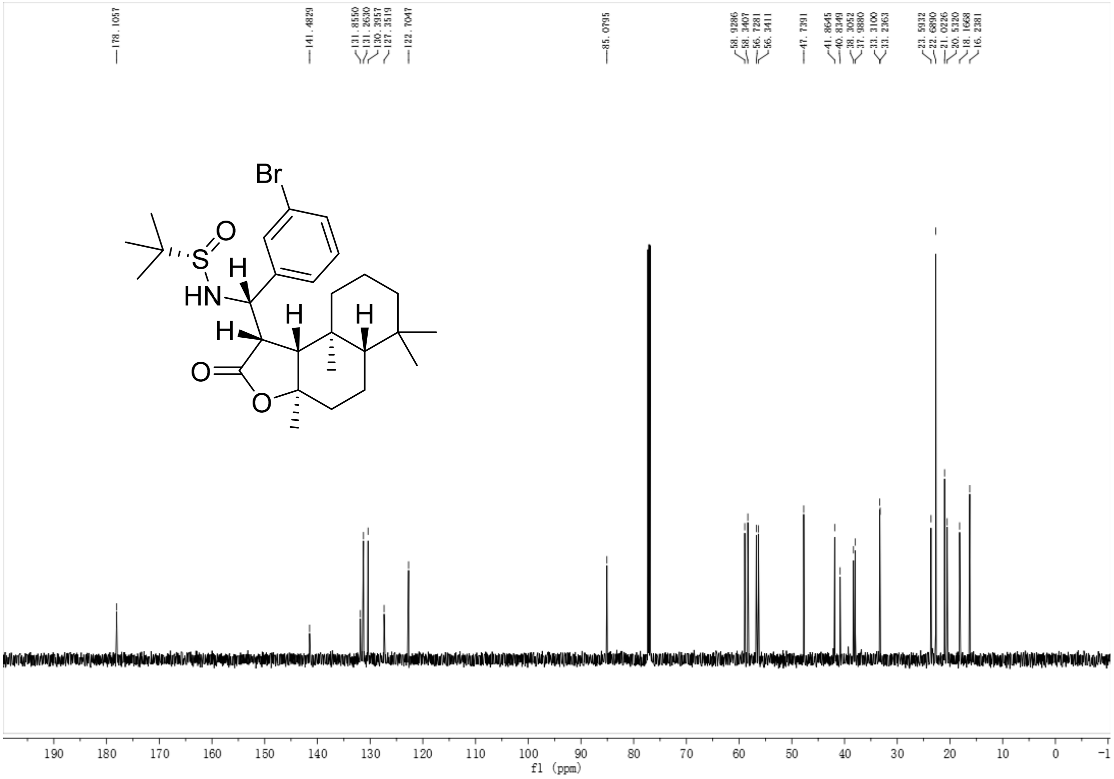

$^{1}H$ NMR (600 MHz, $CDCl_3$) of **3j**:

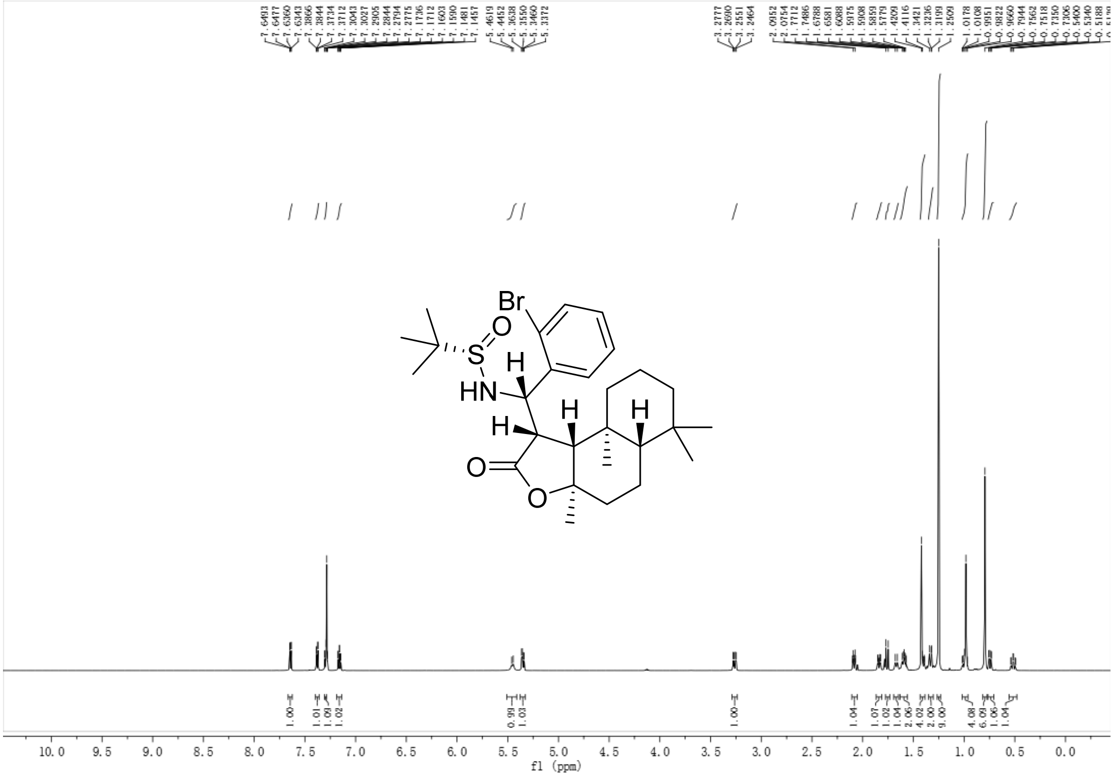

$^{13}C\{^{1}H\}$ NMR (150 MHz, $CDCl_3$) of **3j**:

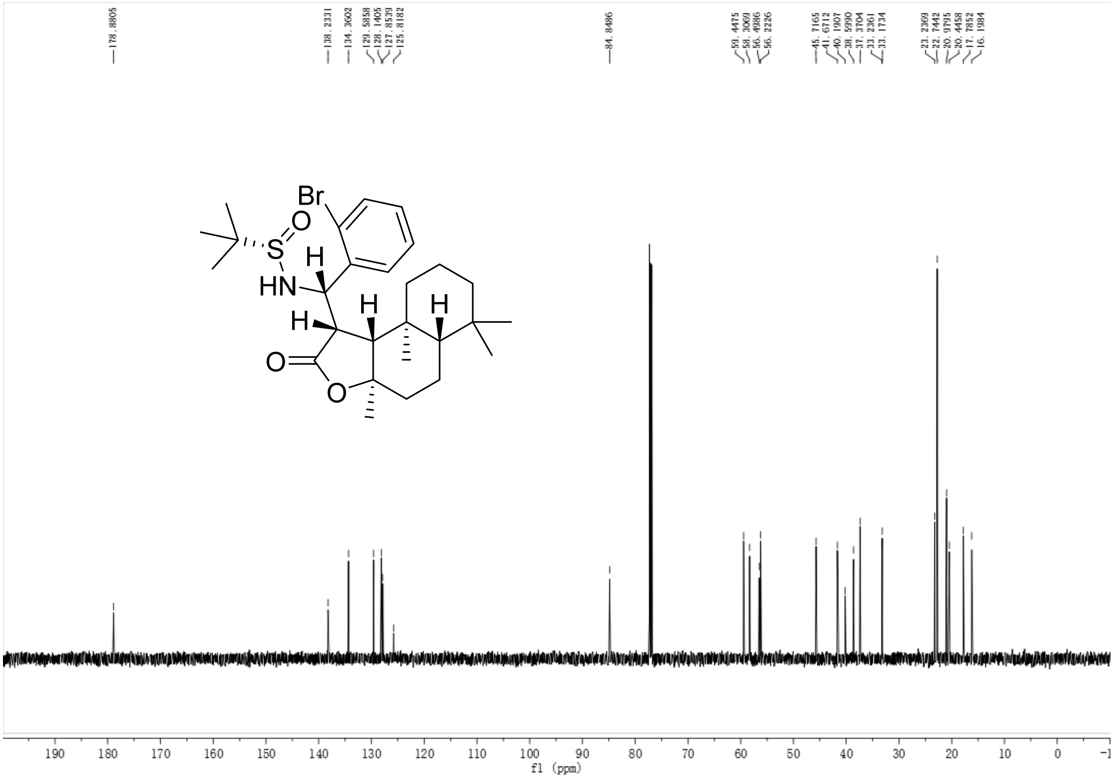

$^{1}H$ NMR (600 MHz, $CDCl_3$) of **3k**:

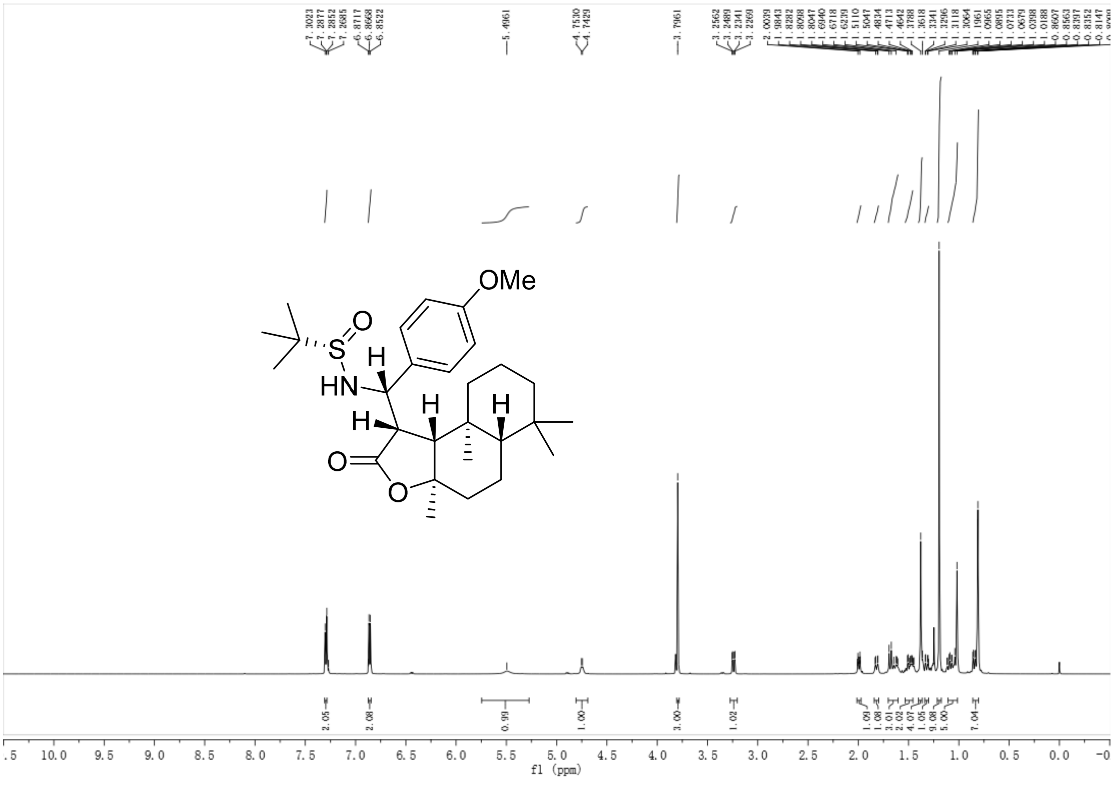

$^{13}C\{^{1}H\}$ NMR (150 MHz, $CDCl_3$) of **3k**:

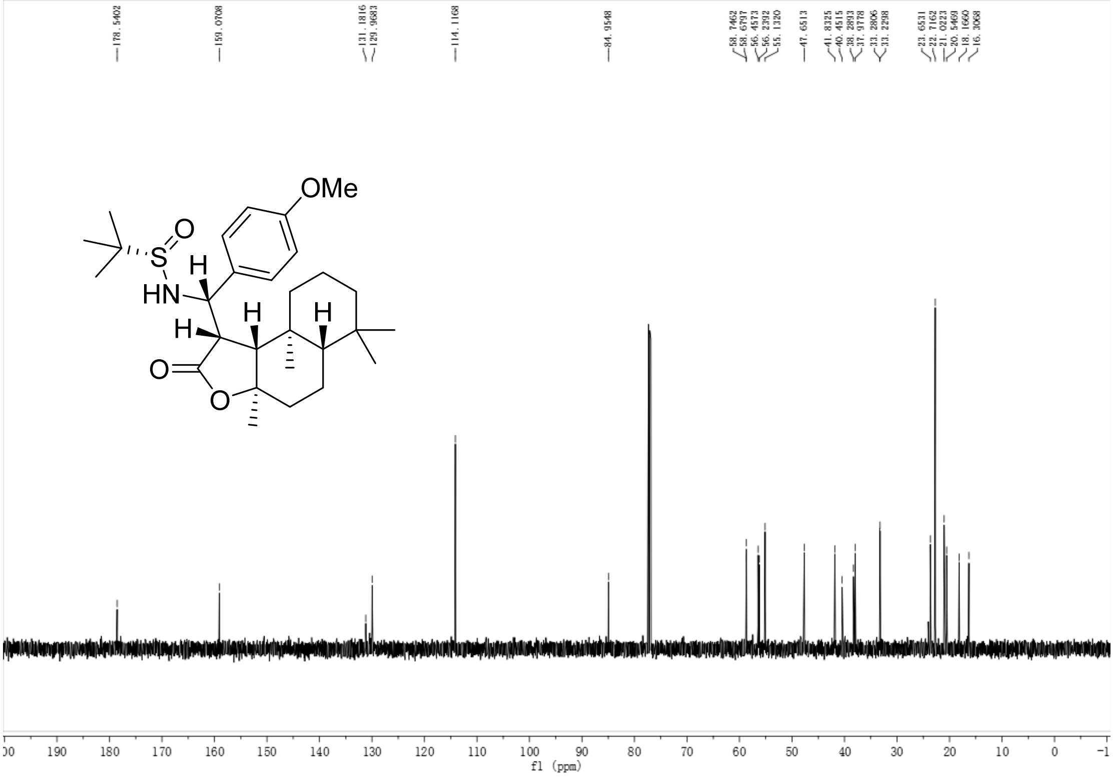

$^{1}H$ NMR (600 MHz, $CDCl_3$) of **3l**:

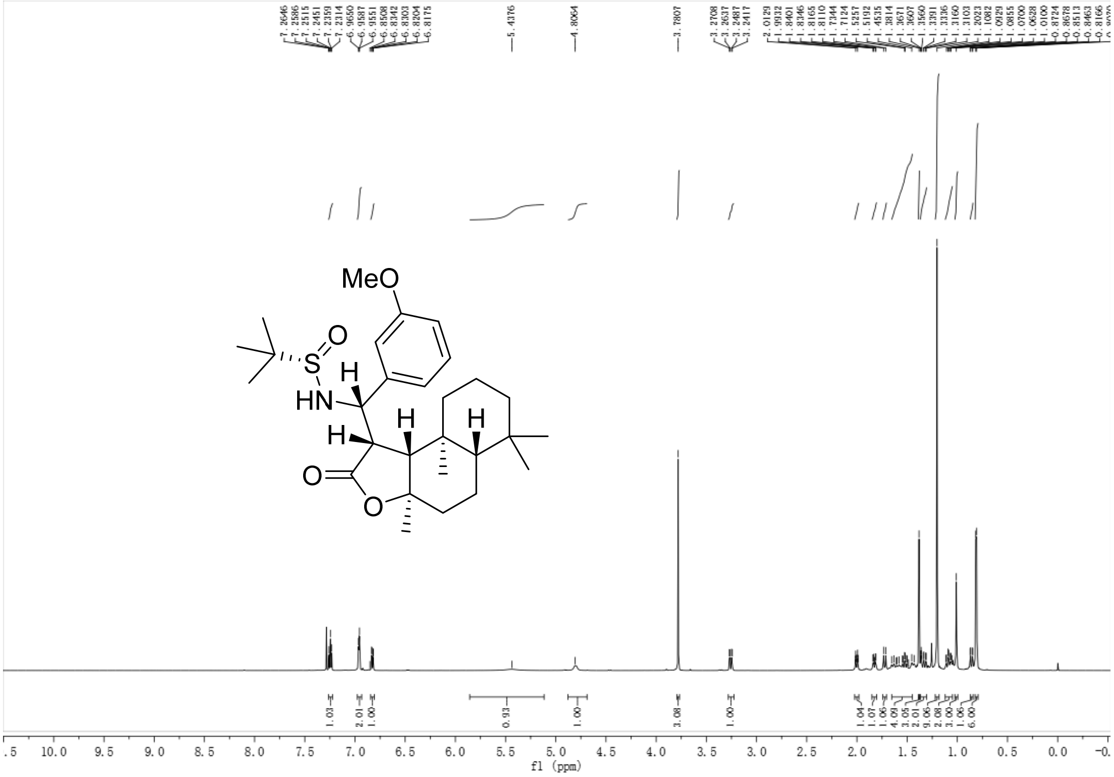

$^{13}C\{^{1}H\}$ NMR (150 MHz, $CDCl_3$) of **3l**:

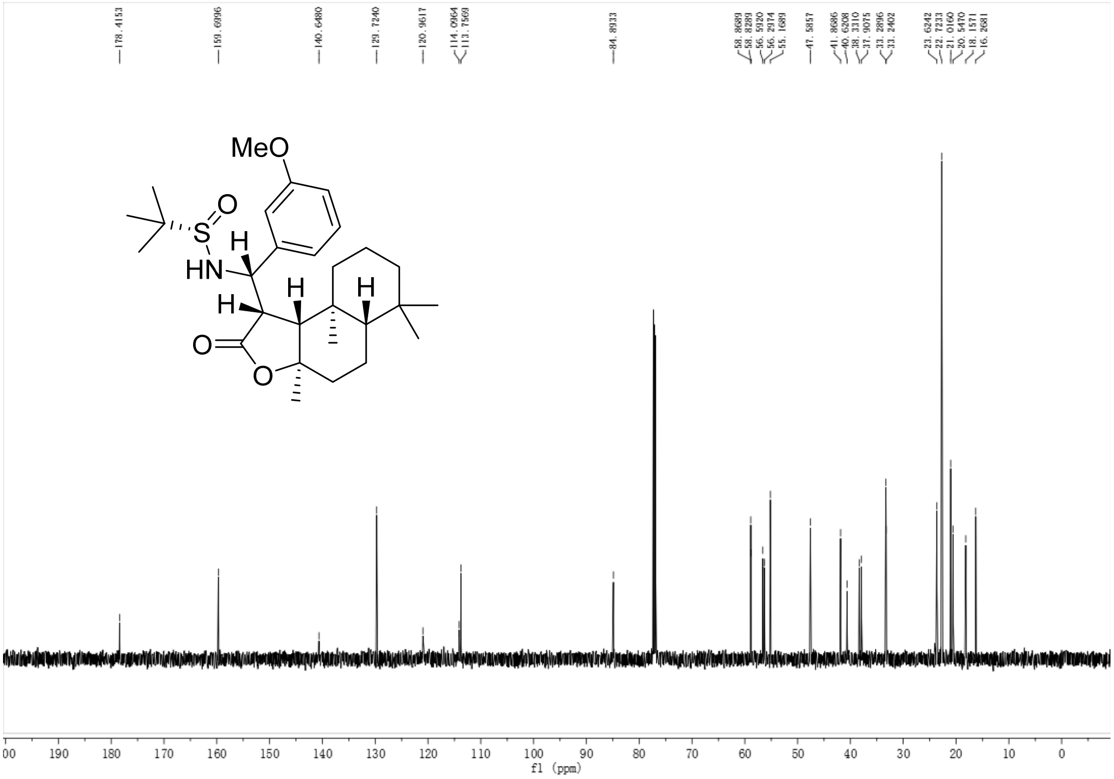

$^{1}H$ NMR (600 MHz, $CDCl_3$) of **3m**:

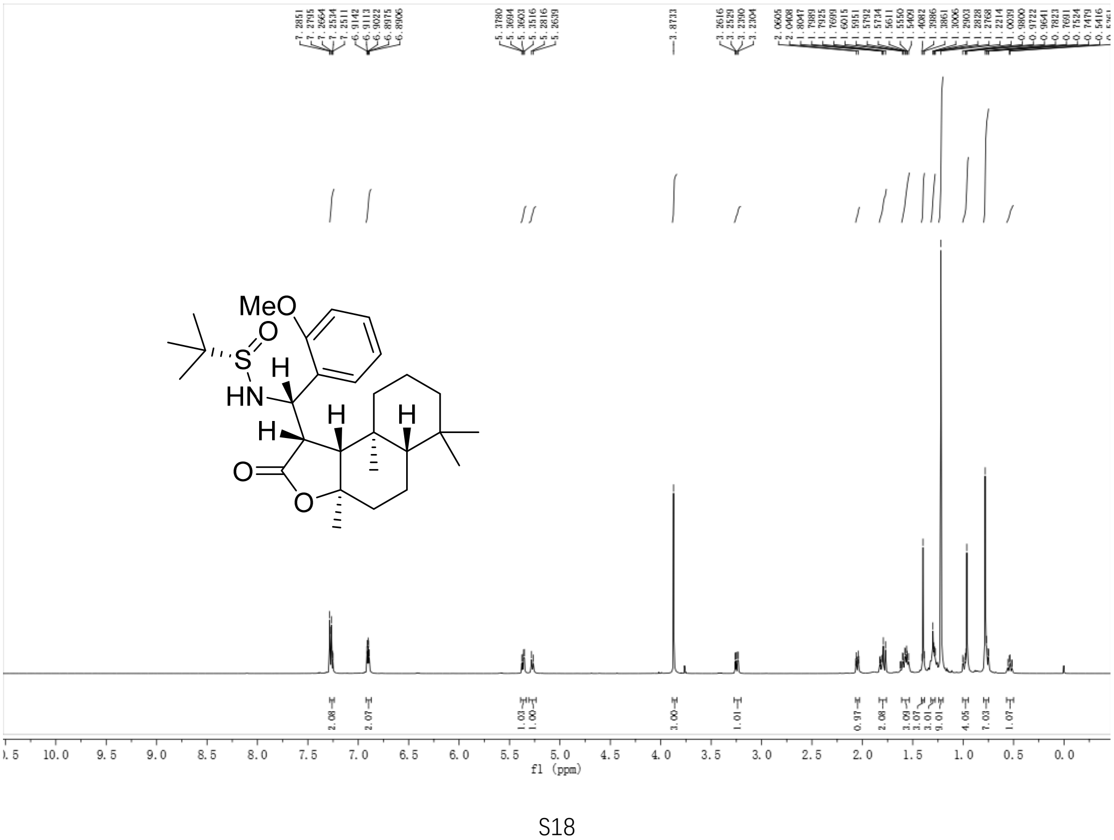

$^{13}C\{^{1}H\}$ NMR (150 MHz, $CDCl_3$) of **3m**:

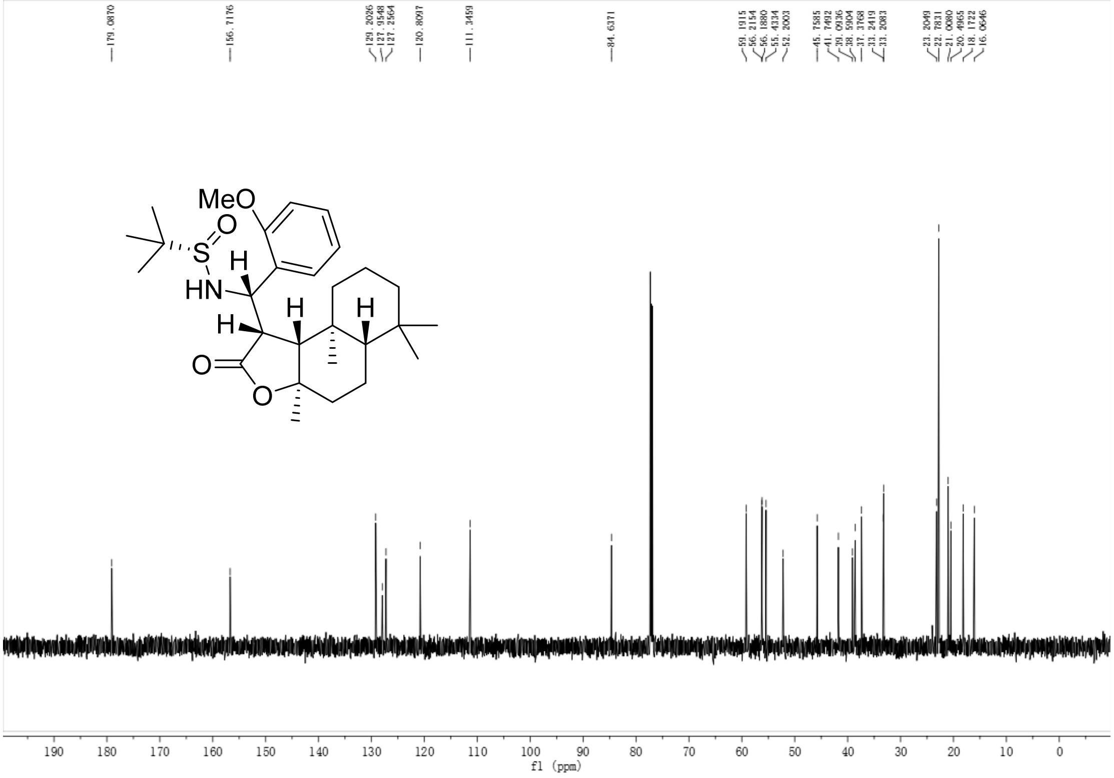


$^{1}H$ NMR (400 MHz, $CDCl_3$) of **3n**:

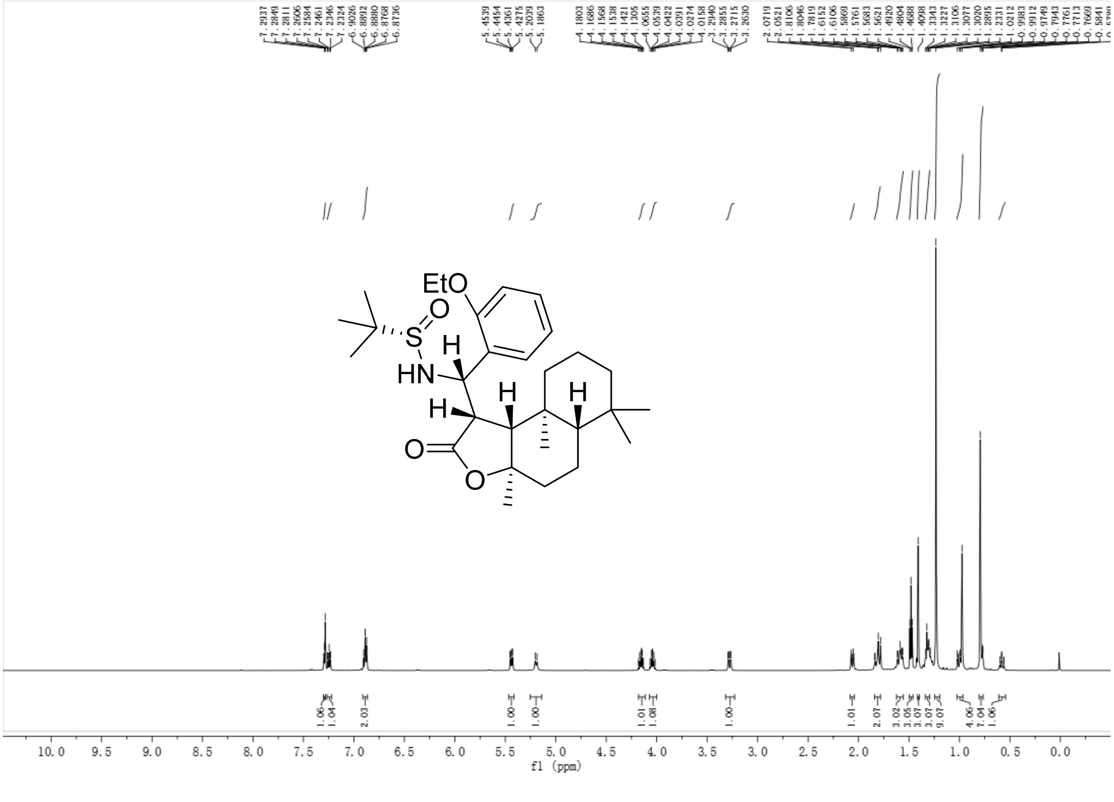

$^{13}C\{^{1}H\}$ NMR (150 MHz, $CDCl_3$) of **3n**:

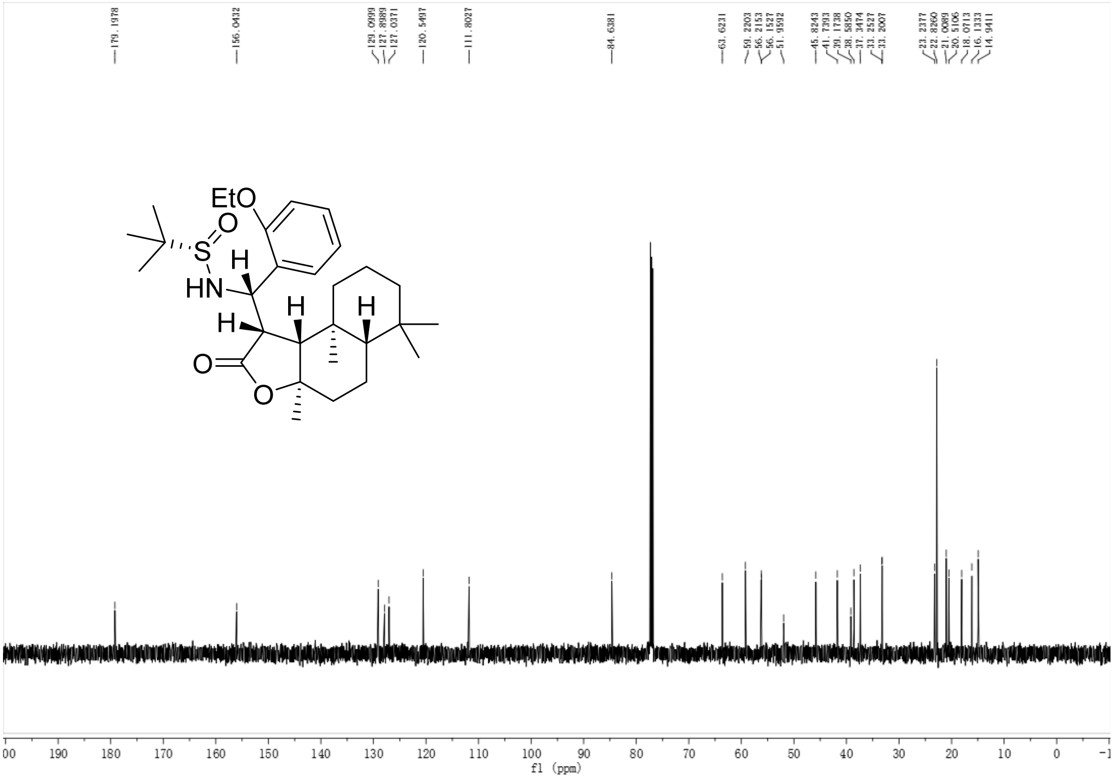


$^{1}H$ NMR (600 MHz, $CDCl_3$) of **3o**:

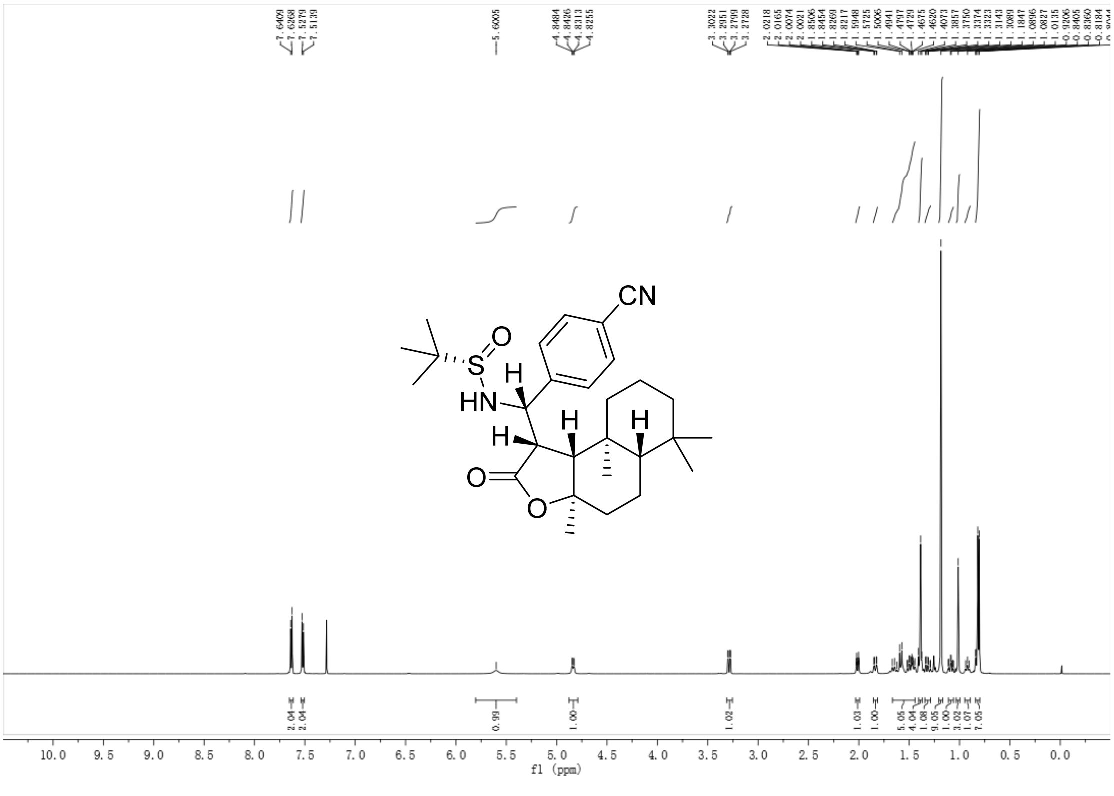

$^{13}C\{^1H\}$ NMR (150 MHz, $CDCl_3$) of **3o**:

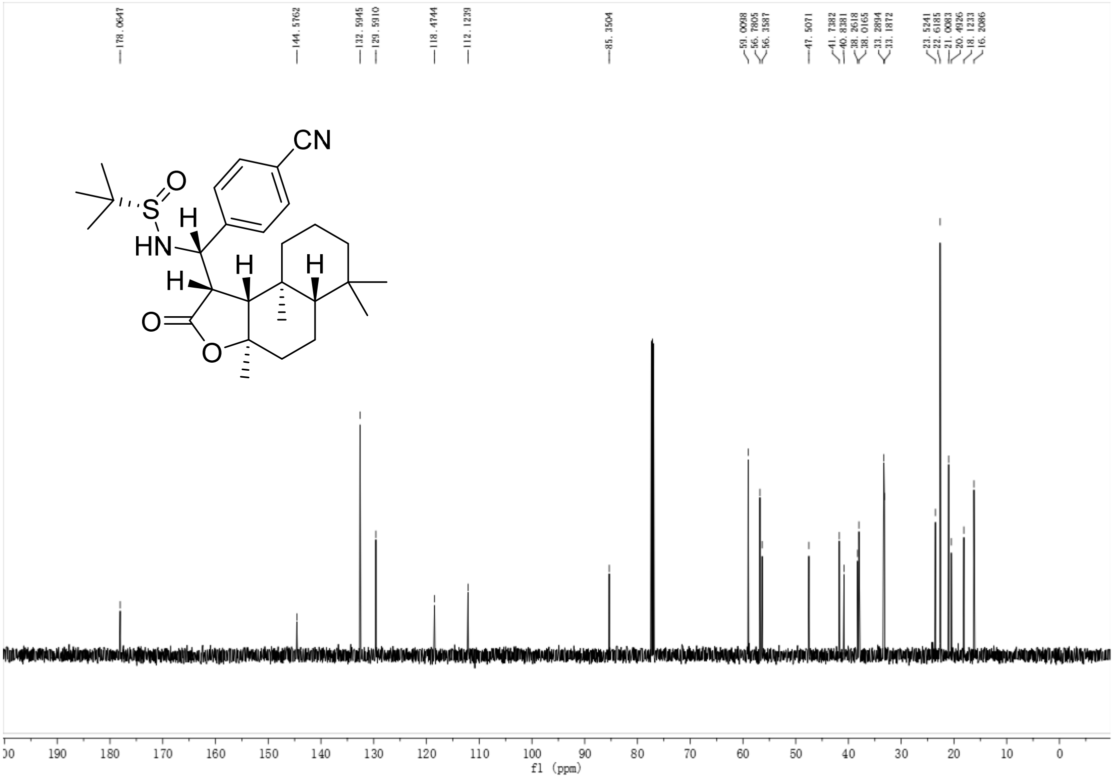


$^1H$ NMR (600 MHz, $CDCl_3$) of **3p**:

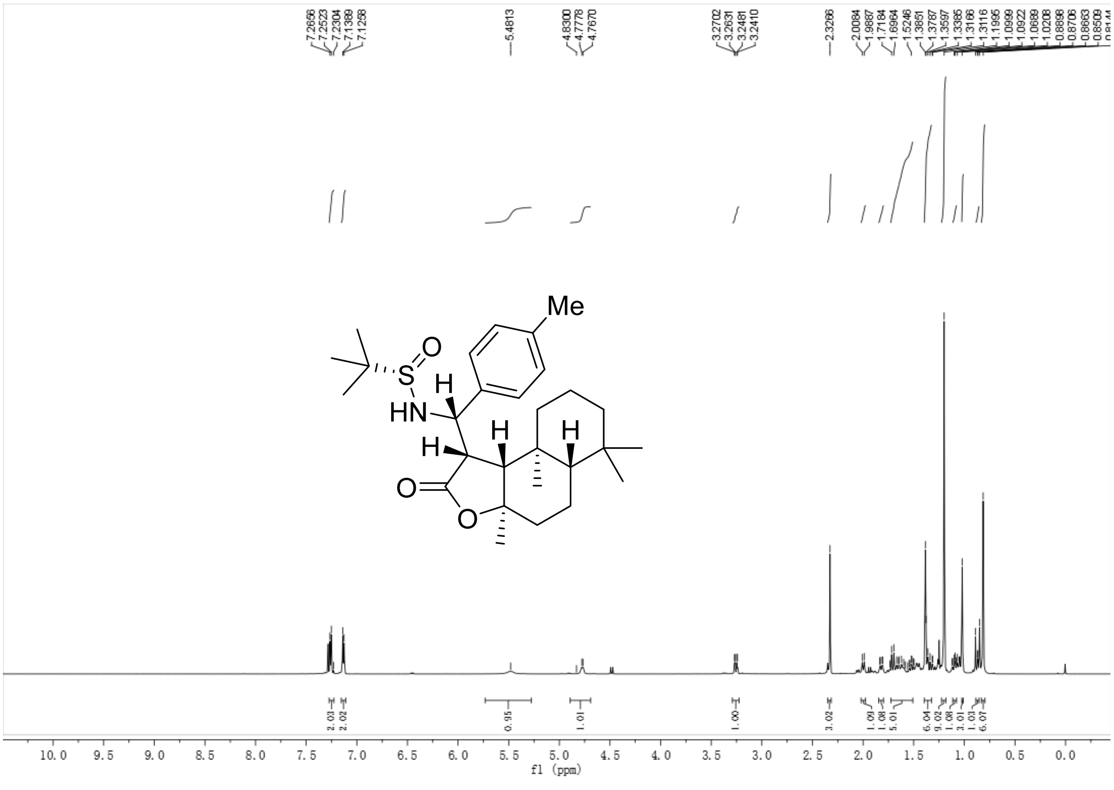

$^{13}C\{^{1}H\}$ NMR (100 MHz, $CDCl_3$) of **3p**:

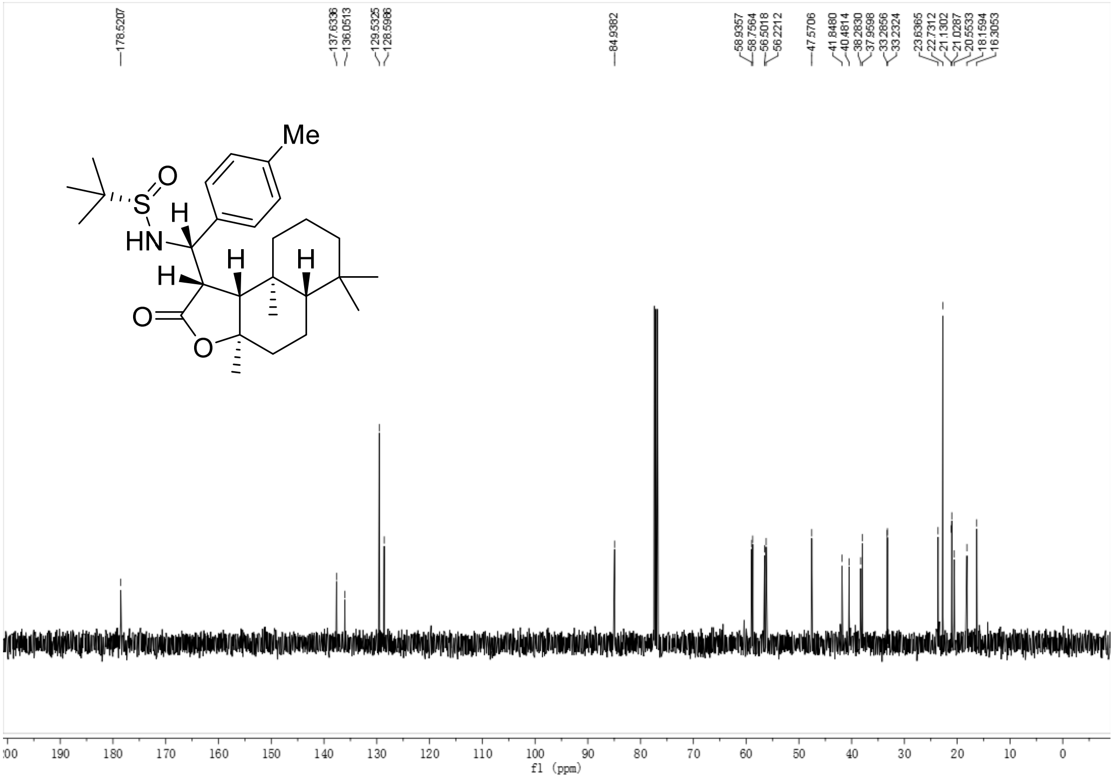


$^{1}H$ NMR (600 MHz, $CDCl_3$) of **3q**:

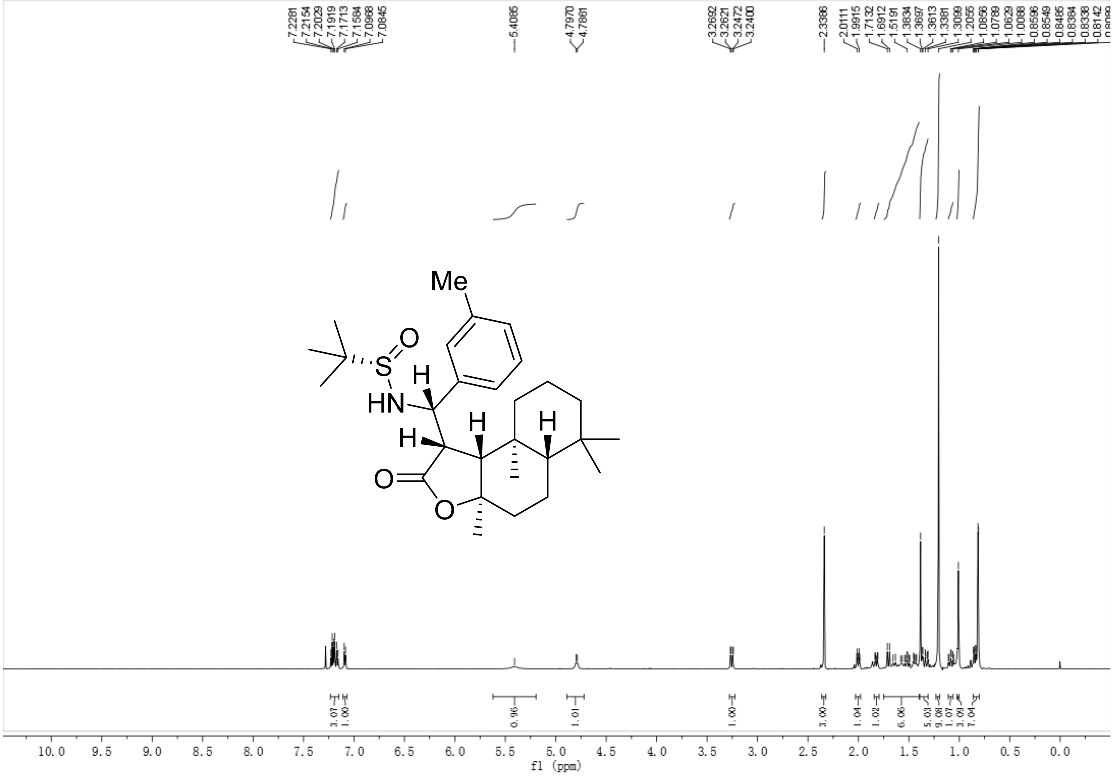

$^{13}C\{^{1}H\}$ NMR (150 MHz, $CDCl_3$) of **3q**:

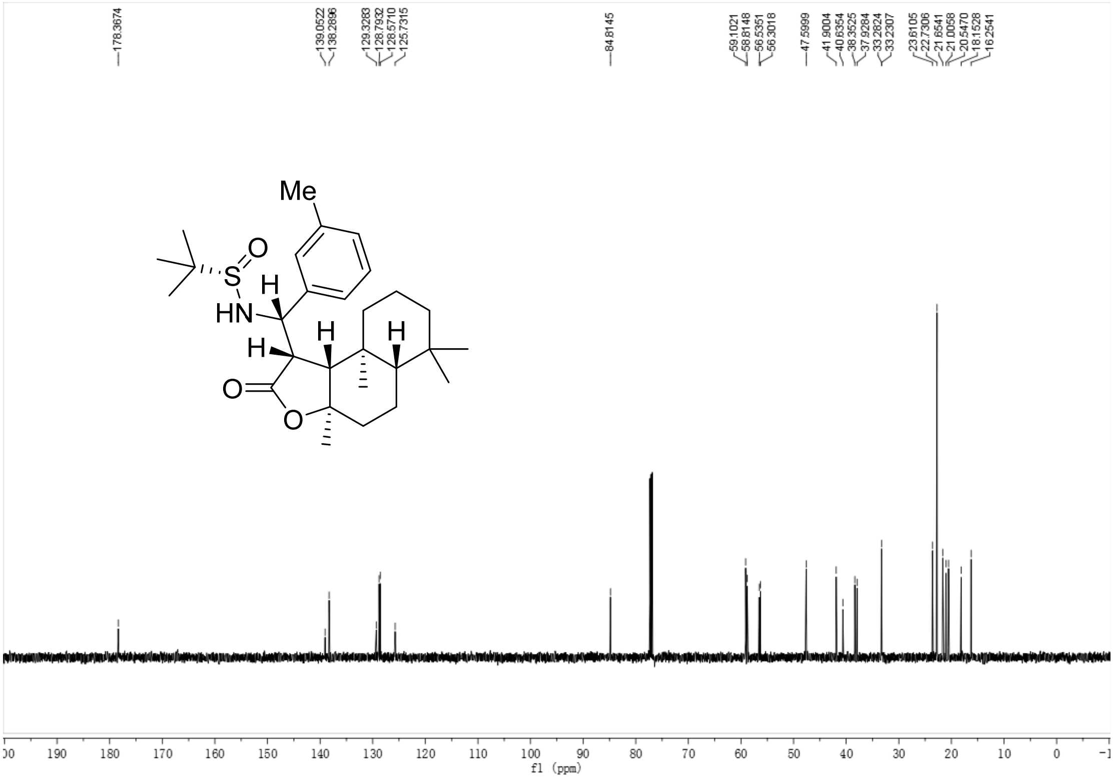


$^{1}H$ NMR (600 MHz, $CDCl_3$) of **3r**:

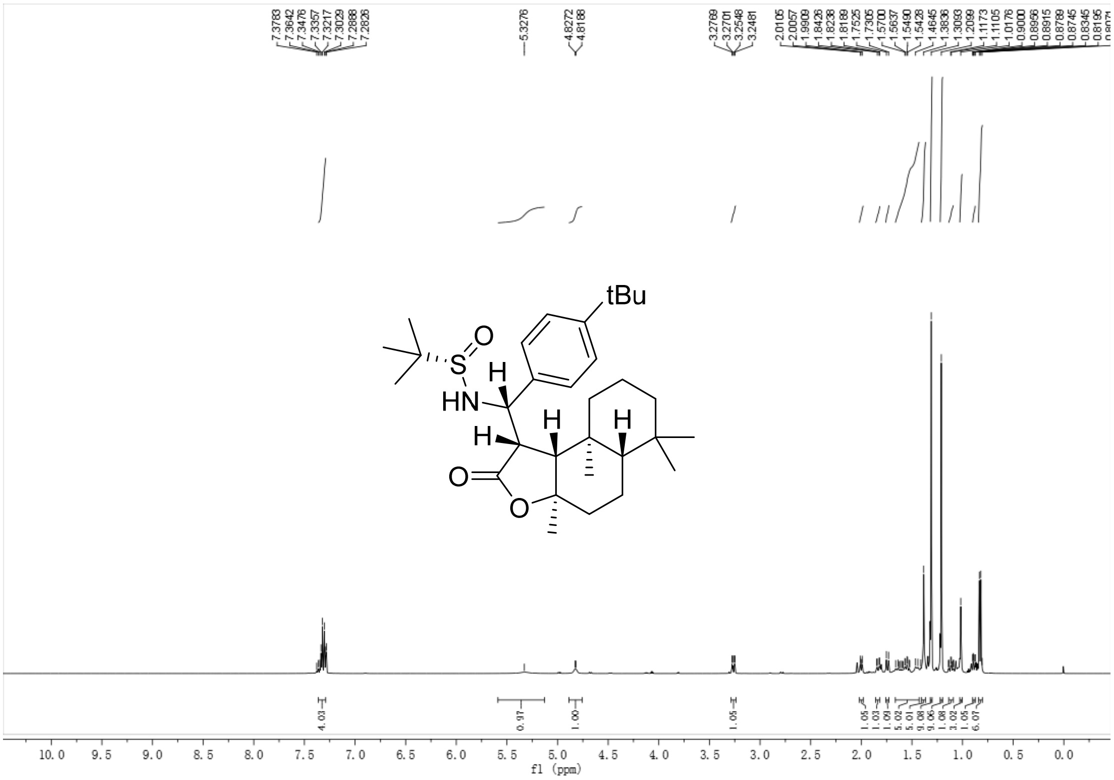

$^{13}C\{^{1}H\}$ NMR (150 MHz, $CDCl_3$) of **3r**:

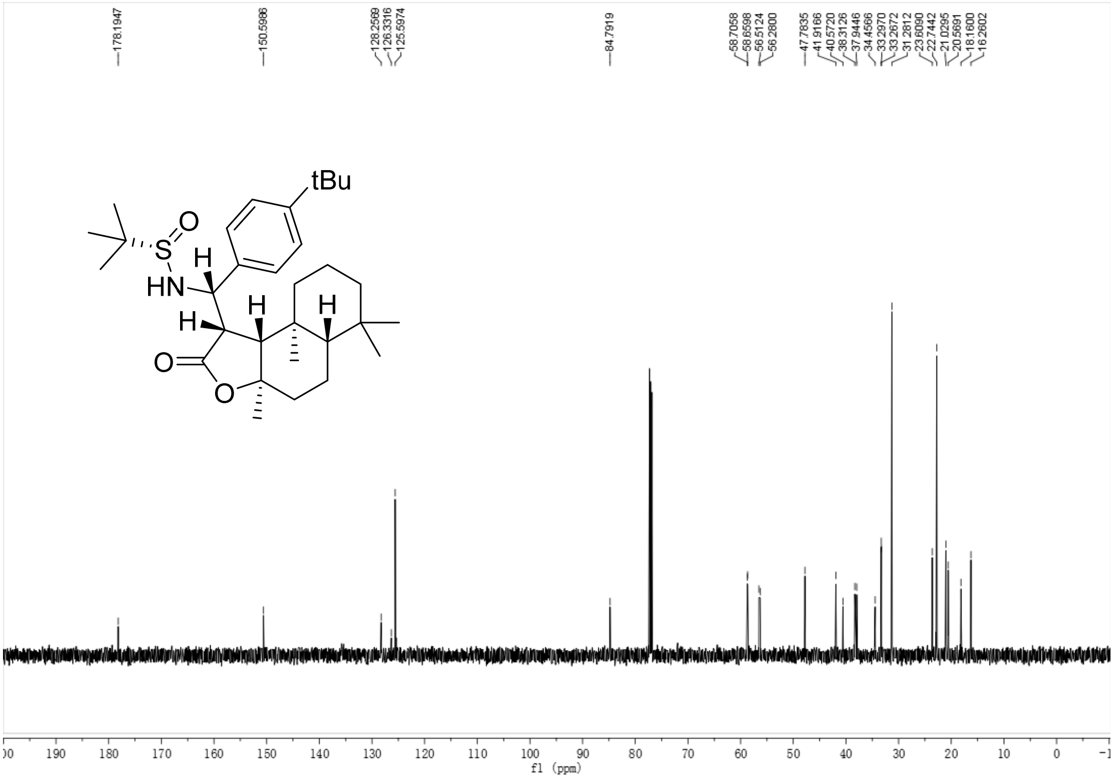


$^{1}H$ NMR (600 MHz, $CDCl_3$) of **3s**:

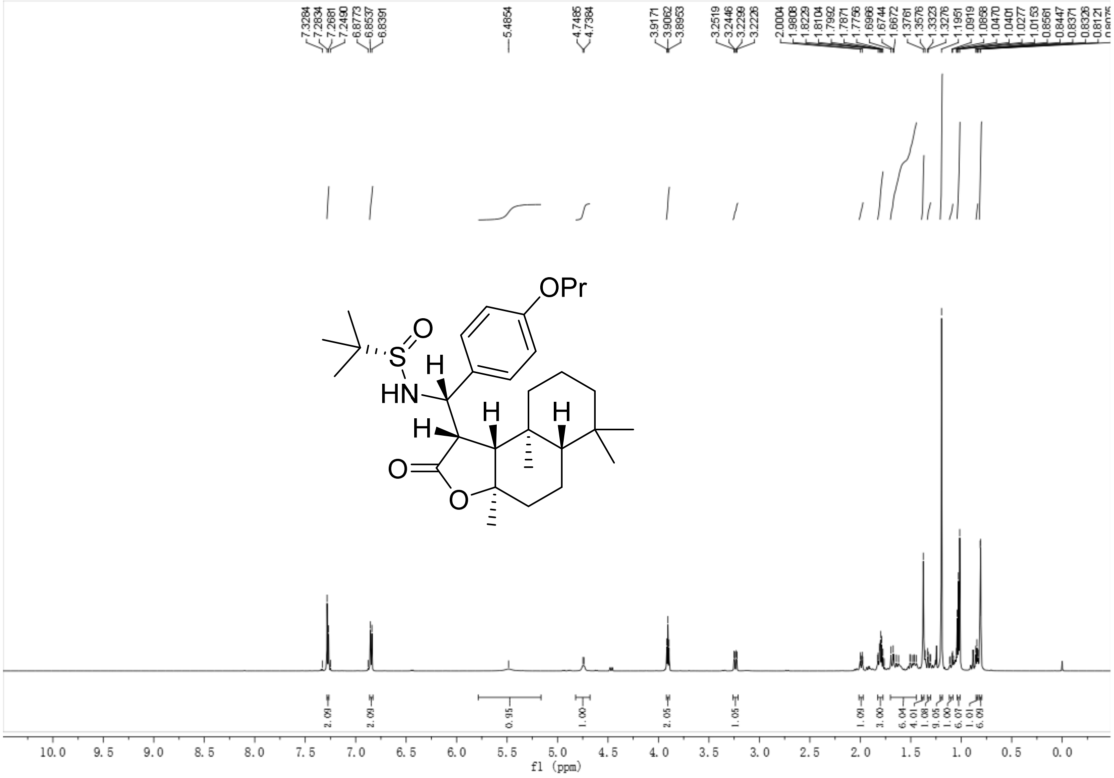

$^{13}C\{^{1}H\}$ NMR (150 MHz, $CDCl_3$) of **3s**:

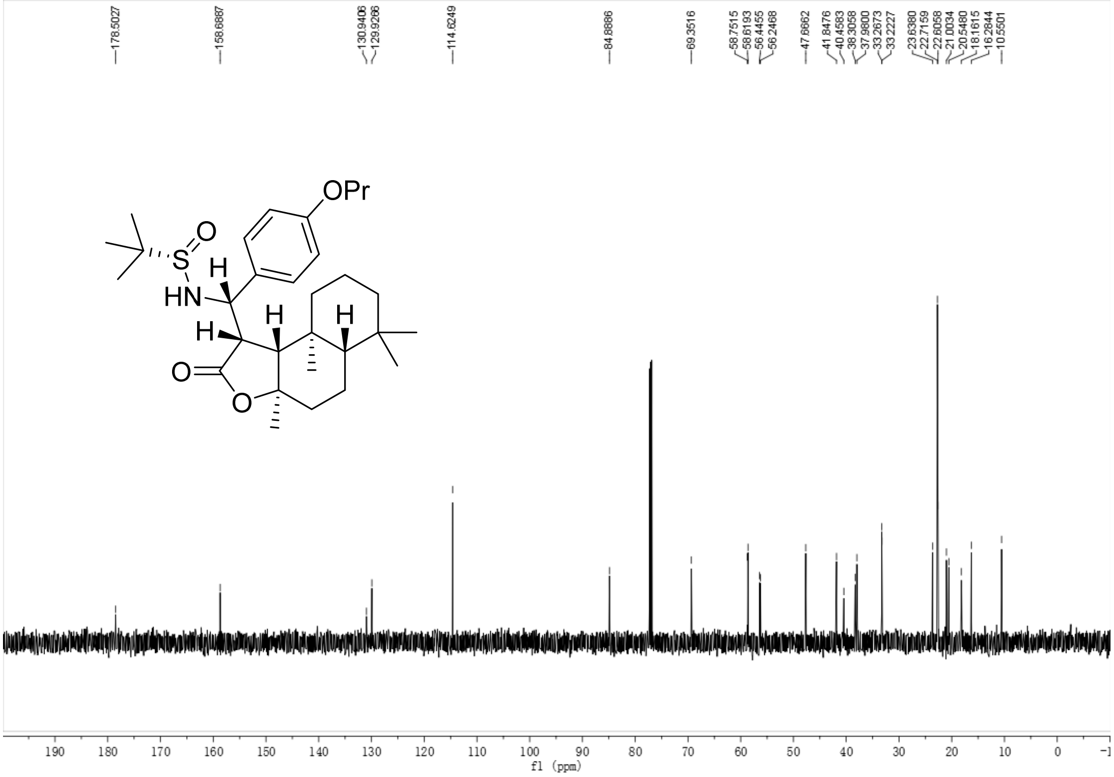

$^{1}H$ NMR (600 MHz, $CDCl_3$) of **3t**:

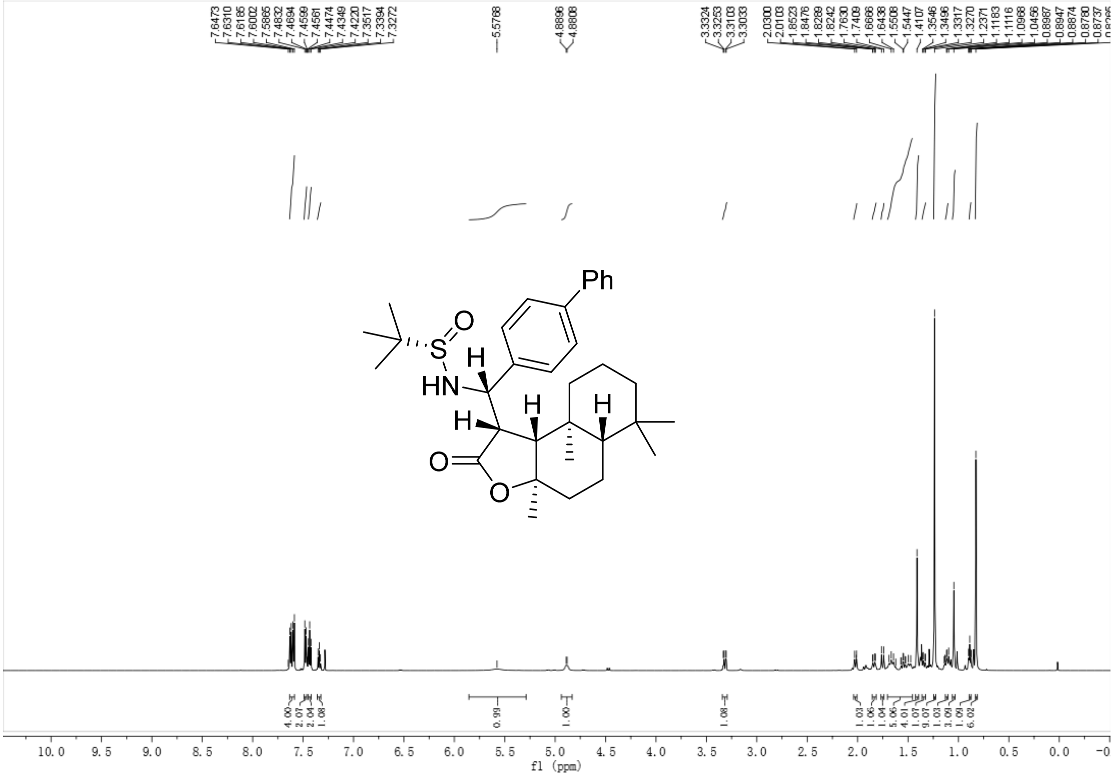

$^{13}C\{^{1}H\}$ NMR (150 MHz, $CDCl_3$) of **3t**:

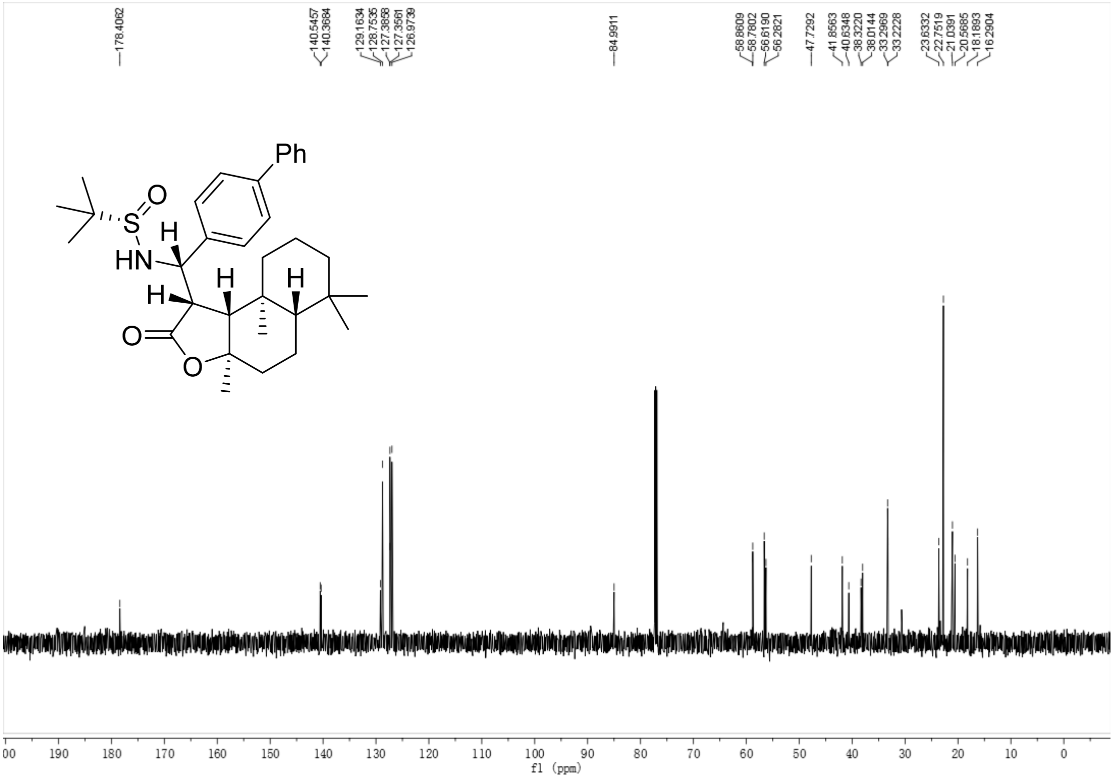


$^{1}H$ NMR (600 MHz, $CDCl_3$) of **3u**:

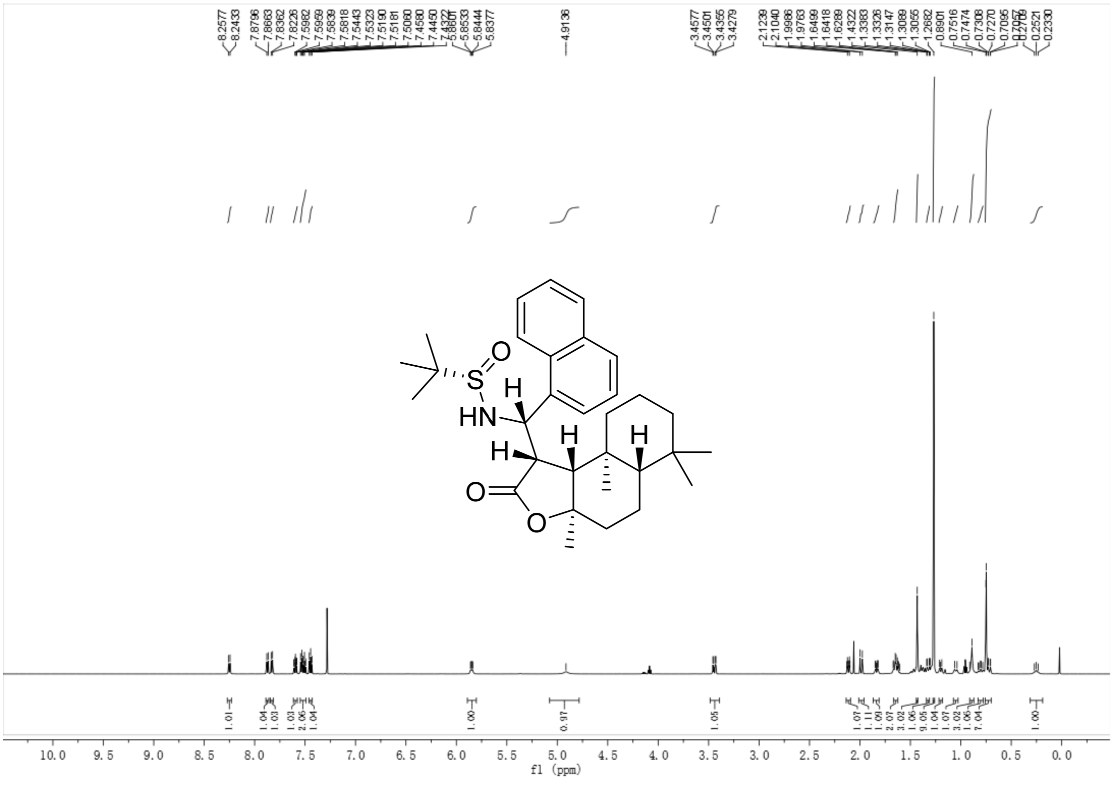

$^{13}C\{^{1}H\}$ NMR (150 MHz, $CDCl_3$) of **3u**:

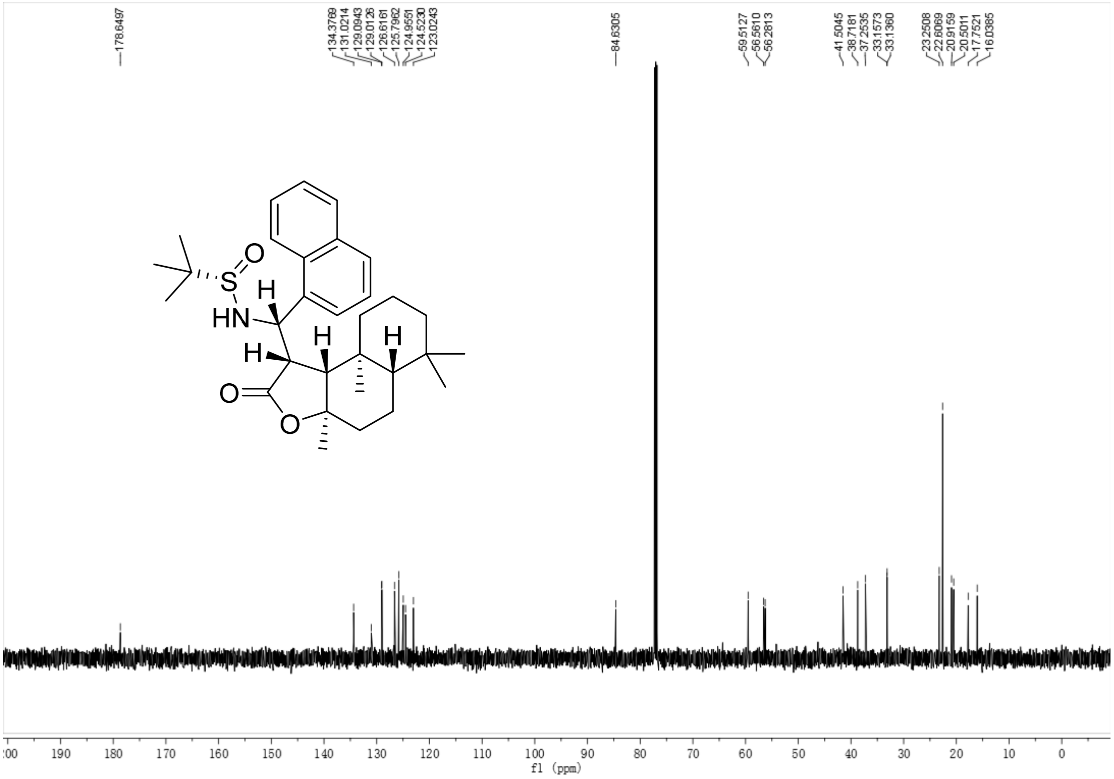

$^{1}H$ NMR (600 MHz, $CDCl_3$) of **3v**:

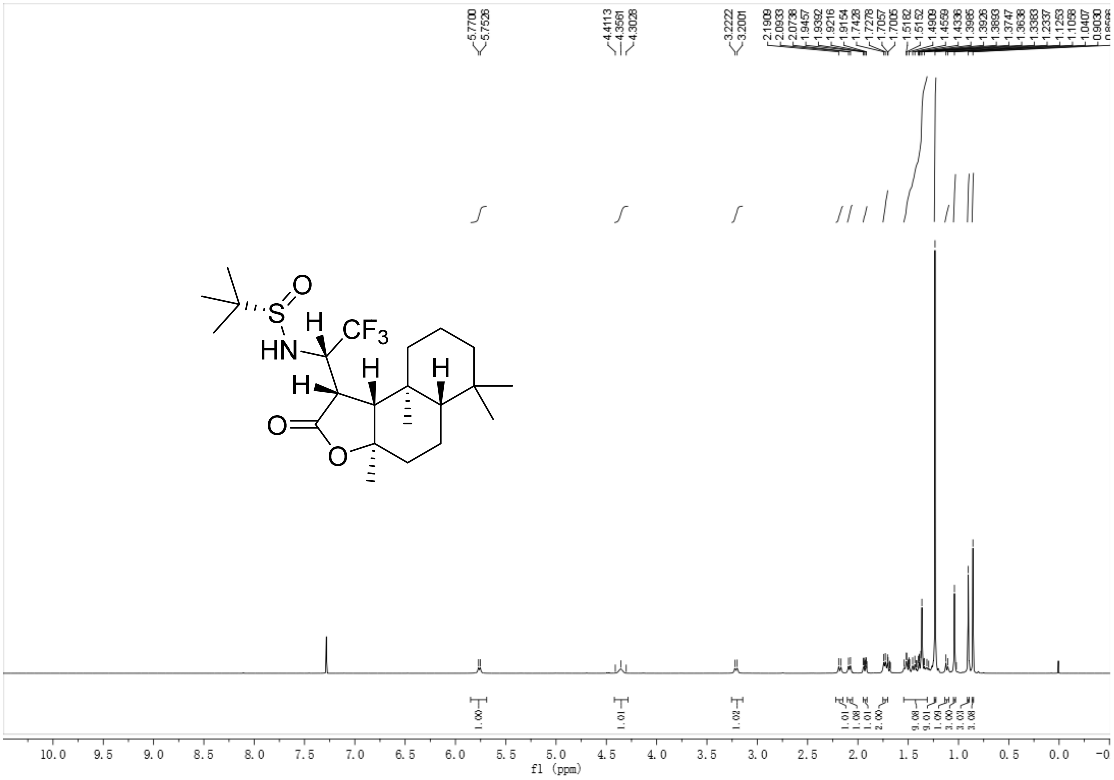

$^{13}C\{^{1}H\}$ NMR (150 MHz, $CDCl_3$) of **3v**:

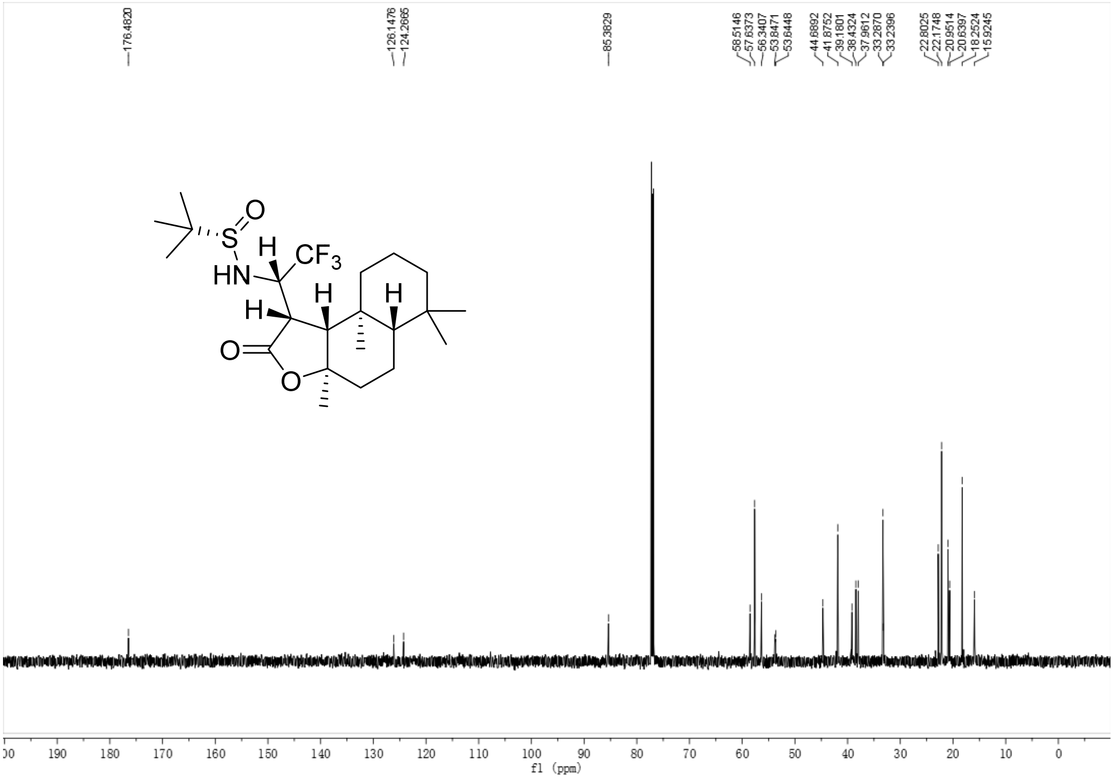


$^{19}F$ NMR (565 MHz, $CDCl_3$) of **3v**:

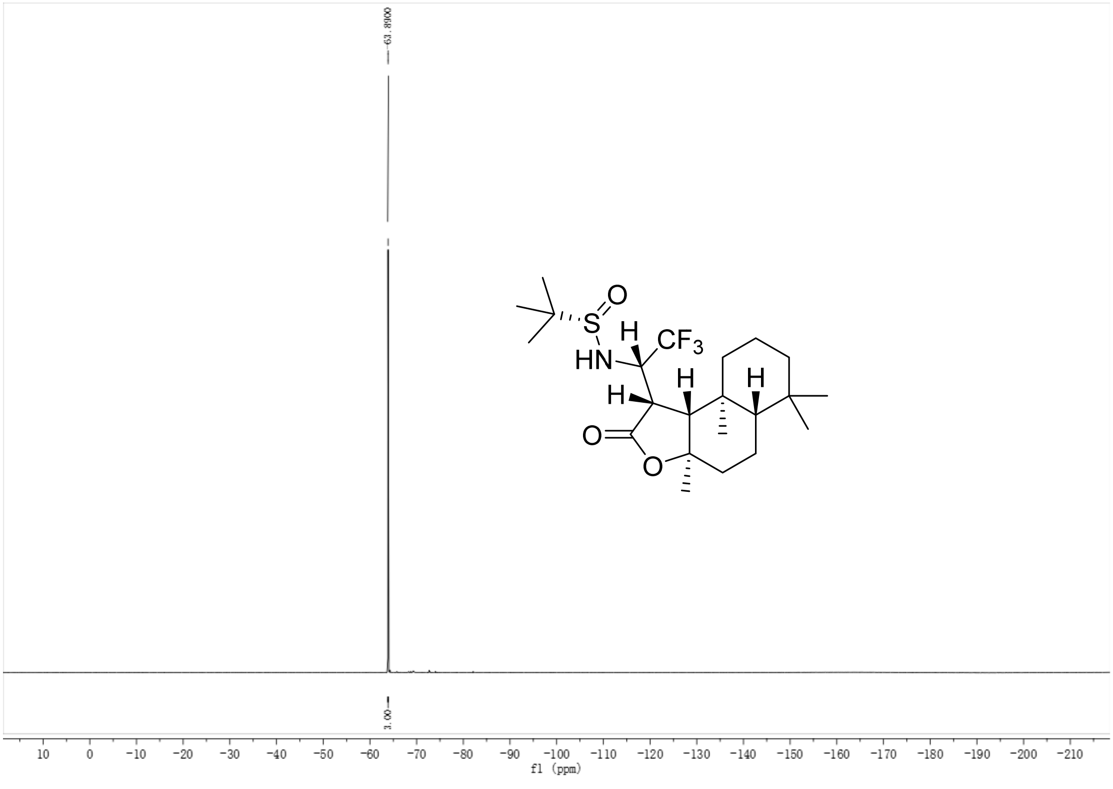

$^{1}H$ NMR (400 MHz, $CDCl_3$) of **4**:

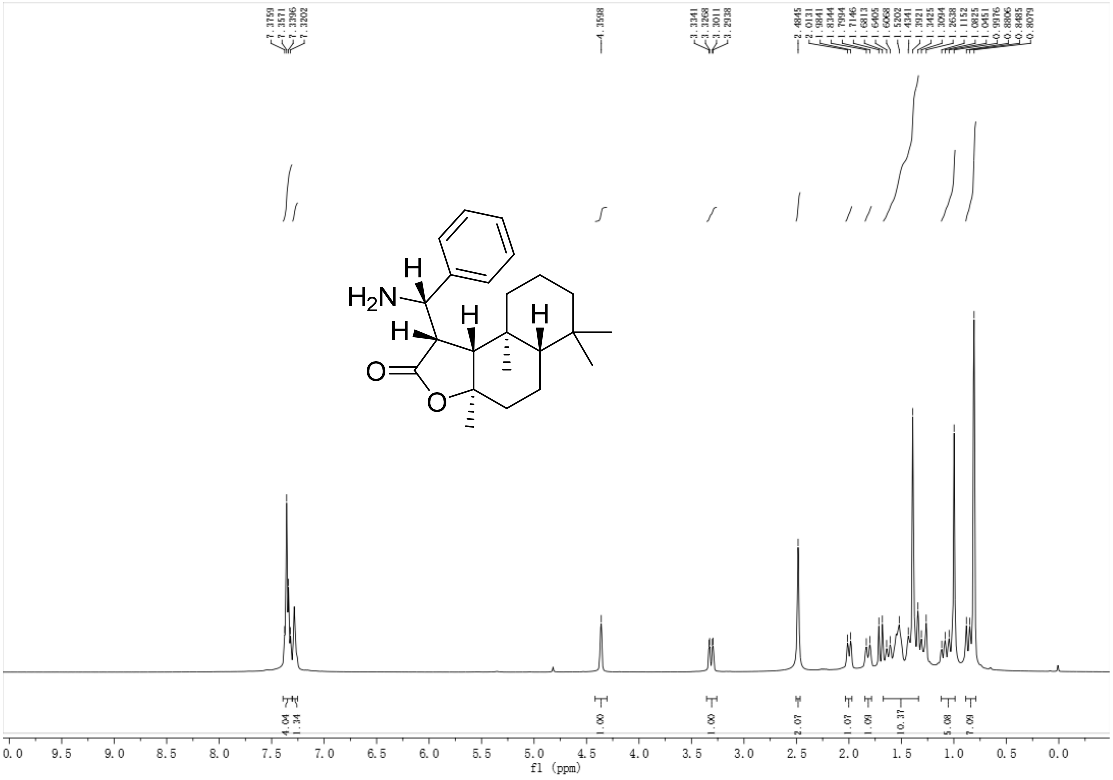

$^{13}C\{^{1}H\}$ NMR (100 MHz, $CDCl_3$) of **4**:

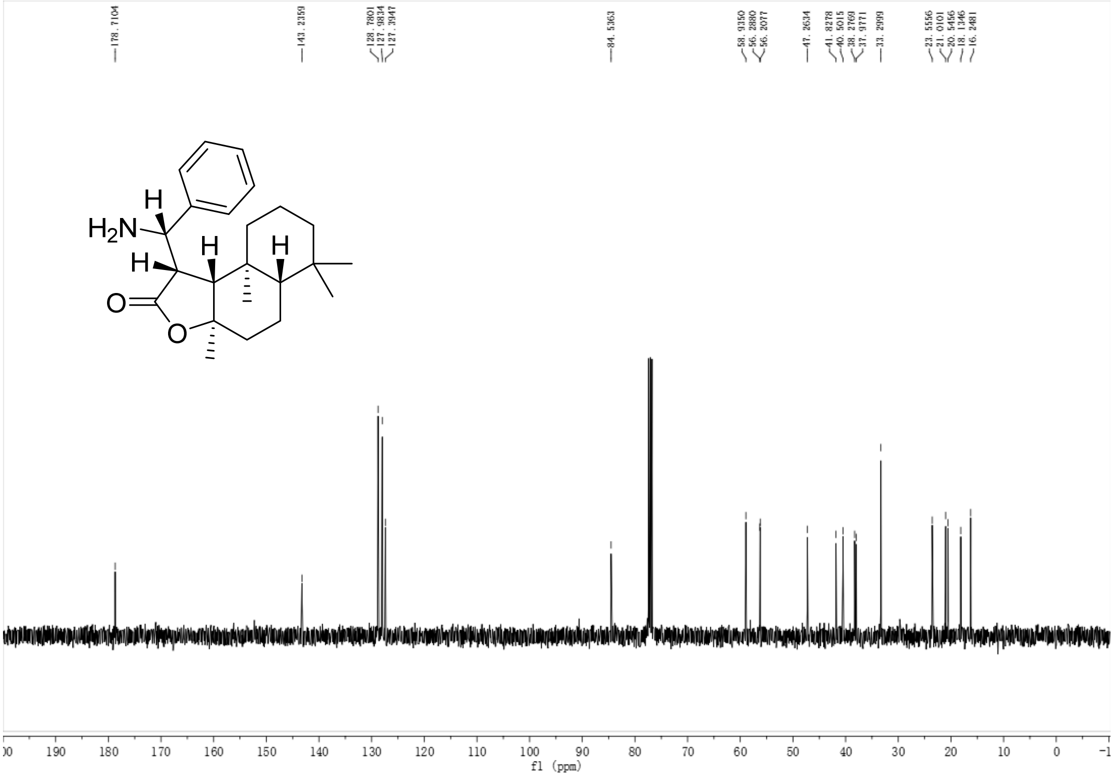

$^{1}$H NMR (400 MHz, $CDCl_3$) of **5**:

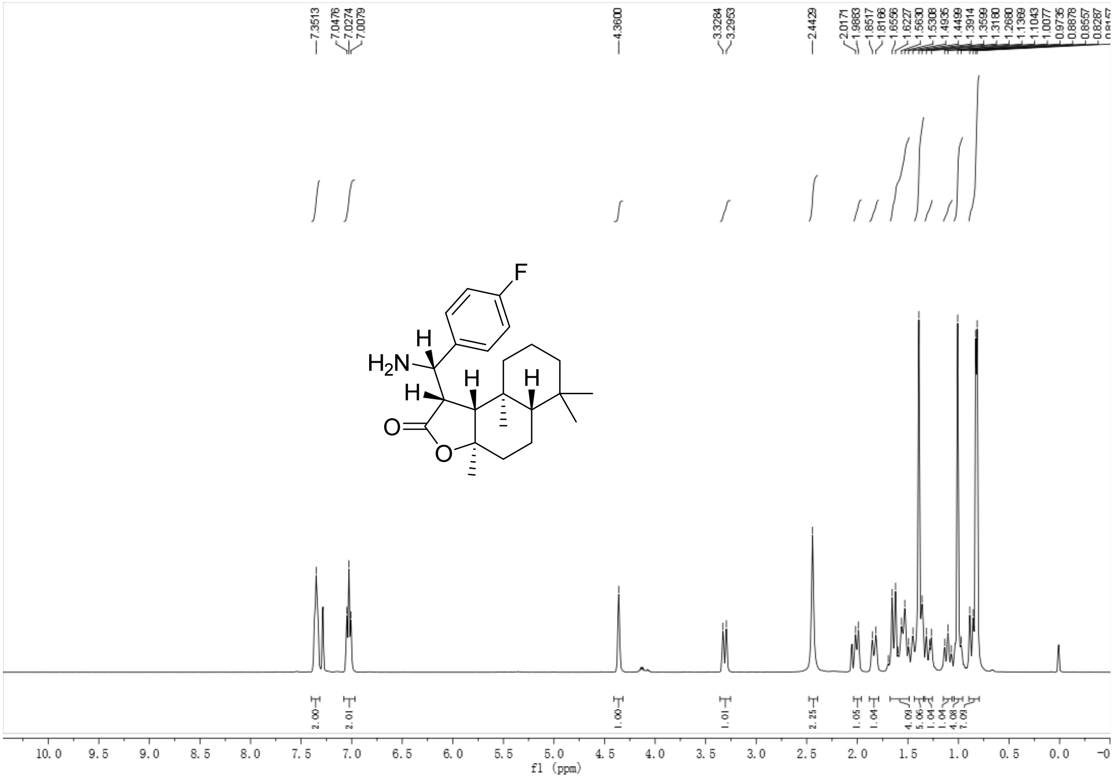

$^{13}$C{$^{1}$H} NMR (100 MHz, $CDCl_3$) of **5**:

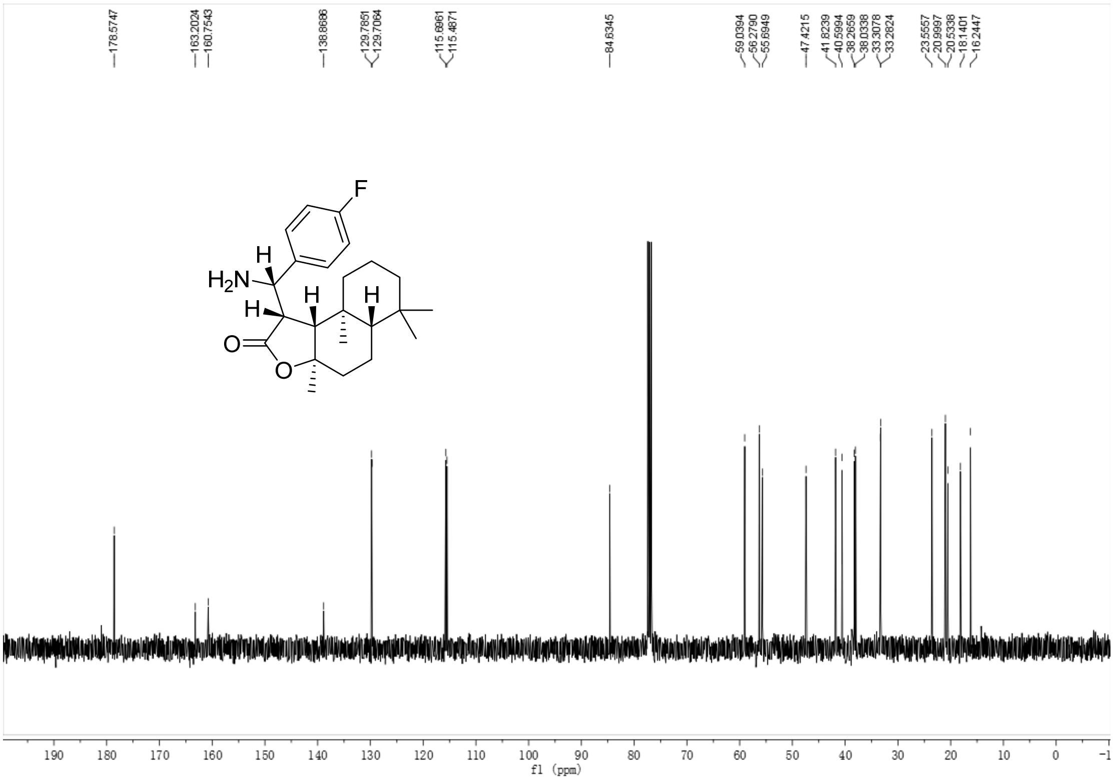

$^{19}$F NMR (376 MHz, $CDCl_3$) of **5**:

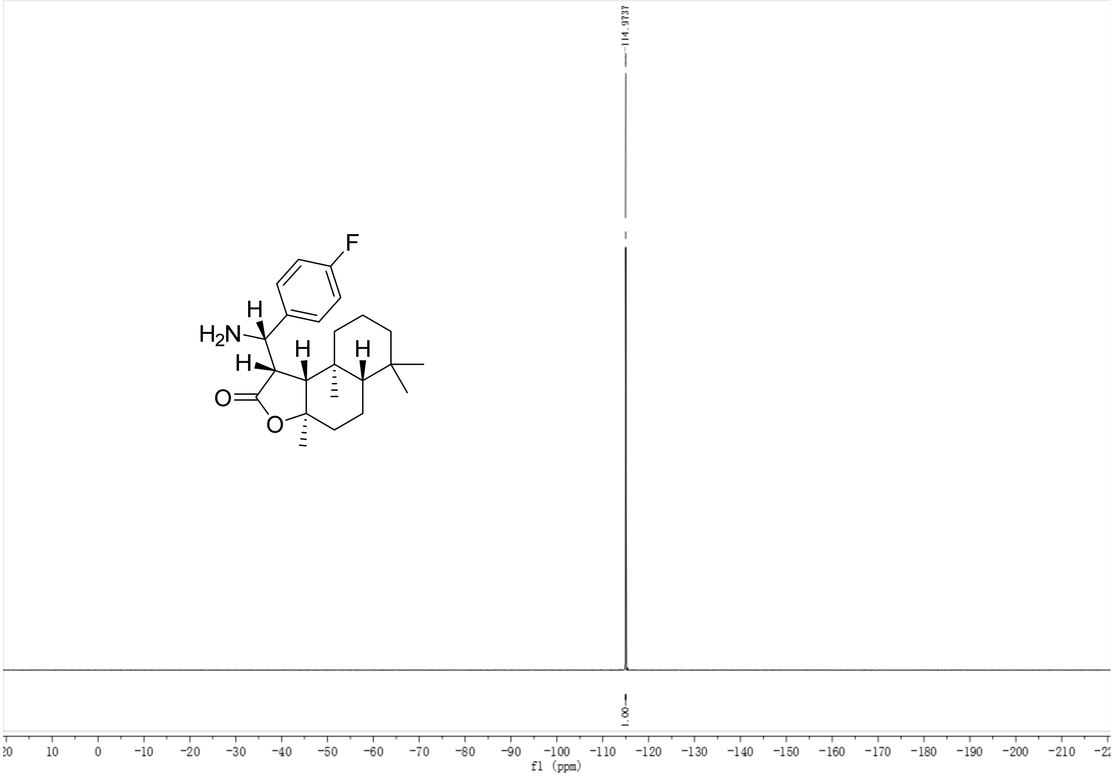


$^{1}$H NMR (400 MHz, $CDCl_3$) of **6**:

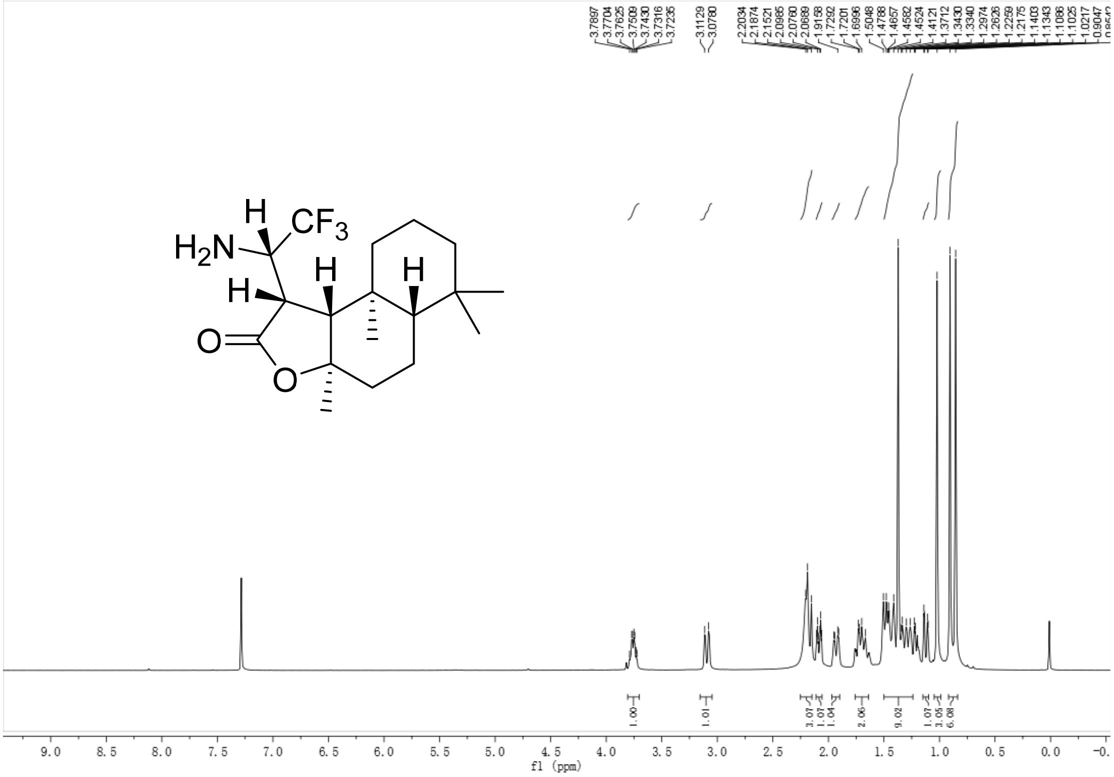

$^{13}C\{^1H\}$ NMR (100 MHz, $CDCl_3$) of **6**:

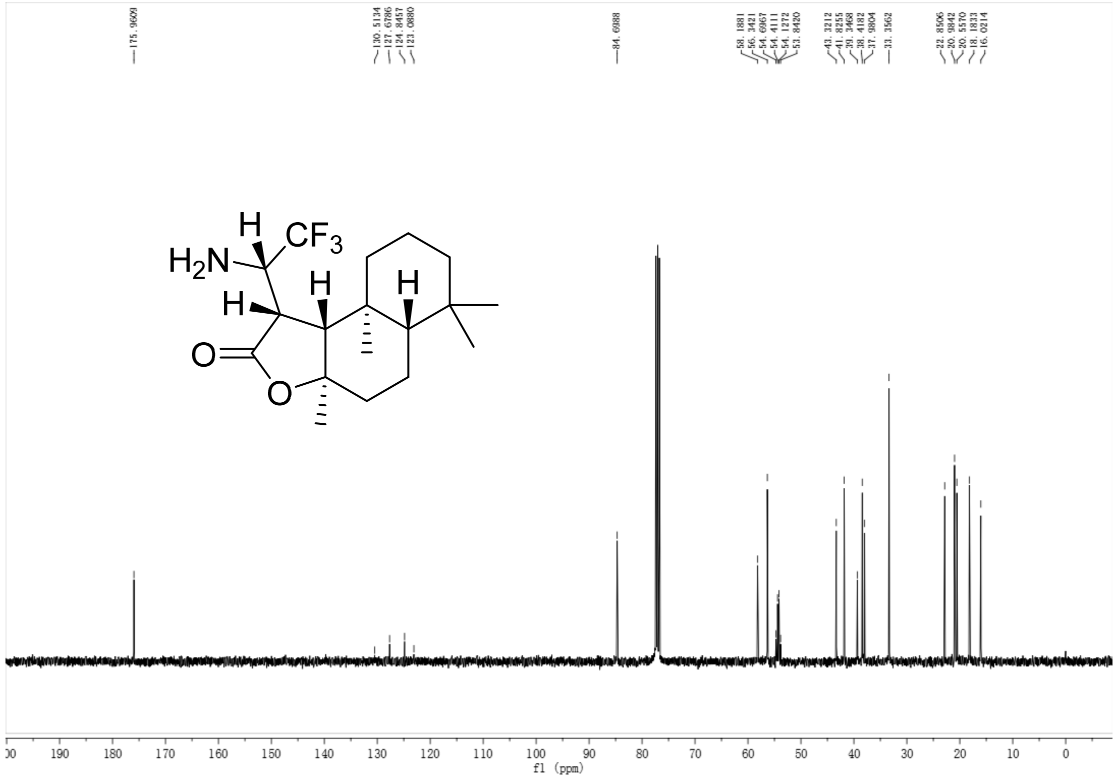

$^{19}F$ NMR (376 MHz, $CDCl_3$) of **6**:

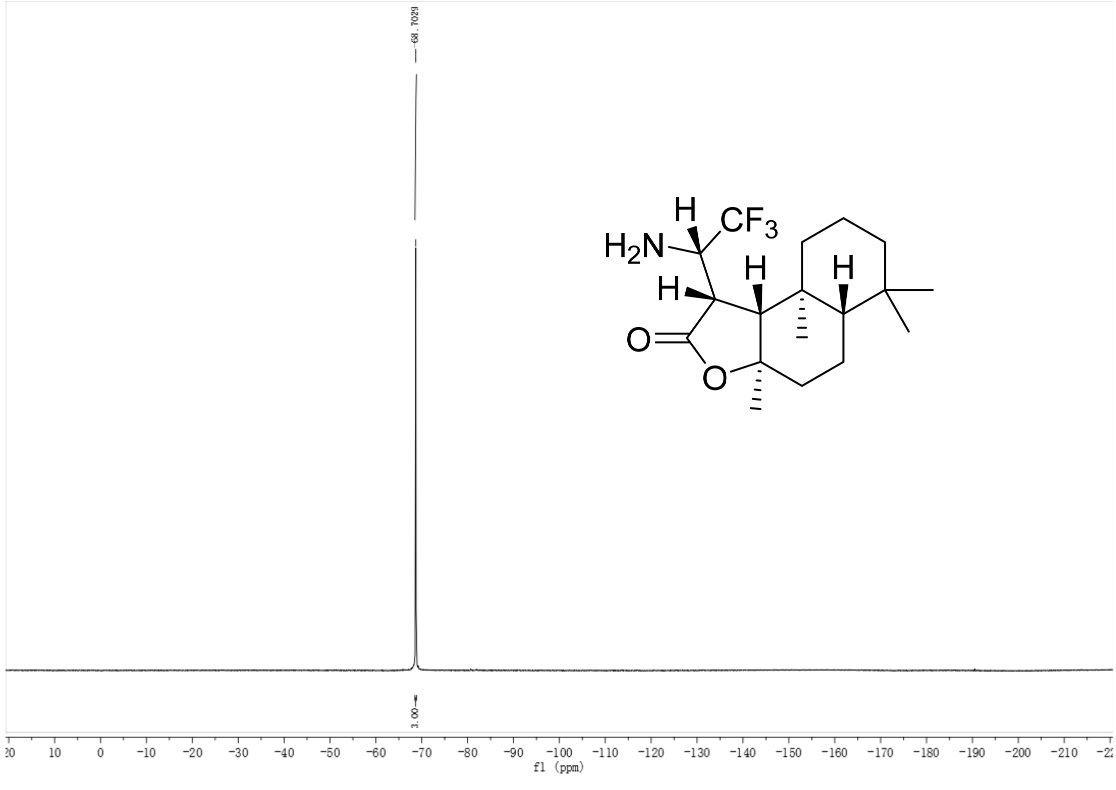

Supplement: Supplementary file 1 [file molecules-28-04067-s001.zip › molecules-2330799-supplementary.pdf]
